# Global burden and risk factors of gastritis and duodenitis: an observational trend study from 1990 to 2019

**DOI:** 10.1038/s41598-024-52936-1

**Published:** 2024-02-01

**Authors:** Yupei Liu, Jixiang Zhang, Yingyun Guo, Shan Tian, Yanrui Wu, Chuan Liu, Xiaoyu Huang, Shufei Zhang, Weiguo Dong

**Affiliations:** 1 Department of Gastroenterology, Renmin Hospital of Wuhan University, No 99 Zhangzhidong Road, WuhanHubei Province, 430060 China; 2 Department of Infection, Union Hospital of Tongji Medical College of Huazhong University of Science and Technology, Wuhan, 430022 China; 3 Department of Obstetrics and Gynecology, Renmin Hospital of Wuhan University, Wuhan, 430060 China

**Keywords:** Gastroenterology, Risk factors

## Abstract

In recent years, there has been a global trend of aging, which has resulted in significant changes to the burden of gastritis and duodenitis (GD). Using the global burden of disease (GBD) database spanning 1990 to 2019, we evaluated the temporal trends of age-standardized incidence rates (ASIR), age-standardized death rates (ASDR), and age-standardized disability-adjusted life years (AS-DALYs) for GD using estimated annual percentage changes (EAPC). Additionally, we examined the burden of GD across various strata, including social demographic index (SDI), age, and sex. Finally, the risk factors linked to the incidence and mortality of GD, utilizing Pearson correlation analysis. In 2019, there were 31 million GD patients globally, a notable increase of 12 million from 1990, while the ASIR, ASDR, and AS-DALYs for GD all showed a decrease. Correlation analysis showed a significant negative relationship between ASIR and SDI. Factors like hand hygiene and vitamin A deficiency had significant positive correlations with ASIR and ASDR in 2019. Over the past thirty years, the burden of GD has increased alongside global population aging. Future efforts should focus on exploring prevention for GD, with special attention to the elderly population in low SDI regions.

## Introduction

Gastritis and duodenitis (GD) are two chronic upper gastrointestinal diseases characterized by non-ulcerative damage to the lining of the stomach or duodenum primarily caused by infection with Helicobacter pylori (HP) or the use of non-steroidal anti-inflammatory drugs (NSAIDs)^1,2^. The clinical manifestations of GD include upper abdominal bloating, belching, vague abdominal pain, and postprandial discomfort. In severe cases, it can cause upper gastrointestinal bleeding, peritonitis, and perforation^3^. As GD progresses, duodenitis can progress in ulceration and obstruction in duodenum, greatly affecting the patients' health-related quality of life and psychological well-being. Additionally, gastritis carries the risk of malignant transformation. Professor Correa's proposed cascade model of intestinal-type gastric adenocarcinoma development suggests that the progression begins with the transformation of normal gastric mucosa into chronic non-atrophic gastritis, then progresses to chronic atrophic gastritis (CAG), followed by intestinal metaplasia (IM), and ultimately culminates in gastric cancer (GC)^4^. In a multicenter study conducted in Italy involving 668 patients with upper gastrointestinal symptoms who underwent gastroscopy, 30.1% of the patients were diagnosed with chronic gastritis^5^. Furthermore, a study in the Netherlands revealed that the annual incidence rates of GC among patients with AG and IM were 4.6% and 0.1%, respectively^6^. A Korean study found occurrence rates of AG and severe AG among diffuse GC patients to be 16.8% and 52.5%, respectively^7^. Therefore, preventing and controlling GD is of significant importance in preventing GC^8^. GD impose a considerable burden on public healthcare resources due to its chronic nature and high prevalence. Understanding epidemiological trends and strengthening the management of relevant risk factors are crucial in rationally allocating and utilizing global healthcare resources.

The GBD 2019 study represents a broad, global observational epidemiological study aimed at evaluating and tracking the health status of the entire human population, providing researchers and public health entities with summary data for policy and scientific purposes^9^. Within this study, the GBD 2019 dataset was utilized to examine the occurrence, fatality rates, and disability-adjusted life years (DALYs) attributed to GD from 1990 to 2019, categorized by gender, age, and levels of socioeconomic development. Additionally, we emphasized the discernible connections between environmental contamination, dietary patterns, inadequate sanitation facilities, and compromised water sources with the burden of GD. This study represents the inaugural endeavor to delineate the worldwide landscape of GD, thereby establishing a conceptual framework for a more profound comprehension of GD's impact on population health and paving the way for the formulation of global preventive strategies and the allocation of healthcare resources.

## Methods

### Overview

The Institute for Health Metrics and Evaluation (IHME) conducted the GBD 2019 study, which is a comprehensive analysis of the global burden of diseases, injuries, and risk factors. This study provides the most detailed insight into these health metrics worldwide. In total, the GBD 2019 study estimated 369 diseases and injuries, as well as 87 risk factors, across 204 countries and territories. Detailed information on the original data and general methodology of the GBD 2019 study has been previously published. The study protocol and statistical codes used to estimate GD can be accessed via the website: http://ghdx.healthdata.org/gbd-2019/code/cod-4. In summary, incidence and prevalence rates of diseases were estimated based on a wide range of data obtained from representative populations. These data were collected through literature reviews and research collaborations, including scientific reports of registries and cohorts, administrative health data, population surveys, and data from cohort and registry studies^10^.

### Data sources

Crude and age-standardized estimates of various measures of the burden of GD in 204 countries and territories from 1990 to 2019 and the respective 95% UIs were extracted from the GBD database via http://ghdx.healthdata.org/gbd-results-tool and no specific permissions were required to access data^10^. For the GBD 2019 assessment, GD was identified based on the following codes according to the 10th revision of the International Classification of Diseases (ICD-10): K29.0-K29.9 and R14 (S1 Table).

In the GBD 2019 study, population size was factored into calculations of age-standardized incidence rates (ASIR) , age-standardized death rates (ASDR) and age-standardized disability-adjusted life years (AS-DALYs) (per 100,000 population), and the 204 countries and territories were categorized into five groups based on their socio-demographic index (SDI): high, high-middle, middle, low-middle, and low SDI quintile^10^. The statistical analyses were performed using the following datasets:

1. The incidence, mortality, and DALYs numbers and rates of GD from 1990 to 2019 in global, SDI regions, and GBD regions;
2. The incidence, mortality, and DALYs numbers and rates of GD in different countries from 1990 to 2019;
3. The incidence, mortality, and DALYs numbers and rates of GD in different age groups and genders from 1990 to 2019;
4. The total population and rate of each country in the world from 1990 to 2019;
5. The SDI levels of different countries according to the World Health Organization (WHO) and the World Bank from 1990 to 2019;
6. The SEV of 69 (level IV) risk factors in GBD regions in 1990 and 2019.

### Definitions

GD were defined as death and disability resulting from damage and regeneration to lining of stomach or duodenum, with or without inflammation, but without ulceration. This can result from diverse aetiologies, but is primarily due to infection with HP or use of non-steroidal anti-inflammatory drugs in GBD database (https://www.healthdata.org/results/gbd_summaries/2019/gastritis-and-duodenitis-level-4-cause).

The socio-demographic index (SDI) is a composite measure of development that takes into account per capita income, educational attainment levels for individuals aged 15 years and above, and total fertility rates for individuals under the age of 25. The SDI ranges from 0 (representing low development) to 1 (representing high development), or can be categorized into five quintiles: low, low-middle, middle, high-middle, and high, based on their respective scores^11^.

Estimated annual percentage change (EAPC) is utilized to precisely assess the trend of age-standardized rates (ASR)^12^. EAPCs were computed through a linear regression model in the following manner: ln (ASR) = α + β x + ε, where x refers to the calendar year, and the ASR was obtained as follows:

$$ ASR = \frac{{ \mathop \sum \nolimits_{i = 1}^{A} aiwi }}{{\mathop \sum \nolimits_{i = 1}^{A} wi}} \times 10000 $$

In the *i*th age subgroup, ai is represented as age class. *wi* denotes the number of persons (or weight), where *i* is equal to the selected reference standard population^13^. when both the EAPC value and its 95% CI > 0, we consider its ASR to be on an upward trend; when both the EAPC value and 95% CI < 0, we consider its ASR to be on an upward trend; In other cases, we consider the ASR to be stable^14^.

The summary exposure value (SEV) is defined as "a metric that quantifies a population's exposure to a specific risk factor, incorporating both the magnitude of exposure and the severity of its impact on disease burden." It ranges from 0% (indicating no excessive risk exposure) to 100% (representing the highest level of risk exposure)^15^. The SEV is calculated by the following formula:

$$ SEV = \frac{{\int {\frac{u}{x = l}} RR\left( x \right)P\left( x \right)d\left( x \right) - 1}}{RRmax - 1} $$

where *RR(x)* is risk ratio at level *x* of exposure, *RRma*x is the highest risk ratio where more than 1% of population are exposed, *P(x)* is the density of exposure, and *l* and *u* are the lowest and highest levels of exposure, respectively.

### Statistical analysis

To assess the temporal burden of GD, ASR and their estimated percentage changes were calculated from 1990 to 2019 for incidences, deaths, DALYs, and EAPC in various countries and territories. The ASR values were presented per 100,000 population, along with their corresponding 95% uncertainty interval (95% UI), which were determined as the 2.5th and 97.5th ordered draws.

Furthermore, a hierarchy cluster analysis was conducted to categorize the countries and territories into 5 categories (a: Significantly decreased in ASDR; b: Significantly increased in ASIR; c: Significant increase; d: Minor increase; e: Minor decrease) based on the temporal trends in EAPC-ASIR and EAPC-ASDR of GD. Additionally, we present the global burden of GD at the national level and examine the association between sociodemographic status and ASIR, ASDR, and AS-DALYs. To identify potential risk factors contributing to the burden of GD. We assessed the relationship between the different risk factors in various GBD regions of summary exposure value (SEV) and the ASIR and ASDR of GD through Pearson correlation analysis. All data analyses were performed using the open-source software R (version 4.3.1, http://www.r-project.org/)^16^. A significance level of p < 0.05 was considered statistically significant for all analyses.

### Ethics statement

Ethics approval was exempted by the Ethics Committee of Wuhan University, because the GBD is a publicly available database and all participants’ data were anonymous.

## Results

### Distribution and trends in the incidence rate of GD by age or year

**Figure 1:**
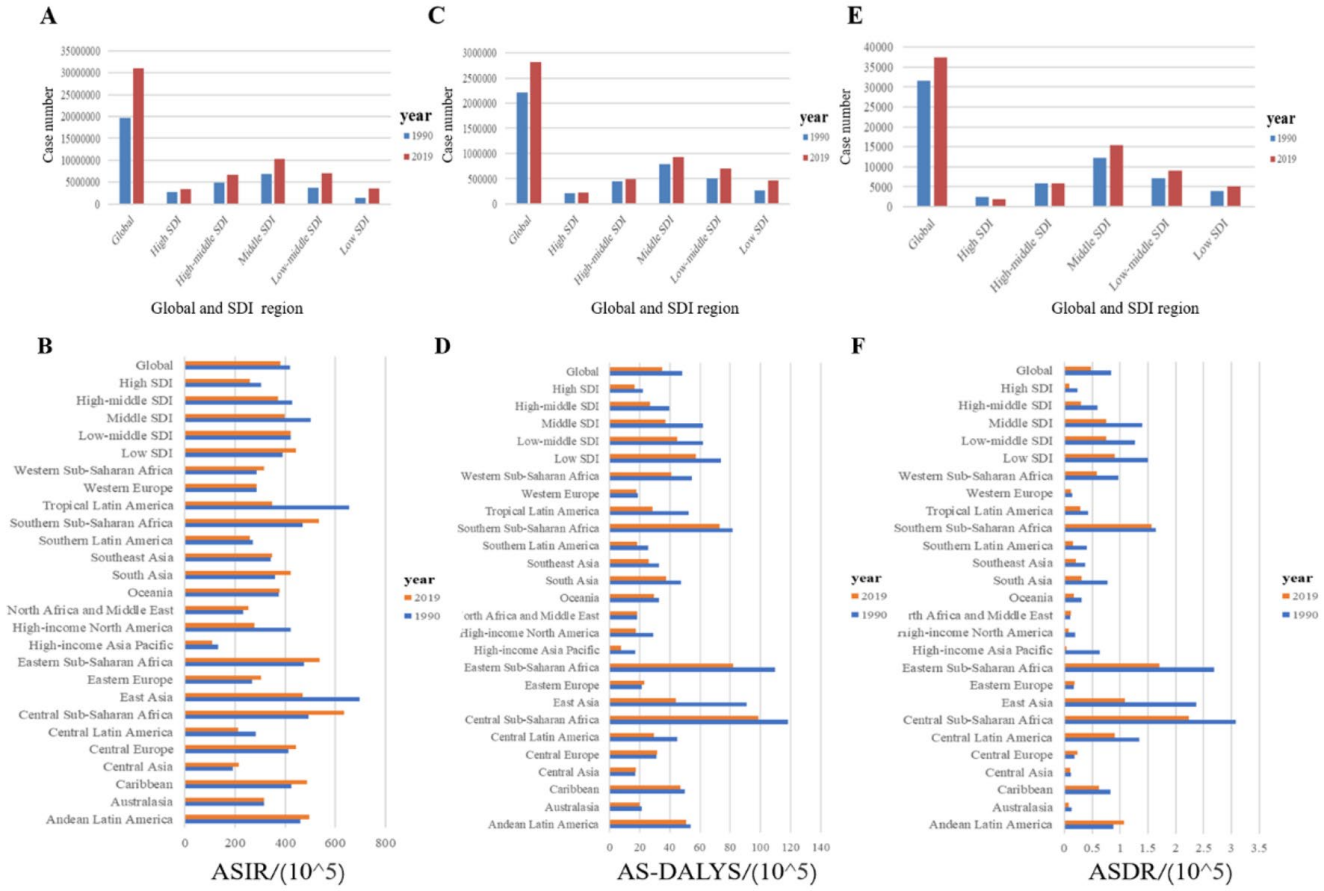
The number of incidences (**A**), DALYs (**C**) and deaths (**E**) in global and five SDI region and the ASIR (**B**), AS-DALYs (**D**) and ASDR (**F**) in 27 GBD region of GD over the past three decades. ASIR: age-standardized incidence rate; AS-DALYs: age-standardized disability-adjusted life years rate; ASDR: age-standardized death rate; SDI: socio-demographic index.

**Figure 2:**
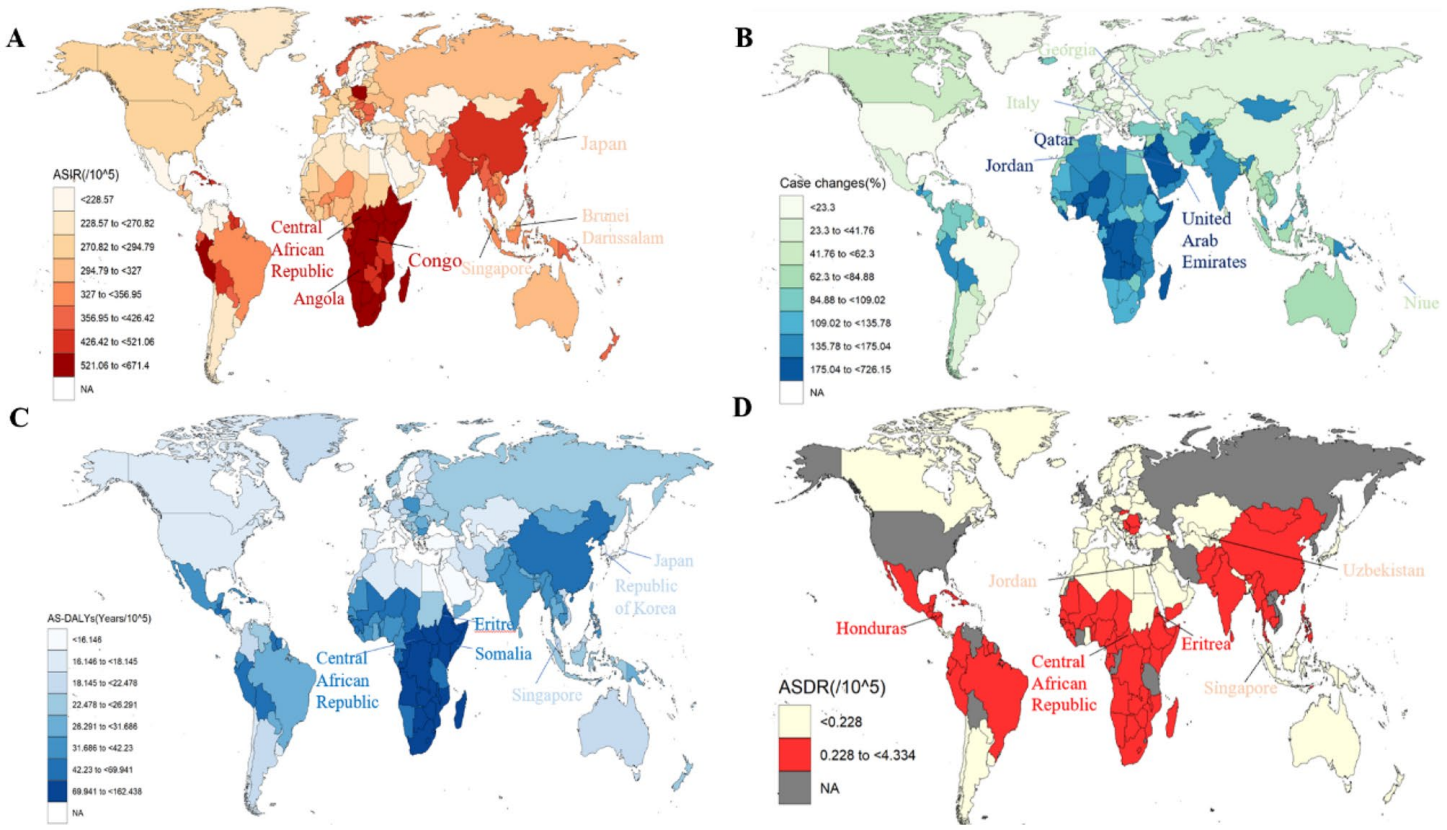
Distribution of ASIR (**A**), incidence case change (**B**), AS-DALYs (**C**) and ASDR (**D**) in 204 countries and territories. Note: All the world maps above were generated by open-source software R (version 4.3.1, http://www.r-project.org/). Abbreviation: ASIR: age-standardized incidence rate; AS-DALYs: age-standardized disability-adjusted life years rate; ASDR: age-standardized death rate.

At the global level, there were 30.9 million (95% UI: 25.4 to 36.7) incident cases of GD in 2019 (Fig. 1A and S2 Table), with an ASIR of 379.9/100,000 persons (95% UI:312.42 to 448.12) (Table 1). Over the 30-year period, only the low SDI region experienced an increasing ASIR for GD, whereas the ASIR in the other four SDI regions decreased. Among the 21 GBD regions, the Central Sub-Saharan Africa region showed the most significant increase in ASIR, while the Tropical Latin America region had the most significant decrease in ASIR (Fig. 1B and S3 Table). Examining the countries-level data, the three countries with the highest ASIR are Angola, Congo and Central African Republic, while the three countries with the lowest ASIR are Brunei Darussalam, Japan and Singapore (Fig. 2A).

**Table 1:** ASIR, ASDR and AS-DALYs of gastritis and duodenitis in 1990 and 2019 for global regions.

Regions	ASIR1990	ASIR2019	ASDR_1990	ASDR_2019	ASDALY_1990	ASDALY_2019
Global	419.58 (340.13 to 500.16)	379.88 (312.42 to 448.12)	0.83 (0.67 to 1.07)	0.48 (0.39 to 0.54)	48.14 (36.79 to 63.39)	34.78 (25.87 to 46.8)
**SDI region**						
High SDI	304.1 (240.96 to 372.02)	258.39 (212.23 to 307.71)	0.24 (0.2 to 0.26)	0.09 (0.08 to 0.12)	22.15 (15.22 to 31.36)	16.43 (10.98 to 23.56)
High-middle SDI	427.95 (346.57 to 515.49)	371.03 (304.54 to 439.12)	0.6 (0.46 to 0.81)	0.3 (0.26 to 0.34)	39.65 (29.07 to 54.14)	27.03 (18.95 to 37.71)
Middle SDI	502.14 (405.16 to 602.77)	398.19 (327.76 to 468.17)	1.4 (1.09 to 1.84)	0.75 (0.58 to 0.86)	61.94 (47.86 to 82)	37.26 (28.06 to 48.91)
Low-middle SDI	421.86 (346.9 to 497.47)	421.8 (346.48 to 497.76)	1.27 (0.94 to 1.84)	0.75 (0.63 to 0.88)	61.76 (46.49 to 82.09)	44.87 (34 to 59.5)
Low SDI	388.9 (318.89 to 460.38)	443.33 (360.79 to 525.55)	1.49 (0.88 to 2.17)	0.91 (0.57 to 1.18)	73.81 (52.28 to 97.23)	57.16 (42.75 to 74.7)
**GBD region**						
Andean Latin America	460.11 (376.37 to 553.07)	494.65 (411.79 to 581.23)	0.89 (0.65 to 1.39)	1.07 (0.79 to 1.39)	53.79 (39.39 to 72.98)	50.71 (38.32 to 66.17)
Australasia	315.11 (253.56 to 391.53)	315.09 (252.28 to 384.68)	0.13 (0.11 to 0.14)	0.07 (0.06 to 0.09)	21.4 (14.32 to 31.47)	19.92 (12.74 to 29.63)
Caribbean	424.07 (347.58 to 504.9)	485.15 (395.48 to 577.11)	0.83 (0.65 to 1.05)	0.62 (0.49 to 0.77)	49.87 (37.13 to 65.44)	46.72 (33.87 to 62.14)
Central Asia	189.38 (151.73 to 227.24)	214.9 (171.81 to 259.71)	0.12 (0.08 to 0.17)	0.1 (0.09 to 0.12)	17.09 (11.88 to 23.88)	17.33 (11.67 to 24.8)
Central Europe	413.62 (334.05 to 496.62)	443.45 (368.4 to 520.58)	0.19 (0.17 to 0.27)	0.24 (0.2 to 0.27)	31.21 (21.04 to 45.02)	31.46 (21.79 to 44.01)
Central Latin America	282.9 (231.68 to 335.97)	212.62 (176.19 to 250.08)	1.35 (1.22 to 1.45)	0.91 (0.77 to 1.06)	44.73 (38.08 to 53.55)	29.32 (24.23 to 35.64)
Central Sub-Saharan Africa	493.05 (403.36 to 591.83)	633.35 (511.12 to 762.97)	3.07 (1.68 to 4.43)	2.24 (1.18 to 3.26)	118.15 (78.49 to 159.67)	98.54 (67.56 to 132.39)
East Asia	697.3 (562.77 to 842.54)	467.88 (386.65 to 551.69)	2.36 (1.72 to 3.31)	1.08 (0.8 to 1.26)	90.77 (68.69 to 120.34)	43.96 (33.37 to 56.76)
Eastern Europe	266.31 (214.31 to 320.37)	302.24 (242.59 to 364.8)	0.17 (0.15 to 0.21)	0.18 (0.15 to 0.22)	21.19 (15 to 29.53)	23.06 (16.36 to 32.55)
Eastern Sub-Saharan Africa	473.44 (387.45 to 564.57)	537.36 (438.67 to 646.23)	2.69 (1.38 to 3.98)	1.71 (0.89 to 2.3)	109.9 (71.43 to 146.85)	81.75 (57.21 to 105.8)
High-income Asia Pacific	131.3 (105.72 to 158.64)	109.09 (89.07 to 131.44)	0.63 (0.35 to 0.72)	0.04 (0.03 to 0.08)	17.15 (12.77 to 21.69)	7.46 (4.9 to 10.92)
High-income North America	421.87 (335 to 519.62)	275.35 (233.68 to 320.45)	0.2 (0.16 to 0.21)	0.08 (0.07 to 0.1)	28.9 (19.39 to 41.57)	17.39 (11.64 to 24.55)
North Africa and Middle East	233.01 (185.99 to 283.25)	253.49 (201.48 to 306.9)	0.1 (0.07 to 0.15)	0.11 (0.08 to 0.13)	18.42 (12.33 to 26.87)	18.22 (12 to 26.65)
Oceania	373 (300.53 to 454.72)	375.93 (300.05 to 458.47)	0.31 (0.2 to 0.46)	0.16 (0.11 to 0.24)	33.03 (22.69 to 47.01)	29.57 (19.25 to 42.66)
South Asia	360.16 (294.62 to 426.58)	422.67 (344.69 to 503.13)	0.77 (0.44 to 1.31)	0.31 (0.21 to 0.43)	47.36 (33.6 to 65.21)	37.73 (26.26 to 52.97)
Southeast Asia	341.4 (272.87 to 415.94)	346.69 (275.66 to 422.77)	0.37 (0.28 to 0.47)	0.21 (0.17 to 0.25)	32.81 (23.63 to 44.64)	25.97 (17.67 to 37.11)
Southern Latin America	269.3 (217.74 to 324.54)	258.42 (205.53 to 311.27)	0.4 (0.3 to 0.49)	0.15 (0.13 to 0.18)	25.76 (19 to 34.51)	18.36 (12.65 to 26.38)
Southern Sub-Saharan Africa	469.65 (383.17 to 562.25)	534.5 (437.81 to 641.57)	1.65 (1.25 to 2)	1.56 (1.3 to 1.75)	81.58 (62.55 to 99.74)	72.81 (59.2 to 89.17)
Tropical Latin America	653.95 (533.12 to 778.68)	348.09 (287.44 to 414.65)	0.42 (0.38 to 0.46)	0.29 (0.25 to 0.32)	52.51 (36.33 to 74.29)	28.39 (20.4 to 39.45)
Western Europe	285.62 (224.79 to 353.55)	285.3 (227.28 to 350.74)	0.15 (0.13 to 0.16)	0.12 (0.1 to 0.13)	18.89 (12.69 to 27.26)	17.91 (11.91 to 25.9)
Western Sub-Saharan Africa	286.72 (233.58 to 341.62)	313.5 (252.71 to 379.03)	0.97 (0.51 to 1.58)	0.58 (0.34 to 0.85)	54.66 (36.11 to 78.57)	41 (30.28 to 55.48)

**Figure 3:**
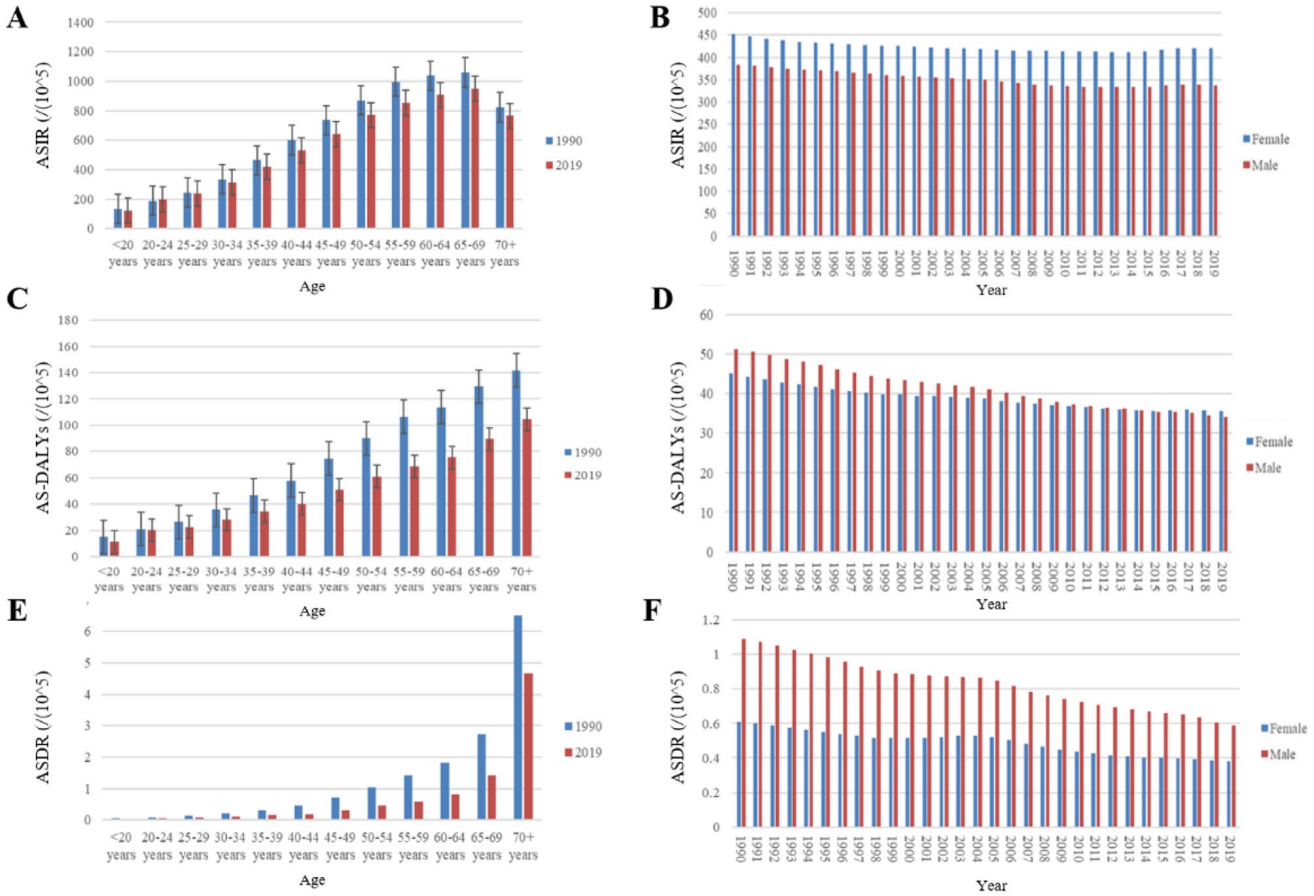
Distribution of incidence, DALYs and death rate in age distribution (**A, C, E**) and gender (**B, D, F**) from 1990–2019. Abbreviation: DALYs: disability-adjusted life years.

Despite the global decrease in ASIR for GD over the 30-year period, the total number of cases has increased. The number of cases in 2019 was 0.57 times higher than in 1990 (95% UI: 0.51 to 0.63), far exceeding the overall population growth globally (0.45 times) (S2 Table). Over the 30-year period, the Central Sub-Saharan Africa region has exhibited the most significant increase in the number of cases. Notably, only the High-income Asia Pacific and North America regions have experienced a decrease in the number of incidence cases by 0.04 times (95% UI: -0.11 to 0.03) and 0.02 times (95% UI: -0.12 to 0.09), respectively. At the country level, Qatar, United Arab Emirates, and Jordan have shown the most significant increase in the number of cases, while Italy, Niue, and Georgia had the most significant decrease (Fig. 2B and S4 Table). In the age-group analysis over the past 30 years, the ASIR of GD increases with age and peaks between 60 to 70 years old before declining. In gender-group analysis, females consistently exhibit higher incidence rates of GD than males (Fig. 3A, B).

### Distribution and trends in the Dalys rate of GD by age or year

At the global level, the number of DALYs due to GD was estimated to be 2.22 million (95% UI: 1.69 to 2.92) in 1990 and increased to 2.82 million (95% UI: 2.10 to 3.7) in 2019(Fig. 1C). Over the past 30 years, the AS-DALYs rate decreased from 48.14/100,000 persons (95% UI, 36.79 to 63.39) in 1990 to 34.78/100,000 persons (95% UI:25.87 to 46.8) in 2019 (Table 1 and S5 Table). However, the number of DALYS increased in all five SDI regions, with the greatest increases observed in the low and low-middle SDI regions.

Among the other 21 GBD regions, the most significant decreases in AS-DALYs were observed in the East Asia and Tropical Latin America regions over the past three decades (Fig. 1D and Table 1). At the nation level, currently, the three countries most affected by AS-DALYs are Central African Republic, Eritrea, and Somalia, while the three countries least affected are Japan, Singapore, and Republic of Korea (Fig. 2C). In comparison to 1990, the three countries with the most significant decrease in GD-related AS-DALYs are China, Equatorial Guinea, and Ethiopia, while Serbia, Bulgaria, and Lithuania have experienced the greatest increase (S6 Table).

In age-stratified analysis, AS-DALYS increases with advancing age. In gender-stratified analysis, male AS-DALYs have consistently been higher than female since 1990. However, since 2015, female AS-DALYs have started to surpass male (Fig. 3C, D). Further analysis of age groups reveals that this trend is particularly prominent in the age ranges of 20–40 and 60–70(S2 Fig C).

### Distribution and trends in the deaths rate of GD by age or year

As mentioned previously, in general, GD does not typically lead to severe consequences. It only poses a threat to the patient's life and health when it progresses to bleeding or malignancy. Over the past 30 years, as the global incidence of GD has increased, resulting in a rise in its death toll, with 37,492 deaths attributed to GD worldwide in 2019 (Fig. 1E). This represents a 19% increase from 1990. It is noteworthy that despite the increasing number of GD death cases, its ASDR is declining, falling from 0.83 per 100,000 people in 1990 to 0.48 per 1000,000 people in 2019 (Fig. 1F and Table 1).

Among the five SDI regions, the Middle SDI region had the highest number of death cases, while the low-SDI regions have the highest ASDR (S7 Table). At the nation level, in 2019, the top three countries with the highest ASDR were Central African Republic, Honduras, and Eritrea. The three countries with the lowest ASDR were Jordan, Singapore, and Uzbekistan (Fig. 2D and S8 Table).

In age and gender stratified analysis, the ASDR of GD increases with advancing age, and it consistently remains higher in males compared to females. Fortunately, there has been significant improvement in the burden of GD over the past 30 years. Particularly, the ASDR for males has decreased by approximately half compared to 1990 (Fig. 3E, F).

### EAPC of GD related ASIR, AS-DALYs, and ASDR and cluster analysis

Over the past three decades, the incidence, DALYs and mortality associated with GD have all continued to rise, while the global ASIR, ASDR and AS-DALYs related to GD have consistently shown a downward trend.

When examining the EAPC of GD burden in five SDI regions, we find that the ASDR and AS-DALYs is on a downward trend (S5 Table and S7 Table). However, the ASIR is increasing in the Low-middle and low SDI regions (S3 Table). On a more granular level, among the 21 GBD regions, South Asia (EAPC = 0.81,95%UI: 0.6 to 1.01) and Tropical Latin America (EAPC = -2.53,95%UI: -2.77 to -2.29) have experienced the most significant increase and decrease in ASIR, respectively. Central Europe (EAPC = 0.25,95%UI: 0.19 to 0.32) and High-income Asia Pacific (EAPC = -3.5,95%UI: -3.95 to -3.05) have seen the most substantial increase and decrease in AS-DALYs, respectively. Andean Latin America (EAPC = 1.8, 95%UI: 1.3 to 2.31) and High-income Asia Pacific (EAPC = -11.42, 95%UI: -12.46 to -10.37) have experienced the most significant increase and decrease in ASDR, respectively.

At the national level, Serbia (EAPC = 1.53, 95%UI:1.33 to 1.72) and Brazil (EAPC = -2.57, 95%UI: -2.82 to -2.32) have seen the most significant increase and decrease in ASIR (S4 Table). Serbia (EAPC = 1.95,95%UI:1.68 to 2.23) and Republic of Korea (EAPC = -7.9 ,95%UI: -8.83 to -6.95) have experienced the most significant increase and decrease in AS-DALYs (S6 Table). Croatia (EAPC = 6.56,95%UI: 5.79 to 7.32) and Japan (EAPC = -9.53,95%UI: -10.44 to -8.62) have seen the most substantial increase and decrease in ASDR (S8 Table).

**Figure 4:**
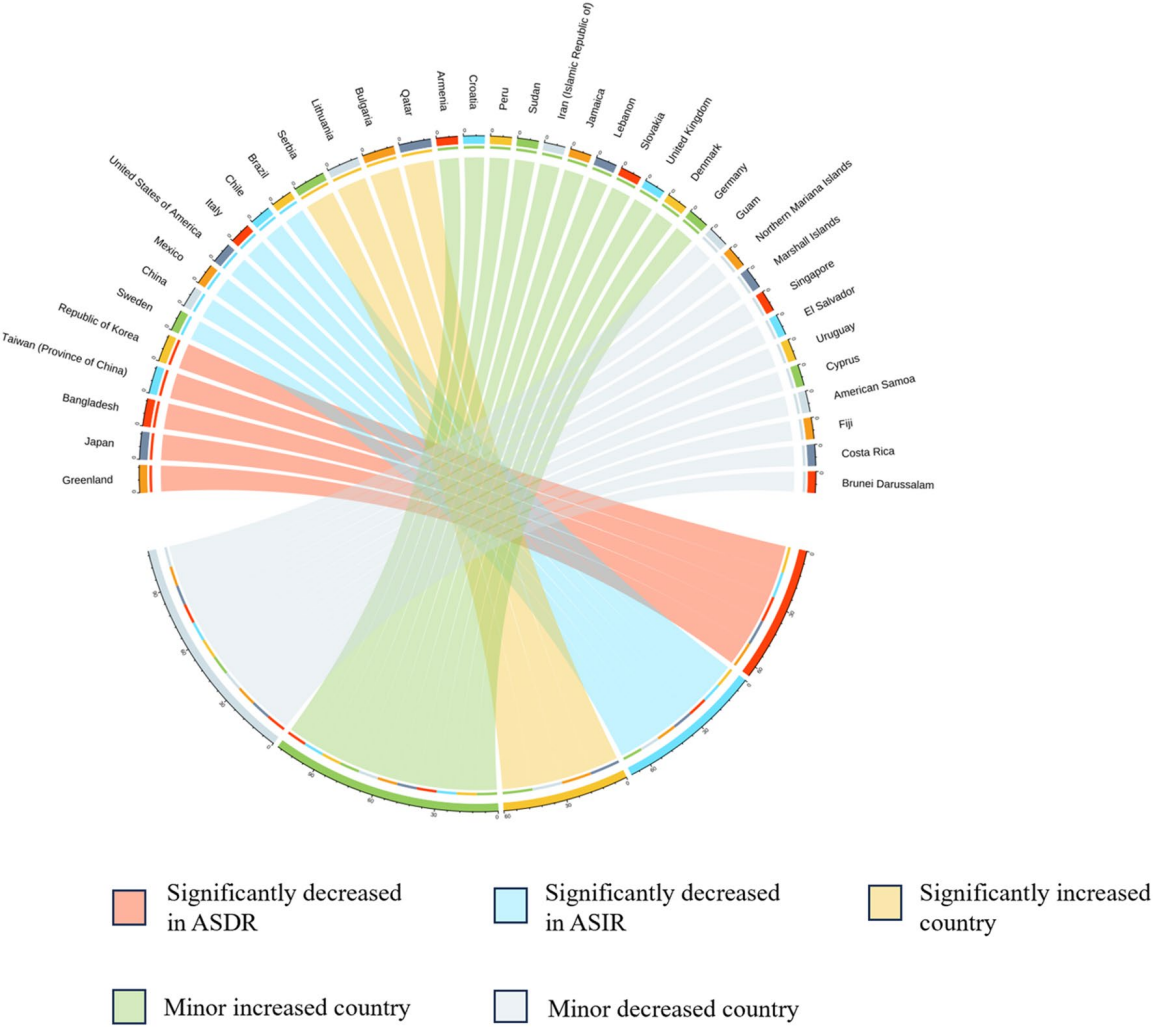
Cluster analysis of EAPC-ASIR and EAPC-ASDR in different countries. (the minor increased and decreased country only display ten countries). Abbreviation: EAPC-ASIR: estimated annual percentage changes in age-standardized incidence rates. EAPC-ASDR: estimated annual percentage changes in age-standardized death rates.

Additionally, we conducted cluster analysis of EAPC in ASDR and ASIR among 204 countries, resulting in the classification of these countries into five clusters. South Korea and Japan were placed in the significant decrease group for ASDR, while China and Italy were categorized in the significant decrease group for ASIR. Serbia and Qatar were assigned to the significant increase group, while 81 countries including Greenland and Sudan were grouped in the category of minor increase. Finally, 107 countries including Fiji and Singapore were placed in the minor decrease group (Fig. 4).

## Correlation analysis of GD related ASIR, ASDR, AS-DALYs and different SDI

**Figure 5:**
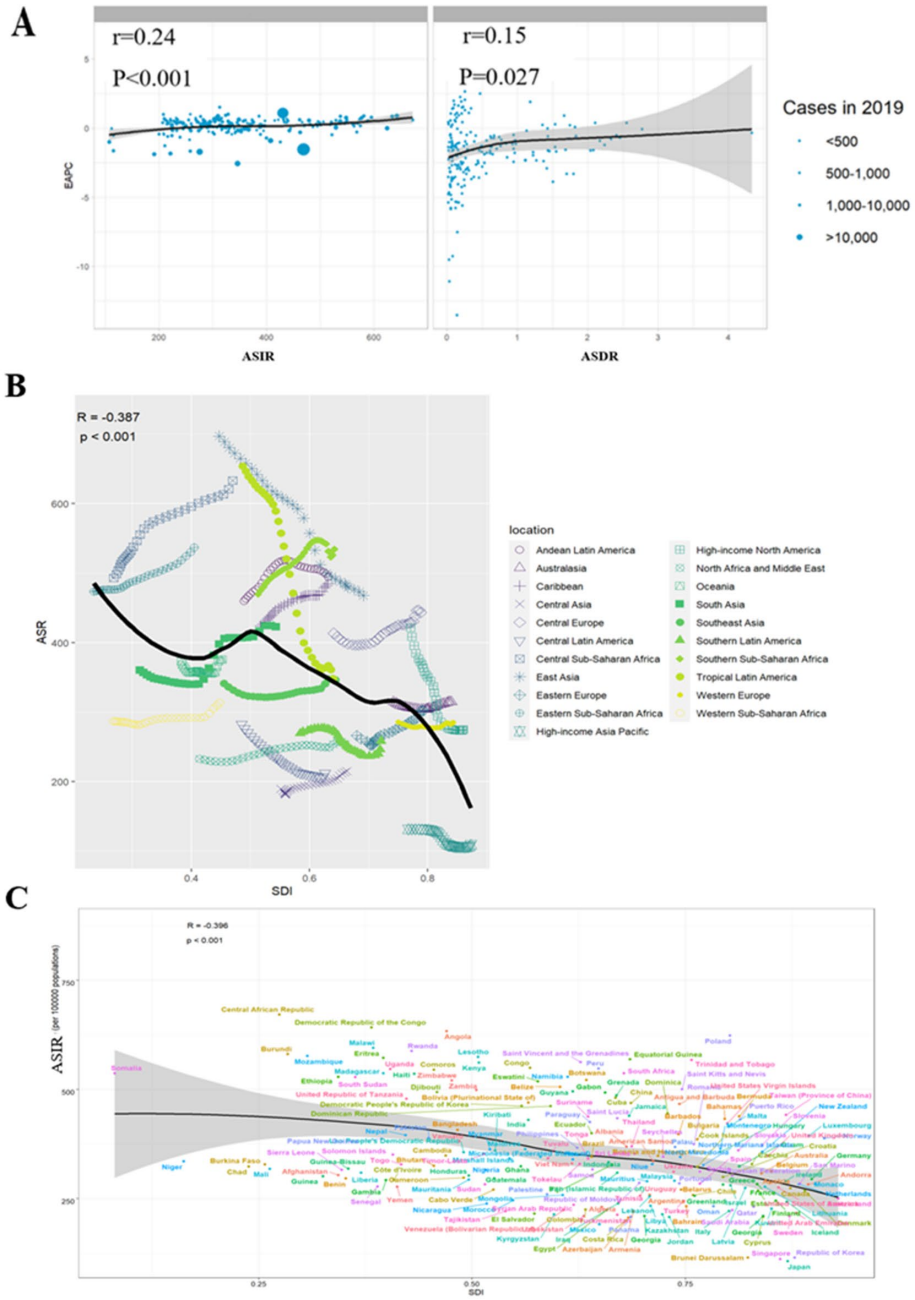
Correlation analysis. (**A**) The correlation between EAPC and ASIR/ASDR in 2019. (**B**) ASIR for GD for different regions and (**C**) countries and territories by SDI, 1990–2019. Abbreviation: EAPC: estimated annual percentage changes. ASIR: age-standardized incidence rate; GD: gastritis and duodenitis; SDI: social demographic index.

As shown in Fig. 5A, a significant correlation between EAPC and ASIR in 2019 (r = 0.24, p < 0.001) as well as between EAPC and ASDR (r = 0.15, p < 0.027).

A correlation between different SDI regions and their expected level of ASIR has been observed over the past three decades (r = -0.387, P < 0.001). A predicted curve was plotted, and it was observed that most GBD regions followed the trend of the curve, although some GBD regions deviated considerably from it, such as the High-income Asia Pacific being much higher than the expected level, and East Asia being much lower (Fig. 5B). In most regions, as SDI increases, ASIR gradually stabilizes or decreases. However, some regions, such as Central Sub-Saharan Africa and Southern Sub-Saharan Africa, experience significant fluctuations in ASIR. Figure 5C provides a detailed account of the correlation between SDI and ASIR for each country.

Furthermore, significant negative correlations have been found between ASDR (r = -0.548, p < 0.001) and AS-DALYs (r = -0.617, p < 0.001) with SDI values. ASDRs in most high SDI regions are limited to a very low level, almost zero. However, ASDRs are higher than expected in some parts of Southern Sub-Saharan Africa regions, and AS-DALYs are highly fluctuating (S3A and S4A Figure). Further details on the correlation between ASDR, AS-DALYs, and SDI for each country are presented in Supplementary Fig. 3B and 4B.

### GD-related risk factors

To further investigate the risk factors associated with ASIR and ASDR of GD, we conducted a correlation analysis between the SEV of 69 risk factors extracted from the GBD database in 21 GBD regions and their corresponding ASIR and ASDR. The results revealed significant positive correlations between ASIR in 1990 and SEV of occupational particulate matter, gases, and fumes, as well as a diet low in fruits. Additionally, ASDR in 1990 was significantly associated with SEV of high BMI index and occupational exposure to trichloroethylene (S9 Table). Furthermore, ASIR and ASDR in 2019 were found to be significantly correlated with factors such as SEV of vitamin A deficiency and no access to handwashing facility (Table 2). Supplementary Table 10 provides the detailed information of risk factors in the GBD database.

**Table 2:** Risk factors associated with ASIR and ASDR of GD in 2019.

Risk factor	ASIR		ASDR	
	r	P	r	P
Lead exposure	0.541*	0.011	0.519*	0.016
Child and maternal malnutrition	0.490*	0.024	0.504*	0.02
Iron deficiency	0.508*	0.019	0.493*	0.023
Vitamin A deficiency	0.621**	0.003	0.712**	< 0.001
Zinc deficiency	0.466*	0.033	0.440*	0.046
Unsafe water source	0.593**	0.005	0.570**	0.007
Unsafe sanitation	0.526*	0.014	0.565**	0.008
No access to handwashing facility	0.629**	0.002	0.740**	< 0.001
Household air pollution from solid fuels	0.519*	0.016	0.552**	0.009
Low bone mineral density	0.440*	0.046	0.644**	0.002
Diet low in vegetables	0.588**	0.005	0.588**	0.005
Diet low in milk	0.427	0.054	0.493*	0.023
Diet low in seafood omega-3 fatty acids	0.605**	0.004	0.467*	0.033
Occupational particulate matter, gases, and fumes	0.403	0.07	0.513*	0.017
Occupational noise	0.425	0.055	0.572**	0.007
Occupational ergonomic factors	0.422	0.057	0.531*	0.013
Intimate partner violence	0.35	0.12	0.457*	0.037
Diet low in calcium	0.545*	0.011	0.630**	0.002
Occupational exposure to beryllium	0.354	0.115	0.465*	0.034
Occupational exposure to cadmium	0.338	0.135	0.462*	0.035
Occupational exposure to formaldehyde	0.348	0.123	0.500*	0.021
Occupational exposure to trichloroethylene	0.314	0.166	0.436*	0.048
Child stunting	0.464*	0.034	0.453*	0.039
Particulate matter pollution	0.519*	0.016	0.560**	0.008

ASIR: ASIR: age-standardized incidence rate; ASDR: ASIR: age-standardized death rate; r: correlation coefficient; P: p-value.

## Discussion

Based on the latest authoritative evidence from the GBD database 2019, this study utilized statistical parameters (ASIR, ASDR, AS-DALYs) to comprehensively analyze and depict the global epidemiology of GD for the first time, filling the gap in GD-related epidemiology in recent years.

Our findings suggest that there has been a significant increase in the number of GD cases over a period of 30 years, from 19.8 million in 1990 to 31 million in 2019, indicating a growth rate of 56.93%. This growth rate exceeds the global population growth rate during the same time period, suggesting that factors beyond population migration and growth contribute to the rise in GD incidence. Analysis of data from the MarketScan Commercial Claims and Encounters and Medicare Supplemental database demonstrates a consistent upward trend in the utilization of endoscopic examinations over the past two decades. With the exception of patients aged 75 and above who may have contraindications for certain examinations, endoscopic examination rates have shown a steady increase across various age groups. Notably, the examination rate for upper gastrointestinal endoscopy has increased by more than 5% for individuals under 60 years old and by over 10% for the 60–65 age group^17^. The widespread adoption of endoscopic technology has significantly contributed to the increased detection rates of GD, as it serves as a primary diagnostic tool. Consequently, this has led to a rise in the number of GD cases. Furthermore, we propose that the escalation in GD cases may be partially attributed to variations in SDI levels. While SDI levels have globally increased over the past three decades, underdeveloped regions have not witnessed proportional improvements in socio-economic factors alongside population growth. SDI provides a comprehensive overview of a country's education, economy, and health indicators. Low SDI levels are associated with various influences, including limited awareness of GD^18^, low fruit and vegetable consumption^19^, poor awareness of hand hygiene^20^, and infrequent handwashing^18^ – all of which are significantly linked to the infection rate of HP^21^. Consequently, there is a notable disparity in HP infection rates between countries, such as Oceania (24.4%) and Africa (79.1%)^22^. Given that HP is a major driver of GD incidence, it has played a significant role in promoting the occurrence of GD.

Additionally, our statistical analysis reveals a significant correlation between SDI and the burden of GD. Our findings indicate that regions with low SDI levels exhibit higher ASIR, ASDR and AS-DALYs, particularly in terms of ASDR, where the difference approaches almost tenfold. Furthermore, changes in SDI also lead to variations in the burden of GD. Over the 30-year period, changes in the SDI index have been associated with shifts in the regions experiencing the highest ASIR for GD, transitioning from East Asia in 1990 to Central Sub-Saharan Africa in 2019. ASDR and ASDALYs have consistently remained highest in Central Sub-Saharan Africa. At the national level, the burden of GD, influenced by implications stemming from economic underdevelopment, is notably concentrated in countries with high rankings in ASIR, ASDR, and ASDALYs, predominantly located in the Central Sub-Saharan Africa region, including the Central African Republic, Congo, and Somalia.

Additionally, due to limited access to treatment, DALYs in less developed countries can be significantly higher than those in more developed nations. Consequently, there exists a substantial discrepancy in disability and mortality rates when compared to developed countries. For example, delayed access to healthcare resulting from inadequate health insurance coverage often leads to the presentation of more advanced disease at the time of initial diagnosis, subsequently increasing the risk of complications and mortality. The results of the EAPC for the global burden of GD reveal the ASIR, ASDR and AS-DALYs demonstrate a downward trend across most regions. Encouragingly, this trend is also significant in numerous developing countries, where both the incidence and mortality rates of GD are progressively decreasing. Although the extent of improvement may not be as pronounced as in larger developing countries such as China, overall trends are showing steady improvement. It is anticipated that continued economic development, advancements in healthcare and education, and progress in GD disease management systems will contribute to a sustained decline in the mortality and disability rates of GD in developing countries.

Furthermore, in our analysis of age and gender, we observed that the ASIR of GD reaches its peak and subsequently declines within the 60–70 age group. This age-related pattern closely aligns with two etiologies documented in the GBD database: HP infection and the use of NSAID medications. Notably, A multicenter study from Korea revealed a linear increase in the seropositivity rate of HP antibodies from the 30–39 age group to the 60–69 age group, with lower infection rates observed in the 20–29 age group and among individuals aged 70 and above^23^. Similarly, retrospective studies from Germany indicated that the peak usage of NSAIDs medications occurs around the age of 50, decreasing in individuals aged 70 and above due to challenges in tolerating associated medication side effects^24,25^. Regarding gender stratification, our findings indicate a higher ASIR in females compared to males, a trend akin to the incidence of gastroesophageal reflux disease^26^. Conversely, the ASDR and AS-DALYs tend to be higher in males, reflecting a general trend in the burden of digestive system diseases^27^. We posit that this gender disparity may be linked to male drinking behavior, which exacerbates the prognosis of gastric diseases. However, it is noteworthy that since 2015, AS-DALYs in females have begun to surpass those in males, a trend that has persisted. Similar evidence was documented in a study by Karaye et al., indicating a greater increase in alcohol-related deaths in women in recent years compared to men in the United States, reflecting shifts in female drinking patterns characterized by increased frequency and quantity of alcohol consumption compared to the past^28^.

In our risk factor analysis, we identified vitamin A deficiency and lack of access to handwashing facilities as the primary risk factors associated with the ASIR and ASDR for GD in 2019. It is widely recognized that vitamin B12 deficiency is a risk factor for atrophic gastritis^29^, underscoring the impact of vitamins on the onset and progression of gastrointestinal diseases. Moreover, previous studies have demonstrated that vitamin A, serving as an antioxidant, exerts beneficial effects on the gastrointestinal tract by attenuating oxidative stress during disease progression and modulating intestinal microbiota^30^.

In relation to the impact of handwashing facilities on GD, a cluster randomized trial conducted in Spain demonstrated significant effectiveness of a hand hygiene program in reducing acute gastroenteritis at child care centers, particularly during the winter^31^. Studies from the Cochrane Library indicate that promoting handwashing can reduce diarrhea episodes by approximately 30% in child day-care centers in high-income countries and among communities in low- and middle-income countries^32^. Furthermore, the absence of handwashing facilities may be linked to the level of development in a given country, indirectly suggesting that countries with lower SDI exhibit higher ASDR and ASIR for GD. As previously mentioned, this risk factor primarily impacts children and adolescents through HP infection, and with increasing duration of HP infection and the influence of environmental factors, the incidence of GD steadily rises until reaching a peak in middle age.

This study represents the first comprehensive exploration of the global burden of gastritis over a 30-year period, encompassing 27 regions and 204 countries. We conducted an assessment of the worldwide burden of gastritis, along with its gender and age disparities, utilizing ASIR, ASDR, AS-DALYs. Furthermore, we evaluated the changes in the global burden of GD over the 30-year period using the EAPC, providing a more accurate and comprehensive understanding of GD's impact on the global population. Additionally, we employed the EAPC to evaluate the changes in the global burden of gastritis over the 30-year period, thereby offering a more precise and thorough comprehension of gastritis’ impact on the global population.

Nevertheless, it is crucial to acknowledge the inherent limitations of our study. Firstly, the diverse prognoses associated with different types of gastritis are not accounted for in the GBD database, which lacks detailed classification for gastritis. This limitation may introduce heterogeneity and necessitate cautious interpretation of certain results. Secondly, the estimates of disease burden in the GBD rely on mathematical modeling, drawing from limited data sources obtained from epidemiological surveys. Consequently, these estimates may exhibit some degree of deviation from actual data, particularly in countries with substantial aging populations and in underdeveloped regions with scarce prior information. Lastly, the GBD encompasses a restricted number of risk factors, limiting our ability to explore the correlation between existing risk factors in the database and the burden of gastritis. This inevitably results in overlooking the impact of certain significant factors on the burden of gastritis.

## Conclusion

Due to the global trend of population aging, there is a continuous increase in the incidence, mortality, and disability caused by GD, leading to a significant impact on global healthcare resource consumption. Although ASIR, ASDR and AS-DALYs for GD have been gradually decreasing worldwide, high ASDR and AS-DALYs persist in certain underdeveloped regions. Furthermore, given the chronic recurrent nature of GD, it remains a significant influencing factor on patients' quality of life and the progression of gastric cancer. Therefore, it is crucial to prioritize the exploration of preventive and treatment methods for GD in the future, especially for elderly populations in low-SDI region.

### Supplementary Information

Supplementary Information.

## Data Availability

The datasets presented in this study can be found in online (http://ghdx.healthdata.org/gbd-results-tool). Further information can be directed to the corresponding author.
