# Supplementary material for: Global burden and risk factors of gastritis and duodenitis: an observational trend study from 1990 to 2019
Source: Sci Rep. 2024 Feb 1;14:2697. doi: 10.1038/s41598-024-52936-1 (PMC10834532; doi:10.1038/s41598-024-52936-1)
Supplement: Supplementary file 1 — Supplementary Information. [file 41598_2024_52936_MOESM1_ESM.docx]

**Global burden and risk factor of gastritis and duodenitis: An observational trend study from 1990 to 2019**

**Supplementary Materials.**

**Supplementary table 1: The definition of GD in ICD-10.**

| ICD-10 codes | Conditions |
| --- | --- |
| K29 | Gastritis and duodenitis |
| K29.0 | Acute haemorrhagic gastritis |
| K29.1 | Other acute gastritis |
| K29.2 | Alcoholic gastritis |
| K29.3 | Chronic superficial gastritis |
| K29.4 | Chronic atrophic gastritis |
| K29.5 | Chronic gastritis, unspecified |
| K29.6 | Other gastritis |
| K29.7 | Gastritis, unspecified |
| K29.8 | Duodenitis |
| K29.9 | Gastroduodenitis, unspecified |
| R14 | Flatulence and related conditions |

Abbreviations: ICD-10, 10th revision of the International Classification of Diseases;

**Supplementary table 2: Changes in global population and incidence of GD over 30 years**

| Location | Total population | | Growth ratio（%） | GD population | | Growth ratio（%） |
| --- | --- | --- | --- | --- | --- | --- |
|  | 1990 | 2019 |  | 1990 | 2019 |  |
| Global | 5.35X10^9^ | 7.74 X10^9^ | 44.63 | 1.97 X10^7^ | 3.10 X10^7^ | 56.93 |
| Qatar | 4.45X10^5^ | 2.86X10^6^ | 543.53 | 8.00X10^2^ | 6.5 X10^3^ | 712.50 |
| United Arab Emirates | 1.87X10^6^ | 9..24X10^6^ | 393.66 | 3.30 X10^3^ | 2.39X10^4^ | 624.24 |
| Jordan | 3.77X10^6^ | 1.16 X10^7^ | 208.41 | 4.70 X10^3^ | 2.18 X10^4^ | 363.83 |
| Italy | 5.68 X10^7^ | 6.03 X10^7^ | 6.19 | 2.46 X10^5^ | 1.89 X10^5^ | -23.11 |
| Niue | 2.33 X10^3^ | 1.67 X10^3^ | -28.15 | 7.00 | 6.00 | -14.29 |
| Georgia | 6.61 X10^6^ | 1.07 X10^7^ | 61.42 | 1.07 X10^4^ | 9.30 X10^3^ | -13.08 |

Note: The countries shown in the table are the top 3 countries with the most significant increase and decrease in GD cases over 30 years.

**Supplementary table 3: Incidence of gastritis and duodenitis in 1990 and 2019 for GBD region, with EAPC from 1990 and 2019.**

| location | Num_1990(x10^3^) | ASR_1990/10^5^ | Num_2019(x10^3^) | ASR_2019/10^5^ | Num_change | EAPC_CI |
| --- | --- | --- | --- | --- | --- | --- |
| **Global** | 19753 (16066 to 23437) | 419.58 (340.13 to 500.16) | 30998 (25440 to 36675) | 379.88 (312.42 to 448.12) | 0.57 (0.51 to 0.63) | -0.36 (-0.42 to -0.3) |
| **SDI region** |  |  |  |  |  |  |
| High SDI | 2821 (2256 to 3462) | 304.1 (240.96 to 372.02) | 3432 (2783 to 4120) | 258.39 (212.23 to 307.71) | 0.22 (0.15 to 0.29) | -0.61 (-0.71 to -0.52) |
| High-middle SDI | 4874 (3937 to 5837) | 427.95 (346.57 to 515.49) | 6688 (5405 to 8034) | 371.03 (304.54 to 439.12) | 0.37 (0.29 to 0.45) | -0.57 (-0.63 to -0.51) |
| Middle SDI | 6906 (5591 to 8225) | 502.14 (405.16 to 602.77) | 10336 (8449 to 12272) | 398.19 (327.76 to 468.17) | 0.42 (0.34 to 0.51) | -0.83 (-0.88 to -0.78) |
| Low-middle SDI | 3710 (3042 to 4370) | 421.86 (346.9 to 497.47) | 6951 (5683 to 8221) | 421.8 (346.48 to 497.76) | 0.86 (0.79 to 0.92) | 0.07 (-0.02 to 0.16) |
| Low SDI | 1433 (1175 to 1682) | 388.9 (318.89 to 460.38) | 3572 (2906 to 4237) | 443.33 (360.79 to 525.55) | 1.38 (1.32 to 1.43) | 0.49 (0.42 to 0.56) |
| **GBD region** |  |  |  |  |  |  |
| Andean Latin America | 135 (110 to 162) | 460.11 (376.37 to 553.07) | 302 (251 to 356) | 494.65 (411.79 to 581.23) | 1.24 (1.08 to 1.4) | 0.28 (0.16 to 0.39) |
| Australasia | 69 (56 to 87) | 315.11 (253.56 to 391.53) | 110 (88 to 134) | 315.09 (252.28 to 384.68) | 0.59 (0.5 to 0.69) | 0.04 (-0.01 to 0.09) |
| Caribbean | 129 (106 to 152) | 424.07 (347.58 to 504.9) | 244 (198 to 291) | 485.15 (395.48 to 577.11) | 0.89 (0.78 to 1) | 0.41 (0.37 to 0.45) |
| Central Asia | 114 (92 to 136) | 189.38 (151.73 to 227.24) | 198 (158 to 240) | 214.9 (171.81 to 259.71) | 0.74 (0.64 to 0.82) | 0.53 (0.46 to 0.59) |
| Central Europe | 559 (448 to 675) | 413.62 (334.05 to 496.62) | 686 (563 to 821) | 443.45 (368.4 to 520.58) | 0.23 (0.15 to 0.32) | 0.39 (0.31 to 0.46) |
| Central Latin America | 333 (272 to 391) | 282.9 (231.68 to 335.97) | 528 (436 to 621) | 212.62 (176.19 to 250.08) | 0.58 (0.48 to 0.68) | -1.06 (-1.17 to -0.96) |
| Central Sub-Saharan Africa | 161 (132 to 189) | 493.05 (403.36 to 591.83) | 492 (395 to 587) | 633.35 (511.12 to 762.97) | 2.06 (1.93 to 2.21) | 0.84 (0.81 to 0.88) |
| East Asia | 7332 (5913 to 8875) | 697.3 (562.77 to 842.54) | 9289 (7532 to 11009) | 467.88 (386.65 to 551.69) | 0.27 (0.17 to 0.38) | -1.52 (-1.62 to -1.41) |
| Eastern Europe | 674 (537 to 813) | 266.31 (214.31 to 320.37) | 793 (624 to 969) | 302.24 (242.59 to 364.8) | 0.18 (0.13 to 0.22) | 0.62 (0.54 to 0.71) |
| Eastern Sub-Saharan Africa | 541 (448 to 636) | 473.44 (387.45 to 564.57) | 1359 (1110 to 1611) | 537.36 (438.67 to 646.23) | 1.51 (1.45 to 1.58) | 0.4 (0.35 to 0.45) |
| High-income Asia Pacific | 242 (195 to 294) | 131.3 (105.72 to 158.64) | 232 (189 to 281) | 109.09 (89.07 to 131.44) | -0.04 (-0.11 to 0.03) | -1 (-1.15 to -0.85) |
| High-income North America | 1324 (1059 to 1639) | 421.87 (335 to 519.62) | 1291 (1077 to 1522) | 275.35 (233.68 to 320.45) | -0.02 (-0.12 to 0.09) | -1.6 (-1.74 to -1.45) |
| North Africa and Middle East | 665 (529 to 804) | 233.01 (185.99 to 283.25) | 1491 (1188 to 1834) | 253.49 (201.48 to 306.9) | 1.24 (1.07 to 1.38) | 0.44 (0.38 to 0.49) |
| Oceania | 19 (16 to 23) | 373 (300.53 to 454.72) | 43 (34 to 52) | 375.93 (300.05 to 458.47) | 1.19 (1.06 to 1.31) | 0.03 (-0.05 to 0.1) |
| South Asia | 3213 (2608 to 3808) | 360.16 (294.62 to 426.58) | 7355 (5959 to 8783) | 422.67 (344.69 to 503.13) | 1.29 (1.2 to 1.37) | 0.81 (0.6 to 1.01) |
| Southeast Asia | 1374 (1096 to 1640) | 341.4 (272.87 to 415.94) | 2432 (1917 to 3011) | 346.69 (275.66 to 422.77) | 0.77 (0.6 to 0.9) | 0.14 (0.06 to 0.23) |
| Southern Latin America | 130 (105 to 157) | 269.3 (217.74 to 324.54) | 189 (151 to 230) | 258.42 (205.53 to 311.27) | 0.46 (0.38 to 0.52) | -0.59 (-0.7 to -0.47) |
| Southern Sub-Saharan Africa | 184 (152 to 221) | 469.65 (383.17 to 562.25) | 382 (312 to 462) | 534.5 (437.81 to 641.57) | 1.07 (0.96 to 1.16) | 0.47 (0.39 to 0.55) |
| Tropical Latin America | 825 (673 to 985) | 653.95 (533.12 to 778.68) | 848 (697 to 1017) | 348.09 (287.44 to 414.65) | 0.03 (-0.04 to 0.1) | -2.53 (-2.77 to -2.29) |
| Western Europe | 1335 (1046 to 1646) | 285.62 (224.79 to 353.55) | 1711 (1345 to 2115) | 285.3 (227.28 to 350.74) | 0.28 (0.24 to 0.32) | 0.03 (-0.01 to 0.07) |
| Western Sub-Saharan Africa | 396 (321 to 474) | 286.72 (233.58 to 341.62) | 1024 (821 to 1238) | 313.5 (252.71 to 379.03) | 1.58 (1.5 to 1.66) | 0.23 (0.17 to 0.3) |

**Supplementary table 4:** **Incidence of gastritis and duodenitis in 1990 and 2019 for all locations, with EAPC from 1990 and 2019.**

| location | Num_1990(x10^3^) | ASR_1990/10^5^ | Num_2019(x10^3^) | ASR_2019/10^5^ | Num_change | EAPC_CI |
| --- | --- | --- | --- | --- | --- | --- |
| Afghanistan | 25.7 (20.5 to 30.4) | 270.68 (215.65 to 325.96) | 87.8 (69.5 to 105.4) | 304.07 (245.44 to 365.81) | 2.42 (2.21 to 2.65) | 0.44 (0.38 to 0.5) |
| Albania | 9.5 (7.7 to 11.5) | 335.7 (269.7 to 404.8) | 12.5 (9.8 to 15.5) | 369.18 (295.05 to 454.4) | 0.31 (0.16 to 0.48) | 0.31 (0.25 to 0.37) |
| Algeria | 40.8 (32.7 to 49.3) | 201.84 (162.47 to 247.28) | 96.3 (76.3 to 118.8) | 230.67 (182.81 to 279.96) | 1.36 (1.11 to 1.59) | 0.44 (0.41 to 0.48) |
| American Samoa | 0.1 (0.1 to 0.2) | 360.7 (285.18 to 441.76) | 0.2 (0.1 to 0.2) | 335.2 (266.48 to 412.52) | 0.33 (0.2 to 0.46) | -0.46 (-0.55 to -0.37) |
| Andorra | 0.2 (0.1 to 0.2) | 260.44 (206.41 to 322.51) | 0.3 (0.3 to 0.4) | 282.58 (223.66 to 346.76) | 1.15 (0.98 to 1.31) | 0.35 (0.33 to 0.38) |
| Angola | 29.4 (24.2 to 34.5) | 493.27 (399.25 to 589.17) | 106.9 (86.1 to 127.1) | 633.51 (511.41 to 764.76) | 2.64 (2.45 to 2.85) | 0.85 (0.82 to 0.88) |
| Antigua and Barbuda | 0.2 (0.2 to 0.3) | 412.34 (334.86 to 485.59) | 0.5 (0.4 to 0.6) | 466.43 (379.65 to 553.63) | 1.19 (1 to 1.37) | 0.42 (0.37 to 0.46) |
| Argentina | 88.8 (71.6 to 107.7) | 272.46 (219.09 to 331.57) | 124.9 (100 to 152.4) | 258.51 (205.9 to 313.21) | 0.41 (0.34 to 0.47) | -0.18 (-0.25 to -0.11) |
| Armenia | 6.1 (4.9 to 7.2) | 188.58 (153.38 to 225.27) | 7.9 (6.3 to 9.6) | 221.3 (180.06 to 265.54) | 0.3 (0.2 to 0.43) | 0.64 (0.6 to 0.68) |
| Australia | 55.8 (44.8 to 69.4) | 304.09 (244.68 to 379.06) | 91.5 (71.7 to 112.7) | 307.82 (243.22 to 377.24) | 0.64 (0.54 to 0.75) | 0.06 (0.05 to 0.08) |
| Austria | 25.3 (19.9 to 31.1) | 266.97 (211.52 to 328.33) | 35.5 (27.8 to 44.3) | 285.82 (227.24 to 356.31) | 0.4 (0.31 to 0.49) | 0.33 (0.3 to 0.36) |
| Azerbaijan | 12.2 (9.7 to 14.7) | 185.65 (147.72 to 225.18) | 23.1 (17.9 to 28.5) | 206.27 (162.37 to 252.28) | 0.89 (0.71 to 1.05) | 0.39 (0.32 to 0.46) |
| Bahamas | 0.8 (0.6 to 0.9) | 375.79 (305.04 to 448.51) | 1.8 (1.4 to 2.2) | 432.58 (352.21 to 520.88) | 1.31 (1.06 to 1.52) | 0.43 (0.38 to 0.47) |
| Bahrain | 0.9 (0.7 to 1.1) | 209.77 (168.76 to 250.8) | 3.9 (3 to 5.1) | 232.81 (185.53 to 282.91) | 3.37 (2.74 to 3.94) | 0.28 (0.2 to 0.37) |
| Bangladesh | 342.2 (280.6 to 402.7) | 462.94 (370.57 to 551.09) | 624 (504.1 to 757.1) | 407.11 (329.2 to 490.73) | 0.82 (0.66 to 1.01) | -0.89 (-1.07 to -0.71) |
| Barbados | 0.8 (0.7 to 1) | 337.14 (272.46 to 406.69) | 1.6 (1.2 to 1.9) | 397.28 (319.35 to 483.73) | 0.84 (0.69 to 1.02) | 0.53 (0.49 to 0.56) |
| Belarus | 28.6 (22.7 to 34.7) | 243.88 (194.38 to 293.49) | 33.2 (25.9 to 41.1) | 270.9 (216.74 to 330.85) | 0.16 (0.07 to 0.24) | 0.5 (0.41 to 0.59) |
| Belgium | 36 (27.6 to 44.7) | 291.3 (224.8 to 362.02) | 48 (37.1 to 59.4) | 313.38 (246.84 to 390.94) | 0.33 (0.27 to 0.4) | 0.22 (0.16 to 0.27) |
| Belize | 0.6 (0.5 to 0.7) | 445.08 (361.44 to 533.38) | 1.8 (1.5 to 2.2) | 506.06 (417.97 to 602.7) | 2.13 (1.91 to 2.36) | 0.44 (0.38 to 0.5) |
| Benin | 8.6 (7 to 10.3) | 271.18 (220.79 to 324.57) | 25.2 (20.4 to 30.5) | 296.07 (238.39 to 361.68) | 1.92 (1.76 to 2.11) | 0.17 (0.1 to 0.24) |
| Bermuda | 0.2 (0.2 to 0.3) | 361.3 (293.72 to 433.35) | 0.4 (0.3 to 0.5) | 438.24 (352.37 to 533.13) | 0.76 (0.58 to 0.93) | 0.66 (0.61 to 0.7) |
| Bhutan | 1.5 (1.3 to 1.8) | 348.39 (284.26 to 414.26) | 2.3 (1.9 to 2.7) | 325.09 (265.25 to 388.4) | 0.48 (0.36 to 0.6) | -0.32 (-0.45 to -0.19) |
| Bolivia (Plurinational State of) | 21.2 (17.2 to 25.2) | 439.97 (359.18 to 523.53) | 50.1 (40.5 to 60.7) | 470.02 (379.67 to 570.15) | 1.37 (1.18 to 1.54) | 0.18 (0.12 to 0.24) |
| Bosnia and Herzegovina | 15.1 (12.1 to 18.2) | 325.14 (263.26 to 389.52) | 15.4 (12 to 19.1) | 347.86 (276.25 to 423.85) | 0.02 (-0.07 to 0.13) | 0.23 (0.15 to 0.32) |
| Botswana | 4.6 (3.8 to 5.6) | 540.53 (438.59 to 654.31) | 10.7 (8.6 to 12.9) | 521.4 (419.22 to 626.11) | 1.3 (1.11 to 1.49) | -0.46 (-0.58 to -0.35) |
| Brazil | 807.7 (658.6 to 963.8) | 656.31 (535.08 to 781.64) | 820.5 (674.3 to 984) | 345.99 (285.72 to 411.92) | 0.02 (-0.06 to 0.08) | -2.57 (-2.82 to -2.32) |
| Brunei Darussalam | 0.3 (0.2 to 0.4) | 128.08 (105.01 to 152.7) | 0.5 (0.4 to 0.6) | 114.92 (92.34 to 137.91) | 0.81 (0.68 to 0.95) | -0.55 (-0.6 to -0.5) |
| Bulgaria | 33.3 (26.6 to 40.1) | 314.93 (255.53 to 377.8) | 38.8 (31 to 47) | 385.48 (312.43 to 458.18) | 0.16 (0.07 to 0.26) | 0.88 (0.79 to 0.96) |
| Burkina Faso | 18.4 (14.9 to 21.8) | 281.81 (227.24 to 336) | 51.1 (41.1 to 61.6) | 328.4 (262.83 to 398.06) | 1.77 (1.62 to 1.92) | 0.5 (0.44 to 0.56) |
| Burundi | 15.4 (12.8 to 18.4) | 455.39 (373.69 to 546.03) | 41.2 (33.4 to 49.4) | 580.94 (463.1 to 703.26) | 1.67 (1.51 to 1.84) | 0.87 (0.78 to 0.96) |
| Cabo Verde | 0.6 (0.5 to 0.7) | 243.43 (194.84 to 302.37) | 1.4 (1.1 to 1.8) | 270.59 (213.83 to 336.97) | 1.37 (1.15 to 1.58) | 0.39 (0.26 to 0.51) |
| Cambodia | 29 (23.6 to 34.4) | 363.9 (297.22 to 434.87) | 53 (42.5 to 63.7) | 348.56 (281.43 to 420.94) | 0.83 (0.63 to 1) | -0.21 (-0.27 to -0.15) |
| Cameroon | 19.8 (16.2 to 23.6) | 275.33 (224.24 to 328.93) | 62.4 (50.3 to 76.9) | 297.99 (240.1 to 364.59) | 2.16 (1.94 to 2.38) | 0.13 (0.05 to 0.21) |
| Canada | 86.9 (70.3 to 106.6) | 283.28 (228.66 to 342.61) | 135.3 (107.1 to 166.4) | 273.77 (219.05 to 336.71) | 0.56 (0.43 to 0.68) | -0.18 (-0.2 to -0.16) |
| Central African Republic | 9.5 (7.7 to 11.3) | 566.01 (454.4 to 684.52) | 21.8 (17.5 to 25.8) | 671.4 (542.21 to 805.21) | 1.29 (1.16 to 1.42) | 0.59 (0.56 to 0.61) |
| Chad | 12.6 (10.3 to 15) | 309.51 (252.11 to 370.59) | 32.8 (26.6 to 39.4) | 323.67 (259.94 to 390.99) | 1.61 (1.47 to 1.77) | 0.01 (-0.03 to 0.06) |
| Chile | 32.2 (26.2 to 38.5) | 266.68 (218.82 to 318.02) | 54.1 (43 to 65.4) | 258.32 (206.22 to 311.73) | 0.68 (0.54 to 0.82) | -1.67 (-2.06 to -1.28) |
| China | 7148.7 (5769.2 to 8656.9) | 705.25 (569.96 to 853.3) | 8989.4 (7302.8 to 10644.3) | 468.59 (387.95 to 552.23) | 0.26 (0.17 to 0.37) | -1.56 (-1.66 to -1.45) |
| Colombia | 57.8 (46.6 to 69.2) | 230.8 (187.01 to 277.85) | 114.8 (92.7 to 137.7) | 224.75 (182.85 to 269.17) | 0.99 (0.83 to 1.15) | -0.16 (-0.23 to -0.08) |
| Comoros | 1.4 (1.2 to 1.7) | 485.5 (394.32 to 581.06) | 3.2 (2.6 to 3.8) | 545.27 (440.3 to 657.83) | 1.24 (1.08 to 1.38) | 0.33 (0.3 to 0.35) |
| Congo | 7 (5.7 to 8.3) | 473.94 (390.25 to 575.46) | 20.4 (16.5 to 24.6) | 549.62 (443.59 to 665.59) | 1.93 (1.72 to 2.15) | 0.55 (0.48 to 0.62) |
| Cook Islands | 0.1 (0 to 0.1) | 321.59 (254.48 to 393.55) | 0.1 (0.1 to 0.1) | 322.93 (255.34 to 396.04) | 0.28 (0.13 to 0.42) | 0 (-0.05 to 0.04) |
| Costa Rica | 5.2 (4.2 to 6.3) | 223.56 (181.07 to 267.92) | 10.2 (8 to 12.3) | 199.92 (160.34 to 241.7) | 0.94 (0.76 to 1.12) | -0.42 (-0.47 to -0.37) |
| Côte d'Ivoire | 22 (17.8 to 26.7) | 267.47 (218.32 to 322.89) | 57.4 (45.6 to 69.6) | 293.02 (235.19 to 353.76) | 1.61 (1.43 to 1.78) | 0.22 (0.16 to 0.28) |
| Croatia | 16.8 (13.1 to 20.7) | 292.11 (233.16 to 354.27) | 17.9 (14.9 to 21.3) | 299.49 (253.73 to 349.79) | 0.06 (-0.04 to 0.19) | -0.29 (-0.46 to -0.11) |
| Cuba | 46.1 (37.9 to 54.2) | 435.78 (356.2 to 515.81) | 74.6 (59.6 to 89.4) | 480.65 (393.58 to 570.97) | 0.62 (0.49 to 0.76) | 0.26 (0.2 to 0.31) |
| Cyprus | 1.6 (1.2 to 1.9) | 191.13 (150.35 to 234.71) | 3.5 (2.7 to 4.4) | 208.37 (163.25 to 260.32) | 1.25 (1.13 to 1.38) | -0.39 (-0.8 to 0.02) |
| Czechia | 32.1 (25.5 to 39.2) | 269.85 (216.03 to 325.12) | 47.4 (37.5 to 59.2) | 328.84 (261.62 to 403.57) | 0.48 (0.39 to 0.56) | 0.99 (0.84 to 1.15) |
| Democratic People's Republic of Korea | 97.3 (77.8 to 116.9) | 506.13 (409.83 to 605.44) | 145.7 (116.1 to 174.7) | 462.25 (375.92 to 550.52) | 0.5 (0.41 to 0.6) | -0.29 (-0.33 to -0.26) |
| Democratic Republic of the Congo | 110.5 (90.3 to 130) | 489.86 (398.71 to 588) | 332 (266 to 396.8) | 641.96 (512.56 to 773.95) | 2.01 (1.83 to 2.19) | 0.91 (0.87 to 0.94) |
| Denmark | 14.8 (11.8 to 18.4) | 232.7 (185.11 to 285.11) | 21.4 (16.8 to 26.3) | 268.16 (212.26 to 329.84) | 0.44 (0.36 to 0.52) | 0.59 (0.52 to 0.66) |
| Djibouti | 1.3 (1 to 1.5) | 466.99 (378.65 to 565.72) | 4.6 (3.7 to 5.6) | 494.08 (397.56 to 600.02) | 2.63 (2.33 to 2.92) | 0.13 (0.06 to 0.21) |
| Dominica | 0.3 (0.2 to 0.3) | 435.95 (354.32 to 520.83) | 0.4 (0.3 to 0.4) | 444.85 (361.5 to 533.11) | 0.24 (0.15 to 0.36) | -0.02 (-0.06 to 0.02) |
| Dominican Republic | 21.3 (17.1 to 25.7) | 381.63 (307.02 to 467.55) | 48.2 (38.9 to 59.4) | 461.8 (370.69 to 565.29) | 1.27 (1.05 to 1.45) | 0.61 (0.57 to 0.65) |
| Ecuador | 35.9 (29.5 to 43.6) | 469.91 (380.09 to 566.49) | 66.9 (58.4 to 75.1) | 399.77 (349.35 to 448.96) | 0.87 (0.64 to 1.15) | -0.72 (-0.91 to -0.53) |
| Egypt | 98 (78.4 to 117.6) | 208.35 (165.62 to 252.43) | 204.4 (162 to 249.3) | 224.07 (177.37 to 273.59) | 1.08 (0.96 to 1.22) | 0.22 (0.16 to 0.27) |
| El Salvador | 9.9 (8.1 to 11.9) | 252 (206.05 to 301.16) | 12.9 (10.4 to 15.5) | 215.56 (174.22 to 260.56) | 0.3 (0.2 to 0.4) | -0.61 (-0.72 to -0.5) |
| Equatorial Guinea | 1.4 (1.1 to 1.6) | 514.68 (408.87 to 621.03) | 4.3 (3.5 to 5.2) | 569.24 (459.42 to 691.07) | 2.19 (1.95 to 2.45) | 0.29 (0.2 to 0.38) |
| Eritrea | 8.1 (6.6 to 9.7) | 467.01 (378.82 to 564.52) | 24.4 (19.9 to 29.2) | 572.54 (464.74 to 691.86) | 2.02 (1.86 to 2.2) | 0.64 (0.6 to 0.68) |
| Estonia | 4.1 (3.3 to 5) | 230.77 (183.25 to 281.27) | 4.5 (3.5 to 5.6) | 268.17 (212.16 to 329.24) | 0.1 (0.03 to 0.18) | 0.71 (0.61 to 0.81) |
| Eswatini | 2.3 (1.9 to 2.8) | 469.17 (379.95 to 567.94) | 4.6 (3.7 to 5.6) | 518.54 (423.63 to 622) | 0.97 (0.84 to 1.1) | 0.22 (0.12 to 0.32) |
| Ethiopia | 161.4 (132.8 to 190.6) | 506.59 (413.82 to 606.43) | 349.7 (284.7 to 416.7) | 528.52 (426.04 to 637.52) | 1.17 (1.09 to 1.24) | 0.04 (0.01 to 0.08) |
| Fiji | 2.2 (1.8 to 2.7) | 351.75 (283.23 to 426.01) | 3 (2.4 to 3.7) | 327.41 (259.82 to 399.89) | 0.35 (0.22 to 0.5) | -0.32 (-0.39 to -0.24) |
| Finland | 14.5 (11.5 to 17.9) | 234.82 (187.18 to 286.48) | 19.1 (14.9 to 23.6) | 244.99 (193.42 to 300.65) | 0.31 (0.23 to 0.41) | -0.04 (-0.39 to 0.31) |
| France | 177.3 (136.7 to 220) | 262.79 (203.69 to 327.39) | 248.3 (191.5 to 314.3) | 283.27 (221.23 to 358.42) | 0.4 (0.32 to 0.48) | 0.34 (0.32 to 0.37) |
| Gabon | 3 (2.5 to 3.5) | 444.47 (362.61 to 533.39) | 6.6 (5.3 to 7.9) | 492.62 (399.71 to 597.41) | 1.21 (1.06 to 1.39) | 0.29 (0.26 to 0.32) |
| Gambia | 1.9 (1.6 to 2.3) | 296.94 (240.31 to 356.84) | 4.6 (3.7 to 5.7) | 292.34 (232.36 to 358.5) | 1.42 (1.26 to 1.59) | -0.26 (-0.32 to -0.19) |
| Georgia | 10.7 (8.4 to 12.9) | 181.06 (144.2 to 218.58) | 9.3 (7.4 to 11.3) | 210.41 (168.58 to 255.69) | -0.13 (-0.18 to -0.07) | 0.61 (0.56 to 0.66) |
| Germany | 272.3 (210 to 334.9) | 265.84 (209.16 to 329.64) | 364.2 (285.9 to 446.1) | 294.58 (233.04 to 361.28) | 0.34 (0.26 to 0.42) | 0.46 (0.42 to 0.5) |
| Ghana | 26.9 (21.8 to 32.3) | 250.06 (201.67 to 301.63) | 82.9 (66.1 to 101.6) | 324.22 (258.36 to 400.74) | 2.08 (1.9 to 2.27) | 0.93 (0.84 to 1.02) |
| Greece | 33.6 (25.8 to 42) | 262.71 (205.33 to 327.49) | 41.4 (32 to 52) | 282.35 (219.82 to 354.67) | 0.23 (0.17 to 0.3) | 0.33 (0.3 to 0.35) |
| Greenland | 0.1 (0.1 to 0.2) | 296.25 (239.07 to 355.1) | 0.2 (0.1 to 0.2) | 256.52 (207.36 to 312.81) | 0.14 (0.03 to 0.28) | -0.85 (-0.98 to -0.72) |
| Grenada | 0.3 (0.3 to 0.4) | 459.76 (375.43 to 548.94) | 0.6 (0.4 to 0.7) | 486.39 (396.53 to 579.05) | 0.76 (0.59 to 0.92) | 0.07 (0.04 to 0.11) |
| Guam | 0.4 (0.3 to 0.5) | 321.77 (255.84 to 394) | 0.6 (0.5 to 0.7) | 324.28 (255.38 to 400.91) | 0.54 (0.39 to 0.71) | -0.04 (-0.08 to 0.01) |
| Guatemala | 16.1 (13 to 19) | 286.23 (233.71 to 338.65) | 40.8 (33.4 to 48.3) | 296.22 (241 to 354.08) | 1.54 (1.36 to 1.72) | 0.16 (0.01 to 0.3) |
| Guinea | 12.7 (10.4 to 14.9) | 278.76 (228.48 to 330.59) | 26.4 (21.3 to 31.6) | 301.12 (243.53 to 365.6) | 1.08 (0.96 to 1.22) | 0.11 (0.04 to 0.18) |
| Guinea-Bissau | 2.1 (1.7 to 2.5) | 307.28 (248.87 to 370.89) | 4.3 (3.4 to 5.2) | 318.32 (255.62 to 384.9) | 1.05 (0.93 to 1.18) | -0.02 (-0.06 to 0.03) |
| Guyana | 2.5 (2 to 2.9) | 428.66 (351.54 to 505.95) | 3.7 (3 to 4.3) | 495.47 (405.45 to 584.99) | 0.49 (0.38 to 0.63) | 0.46 (0.44 to 0.49) |
| Haiti | 22.6 (18.7 to 26.4) | 472 (387.45 to 559.52) | 53.3 (43.8 to 64.1) | 535.65 (439.39 to 647.58) | 1.36 (1.22 to 1.5) | 0.4 (0.36 to 0.44) |
| Honduras | 9.9 (8 to 11.6) | 327.79 (266.67 to 391.98) | 22.8 (18.5 to 27.3) | 302.61 (243.39 to 364.76) | 1.31 (1.15 to 1.47) | -0.42 (-0.53 to -0.31) |
| Hungary | 38.1 (30.7 to 46.3) | 308.8 (249.74 to 370.45) | 47.5 (38.1 to 57.8) | 357.05 (290.27 to 427.41) | 0.24 (0.17 to 0.33) | 0.49 (0.45 to 0.54) |
| Iceland | 0.6 (0.4 to 0.7) | 214.84 (168.97 to 263.09) | 1 (0.8 to 1.3) | 242.68 (192.53 to 299.03) | 0.87 (0.77 to 0.97) | 0.41 (0.39 to 0.44) |
| India | 2490.7 (2013.9 to 2955.6) | 348.33 (284.92 to 413.39) | 5906.7 (4775.2 to 7053.8) | 430.09 (351.88 to 510.67) | 1.37 (1.29 to 1.45) | 1.1 (0.85 to 1.34) |
| Indonesia | 525 (416.7 to 633.6) | 320.54 (257.03 to 391.49) | 905.3 (710.2 to 1128.8) | 333.54 (265.83 to 409.78) | 0.72 (0.55 to 0.85) | 0.28 (0.15 to 0.42) |
| Iran (Islamic Republic of) | 136.5 (108.7 to 164.3) | 285.39 (228.61 to 346.11) | 274.1 (216.9 to 340.5) | 309.2 (247.76 to 374.38) | 1.01 (0.77 to 1.21) | 0.88 (0.66 to 1.11) |
| Iraq | 29.5 (23.3 to 35.9) | 215.99 (172.28 to 264.78) | 88.2 (70 to 108.2) | 239.24 (190.2 to 294.05) | 1.99 (1.76 to 2.19) | 0.43 (0.36 to 0.5) |
| Ireland | 9.8 (7.8 to 12.2) | 267.29 (211.24 to 331.51) | 17.9 (13.8 to 22.6) | 290 (226.42 to 361.64) | 0.82 (0.71 to 0.91) | 0.33 (0.28 to 0.38) |
| Israel | 12.2 (9.7 to 14.9) | 261.12 (206.61 to 320.02) | 27.6 (21.8 to 34.7) | 280.26 (222.39 to 352.51) | 1.27 (1.14 to 1.4) | 0.34 (0.31 to 0.38) |
| Italy | 245.8 (193.1 to 303.8) | 345.91 (274.21 to 425.29) | 189 (154.1 to 228.1) | 221.43 (182.88 to 264.34) | -0.23 (-0.28 to -0.16) | -1.83 (-1.98 to -1.69) |
| Jamaica | 7.4 (6.1 to 8.9) | 384.06 (310.42 to 462.65) | 12.9 (10.4 to 15.7) | 439.44 (355.08 to 529.73) | 0.74 (0.6 to 0.87) | 0.51 (0.47 to 0.55) |
| Japan | 175.7 (140.1 to 214.8) | 128.88 (102.98 to 156.73) | 153.8 (126.6 to 185.2) | 106.93 (87.86 to 128.01) | -0.12 (-0.19 to -0.05) | -1.01 (-1.18 to -0.83) |
| Jordan | 4.7 (3.7 to 5.7) | 163.92 (129.18 to 199.21) | 21.8 (17.6 to 26.5) | 207.91 (168.91 to 251.52) | 3.64 (3.25 to 4) | 0.99 (0.9 to 1.08) |
| Kazakhstan | 30.4 (24.4 to 36.1) | 199.89 (161.09 to 237.91) | 40.9 (32.5 to 49.6) | 214.26 (171.12 to 259.11) | 0.35 (0.26 to 0.43) | 0.29 (0.22 to 0.35) |
| Kenya | 71.2 (58.6 to 84) | 549.24 (448.91 to 659.45) | 190.6 (155.9 to 226.1) | 561.43 (458.51 to 676.46) | 1.68 (1.57 to 1.76) | 0.28 (0.11 to 0.44) |
| Kiribati | 0.2 (0.2 to 0.3) | 368.78 (299.4 to 444.07) | 0.4 (0.3 to 0.5) | 360.08 (288.93 to 442.24) | 0.67 (0.56 to 0.79) | -0.13 (-0.21 to -0.06) |
| Kuwait | 3 (2.3 to 3.8) | 193.15 (154.7 to 236.1) | 10.8 (8.4 to 14.2) | 227.35 (180.98 to 278.13) | 2.61 (2.26 to 2.92) | 0.51 (0.49 to 0.54) |
| Kyrgyzstan | 7.1 (5.7 to 8.4) | 186.5 (148.56 to 223.69) | 13 (10.3 to 15.9) | 214.24 (170.81 to 261.77) | 0.83 (0.71 to 0.94) | 0.53 (0.46 to 0.6) |
| Lao People's Democratic Republic | 12.3 (10 to 14.7) | 363.89 (298.56 to 434.58) | 22.5 (18.1 to 27.1) | 355.94 (288.29 to 430.25) | 0.84 (0.68 to 0.98) | -0.08 (-0.15 to -0.02) |
| Latvia | 7.8 (6.1 to 9.4) | 251.29 (200.81 to 303.57) | 6.9 (5.5 to 8.5) | 274.43 (222.06 to 333.63) | -0.11 (-0.16 to -0.05) | 0.39 (0.33 to 0.45) |
| Lebanon | 6.4 (5.1 to 7.8) | 220.04 (173.98 to 270.98) | 13.4 (10.7 to 16.5) | 254.68 (201.66 to 310.08) | 1.09 (0.94 to 1.23) | 0.6 (0.49 to 0.71) |
| Lesotho | 6.7 (5.5 to 8) | 517.91 (420.54 to 621.41) | 10.2 (8.3 to 12.5) | 574.3 (469.4 to 700.82) | 0.53 (0.43 to 0.62) | 0.18 (0.08 to 0.28) |
| Liberia | 3.7 (3 to 4.3) | 248.28 (199.36 to 295.64) | 11.4 (9.1 to 14.2) | 309.19 (245.46 to 382.15) | 2.1 (1.87 to 2.38) | 0.7 (0.63 to 0.77) |
| Libya | 6.6 (5.2 to 8) | 197.76 (157.97 to 243.03) | 16.3 (12.8 to 20.7) | 233.67 (184.66 to 287.4) | 1.48 (1.16 to 1.76) | 0.62 (0.58 to 0.65) |
| Lithuania | 9.5 (7.5 to 11.5) | 232.59 (184.92 to 283.06) | 10.3 (8 to 12.5) | 277.09 (221.31 to 335.43) | 0.09 (0.01 to 0.17) | 0.84 (0.75 to 0.93) |
| Luxembourg | 1.7 (1.4 to 2.2) | 371.8 (293.18 to 457.14) | 3.2 (2.5 to 4) | 394.29 (308.71 to 495.74) | 0.83 (0.73 to 0.92) | -0.2 (-0.38 to -0.02) |
| Madagascar | 31 (25.5 to 36.7) | 415.5 (341.22 to 498.24) | 92.4 (74.7 to 110.6) | 537.14 (433.97 to 651.78) | 1.98 (1.8 to 2.18) | 0.85 (0.78 to 0.91) |
| Malawi | 26.9 (22.2 to 31.7) | 470.55 (380.76 to 562.44) | 64.9 (52.9 to 78.1) | 595.31 (475.73 to 725.71) | 1.41 (1.29 to 1.56) | 0.78 (0.74 to 0.82) |
| Malaysia | 41.3 (32.7 to 50.5) | 275.87 (220.11 to 339.6) | 90.8 (71.4 to 111.6) | 290.68 (230.24 to 355.08) | 1.2 (1 to 1.38) | 0.23 (0.17 to 0.29) |
| Maldives | 0.6 (0.5 to 0.8) | 372.6 (293.36 to 461.53) | 1.8 (1.4 to 2.2) | 363.34 (287.21 to 445.05) | 1.74 (1.36 to 2.13) | 0.03 (-0.01 to 0.07) |
| Mali | 17.1 (14 to 20.2) | 276.54 (225.37 to 333.37) | 45.5 (36.9 to 54.5) | 318.15 (255.06 to 386.29) | 1.66 (1.52 to 1.8) | 0.44 (0.39 to 0.5) |
| Malta | 1.6 (1.3 to 2) | 384.89 (304.38 to 479.16) | 2.6 (2 to 3.2) | 415.09 (327.84 to 516.46) | 0.6 (0.46 to 0.73) | 0.55 (0.47 to 0.64) |
| Marshall Islands | 0.1 (0.1 to 0.2) | 372.13 (301.57 to 454.22) | 0.2 (0.1 to 0.2) | 341.74 (273.13 to 414.5) | 0.45 (0.28 to 0.6) | -0.31 (-0.41 to -0.21) |
| Mauritania | 4 (3.3 to 4.8) | 276.43 (224.83 to 332.63) | 8.8 (7.1 to 10.8) | 293.36 (234.47 to 362.74) | 1.19 (1.05 to 1.36) | 0.04 (-0.03 to 0.11) |
| Mauritius | 2.8 (2.3 to 3.4) | 287.22 (233.25 to 345.86) | 4.5 (3.6 to 5.6) | 289.84 (231.72 to 353.12) | 0.61 (0.44 to 0.78) | 0.01 (-0.05 to 0.06) |
| Mexico | 186.8 (152.1 to 221.3) | 313.01 (254.1 to 376.19) | 237.4 (199.2 to 278.9) | 190.95 (160.45 to 223.52) | 0.27 (0.19 to 0.36) | -1.9 (-2.15 to -1.64) |
| Micronesia (Federated States of) | 0.3 (0.2 to 0.3) | 364.63 (293.95 to 446.33) | 0.3 (0.3 to 0.4) | 345.92 (276.27 to 420.14) | 0.15 (0.03 to 0.27) | -0.23 (-0.3 to -0.16) |
| Monaco | 0.1 (0.1 to 0.1) | 264.85 (210 to 331.19) | 0.2 (0.1 to 0.2) | 283.01 (223.38 to 352.89) | 0.36 (0.28 to 0.45) | 0.31 (0.28 to 0.34) |
| Mongolia | 3.4 (2.7 to 4.1) | 206.47 (167.48 to 249.64) | 8.2 (6.5 to 9.9) | 258.28 (209.66 to 308.6) | 1.4 (1.14 to 1.63) | 0.99 (0.92 to 1.07) |
| Montenegro | 2.1 (1.7 to 2.6) | 327.61 (264.4 to 399.62) | 2.9 (2.2 to 3.6) | 364.19 (292.95 to 445.07) | 0.36 (0.27 to 0.44) | 0.34 (0.28 to 0.41) |
| Morocco | 52.3 (41.5 to 63.4) | 243.68 (192.65 to 298.7) | 90.7 (71.8 to 110.4) | 246.02 (196.08 to 298.44) | 0.73 (0.58 to 0.89) | 0.05 (0 to 0.1) |
| Mozambique | 38.8 (31.6 to 46.6) | 459.34 (370.56 to 557.09) | 97.1 (79.4 to 116.1) | 576.48 (464.53 to 696.85) | 1.5 (1.36 to 1.64) | 0.74 (0.67 to 0.81) |
| Myanmar | 126.5 (102.1 to 150.5) | 362.61 (294.24 to 433.27) | 198.6 (157.6 to 247.2) | 362.65 (289.74 to 446.62) | 0.57 (0.4 to 0.73) | 0 (-0.08 to 0.09) |
| Namibia | 4.6 (3.8 to 5.5) | 478.65 (383.25 to 578.41) | 10.1 (8.2 to 12.3) | 528.89 (427.54 to 637.91) | 1.18 (1.03 to 1.32) | 0.2 (0.11 to 0.29) |
| Nauru | 0 (0 to 0) | 340.94 (271.4 to 417.37) | 0 (0 to 0) | 340 (269.16 to 418.43) | 0.04 (-0.02 to 0.1) | -0.02 (-0.08 to 0.04) |
| Nepal | 54.6 (44.8 to 64) | 367.7 (302.78 to 433.14) | 108.6 (88.5 to 129.8) | 395.52 (322.61 to 474.95) | 0.99 (0.87 to 1.12) | 0.19 (0.12 to 0.25) |
| Netherlands | 46.5 (36.2 to 57.9) | 266.21 (206.38 to 329.91) | 66.9 (52.2 to 83.5) | 283.26 (224.08 to 350.43) | 0.44 (0.35 to 0.54) | 0.26 (0.24 to 0.28) |
| New Zealand | 13.4 (10.9 to 16.6) | 370.37 (298.98 to 454.74) | 18.7 (15.4 to 21.9) | 357.47 (297.54 to 425.88) | 0.39 (0.28 to 0.52) | -0.01 (-0.24 to 0.22) |
| Nicaragua | 7.2 (5.8 to 8.6) | 285.17 (232.86 to 340.72) | 13.3 (10.8 to 16) | 238.36 (193.31 to 282.91) | 0.86 (0.68 to 1.03) | -0.64 (-0.69 to -0.59) |
| Niger | 15.7 (12.8 to 18.9) | 300.09 (245.71 to 364.41) | 46.6 (38.3 to 55.5) | 335.48 (271.72 to 404.74) | 1.96 (1.8 to 2.13) | 0.3 (0.25 to 0.35) |
| Nigeria | 202.6 (163 to 242.1) | 300.14 (243.99 to 360.01) | 492.7 (393.1 to 595) | 316.28 (254.09 to 384.49) | 1.43 (1.36 to 1.51) | 0.11 (0.04 to 0.18) |
| Niue | 0 (0 to 0) | 340.43 (269.35 to 414.63) | 0 (0 to 0) | 328.95 (259.87 to 403.54) | -0.13 (-0.2 to -0.06) | -0.17 (-0.23 to -0.11) |
| North Macedonia | 6.3 (5 to 7.7) | 308.26 (246.66 to 378.91) | 9.4 (7.4 to 11.6) | 345.02 (279.11 to 414.31) | 0.49 (0.39 to 0.61) | 0.39 (0.32 to 0.46) |
| Northern Mariana Islands | 0.1 (0.1 to 0.2) | 305.95 (242.98 to 374.56) | 0.2 (0.1 to 0.2) | 326.64 (258.59 to 404.47) | 0.5 (0.26 to 0.82) | 0.18 (0.15 to 0.22) |
| Norway | 19.4 (15.5 to 23.7) | 387.84 (308.97 to 476.11) | 26.6 (20.9 to 32.8) | 388.01 (308.29 to 478.32) | 0.37 (0.32 to 0.43) | -0.46 (-0.61 to -0.3) |
| Oman | 3.7 (2.9 to 4.6) | 235.77 (186.98 to 290.35) | 10 (7.6 to 12.8) | 232.64 (184.68 to 285.15) | 1.71 (1.46 to 1.96) | -0.18 (-0.24 to -0.11) |
| Pakistan | 323.9 (264.7 to 384.4) | 374.54 (306.1 to 442.44) | 713.4 (571.9 to 853.7) | 389.06 (317.61 to 462.34) | 1.2 (1.11 to 1.29) | 0.16 (0.02 to 0.31) |
| Palau | 0 (0 to 0.1) | 375.66 (299.52 to 458.63) | 0.1 (0.1 to 0.1) | 368.07 (297.86 to 447.15) | 0.75 (0.55 to 0.95) | -0.11 (-0.12 to -0.09) |
| Palestine | 3.7 (2.9 to 4.4) | 240.51 (189.55 to 292.68) | 10.7 (8.5 to 12.9) | 259.68 (207.28 to 316.69) | 1.91 (1.7 to 2.09) | 0.33 (0.26 to 0.41) |
| Panama | 4.6 (3.7 to 5.5) | 236.7 (188.95 to 283.92) | 8.7 (7 to 10.6) | 208.86 (167.29 to 254.93) | 0.91 (0.75 to 1.07) | -0.48 (-0.54 to -0.42) |
| Papua New Guinea | 12.6 (10.1 to 15.3) | 382.85 (306.74 to 467.95) | 31.9 (25.1 to 38.8) | 386.81 (306.69 to 472.95) | 1.52 (1.37 to 1.67) | 0.06 (-0.02 to 0.13) |
| Paraguay | 17.5 (13.9 to 21.2) | 563.36 (448.16 to 687.15) | 27.7 (22.1 to 33.7) | 425.16 (339.18 to 516.74) | 0.58 (0.48 to 0.68) | -1.01 (-1.18 to -0.84) |
| Peru | 77.6 (63.5 to 93.7) | 461.31 (376.86 to 555.01) | 184.6 (150 to 223.4) | 547.79 (444.97 to 660.04) | 1.38 (1.17 to 1.57) | 0.75 (0.62 to 0.87) |
| Philippines | 205.6 (164.1 to 248.8) | 383.75 (311.66 to 460.5) | 384 (309.1 to 465) | 366.27 (296.04 to 441.32) | 0.87 (0.74 to 0.98) | -0.1 (-0.19 to -0.01) |
| Poland | 267.1 (214 to 323.1) | 656.16 (528.19 to 789.39) | 322.6 (269.2 to 381.4) | 623.56 (530.08 to 722.52) | 0.21 (0.11 to 0.34) | -0.01 (-0.14 to 0.12) |
| Portugal | 32.7 (25.6 to 40.7) | 273.36 (217 to 340.49) | 43.7 (34 to 54.7) | 284.74 (224.75 to 355.27) | 0.34 (0.25 to 0.43) | 0.21 (0.16 to 0.25) |
| Puerto Rico | 12.9 (10.3 to 15.7) | 358.43 (286.17 to 435.45) | 20.5 (16.1 to 25.5) | 431.03 (343.03 to 527.95) | 0.6 (0.45 to 0.76) | 0.63 (0.59 to 0.67) |
| Qatar | 0.8 (0.6 to 1) | 194.99 (155.56 to 237.63) | 6.5 (5 to 8.4) | 231.69 (185.85 to 279.02) | 7.26 (6.59 to 7.89) | 0.71 (0.67 to 0.76) |
| Republic of Korea | 62.4 (50.7 to 74.9) | 156.9 (129.96 to 184.14) | 70.1 (56 to 85.7) | 115.04 (93.35 to 139.8) | 0.12 (0 to 0.24) | -1.64 (-1.82 to -1.46) |
| Republic of Moldova | 11.8 (9.4 to 14.4) | 256.48 (204.8 to 312.76) | 13.4 (10.3 to 16.6) | 285.99 (227.91 to 350.56) | 0.14 (0.06 to 0.22) | 0.51 (0.42 to 0.6) |
| Romania | 88 (70.9 to 105.6) | 338.51 (272.28 to 406.79) | 98.1 (79.5 to 119.4) | 374.77 (308.61 to 447.79) | 0.12 (0.04 to 0.2) | 0.41 (0.35 to 0.48) |
| Russian Federation | 445.6 (355.6 to 539.6) | 266.48 (214.23 to 321.31) | 551.3 (436.6 to 671.5) | 302.94 (243.84 to 365.17) | 0.24 (0.19 to 0.29) | 0.64 (0.56 to 0.73) |
| Rwanda | 21.6 (18 to 25.5) | 503.63 (412.83 to 601.4) | 50 (40.9 to 59.8) | 588.17 (481.75 to 708.79) | 1.32 (1.16 to 1.49) | 0.52 (0.48 to 0.55) |
| Saint Kitts and Nevis | 0.2 (0.1 to 0.2) | 457.57 (372.69 to 547.09) | 0.4 (0.3 to 0.4) | 500.51 (404.56 to 601.45) | 1.26 (1.04 to 1.52) | 0.21 (0.17 to 0.25) |
| Saint Lucia | 0.4 (0.3 to 0.5) | 384.23 (311.98 to 456.93) | 0.9 (0.7 to 1.1) | 434.07 (351.9 to 520.27) | 1.18 (0.91 to 1.41) | 0.43 (0.41 to 0.46) |
| Saint Vincent and the Grenadines | 0.4 (0.4 to 0.5) | 522.78 (428.68 to 614.66) | 0.7 (0.6 to 0.9) | 562.49 (460.22 to 662.87) | 0.71 (0.53 to 0.89) | 0.12 (0.04 to 0.21) |
| Samoa | 0.4 (0.3 to 0.5) | 322.53 (259.2 to 393.03) | 0.6 (0.5 to 0.7) | 316.84 (251.15 to 388.39) | 0.43 (0.32 to 0.52) | -0.15 (-0.2 to -0.1) |
| San Marino | 0.1 (0.1 to 0.1) | 262.77 (205.72 to 326.85) | 0.1 (0.1 to 0.2) | 284.3 (223.52 to 356.05) | 0.72 (0.63 to 0.83) | 0.35 (0.33 to 0.38) |
| Sao Tome and Principe | 0.2 (0.2 to 0.2) | 246.01 (197.13 to 295.77) | 0.4 (0.3 to 0.5) | 261.63 (207.19 to 327.55) | 1.08 (0.9 to 1.28) | 0.12 (0.05 to 0.19) |
| Saudi Arabia | 28.4 (22.1 to 35.1) | 220.66 (175.04 to 270.3) | 81.6 (63.2 to 103.8) | 223.65 (178.2 to 271.05) | 1.87 (1.53 to 2.15) | 0.1 (0.04 to 0.16) |
| Senegal | 13.8 (11.4 to 16.5) | 269.4 (219.25 to 321.82) | 31.2 (25.3 to 37.8) | 277.98 (222.71 to 336.98) | 1.26 (1.13 to 1.41) | -0.03 (-0.12 to 0.05) |
| Serbia | 25.7 (20.3 to 31.1) | 239.61 (192.68 to 287.19) | 36.8 (29.7 to 44.8) | 312.2 (253.77 to 373.22) | 0.43 (0.33 to 0.55) | 1.53 (1.33 to 1.72) |
| Seychelles | 0.2 (0.2 to 0.3) | 349.34 (283.35 to 416.69) | 0.4 (0.3 to 0.5) | 346.03 (277.26 to 420.38) | 0.85 (0.65 to 1.01) | -0.09 (-0.13 to -0.06) |
| Sierra Leone | 7.1 (5.7 to 8.5) | 258.05 (207.59 to 311.3) | 19.1 (15.3 to 23.2) | 315.91 (254.46 to 384.79) | 1.71 (1.56 to 1.88) | 0.72 (0.63 to 0.8) |
| Singapore | 3.5 (2.8 to 4.4) | 112.41 (90.07 to 136.15) | 7.1 (5.6 to 8.9) | 111.85 (90.33 to 136.41) | 1.01 (0.79 to 1.2) | -0.03 (-0.04 to -0.02) |
| Slovakia | 17.4 (14.1 to 21.2) | 309.66 (252.06 to 373.55) | 25.8 (20.6 to 31.7) | 360.34 (292.65 to 432.63) | 0.48 (0.39 to 0.58) | 0.57 (0.53 to 0.6) |
| Slovenia | 7.5 (6.1 to 9.2) | 335.88 (273.01 to 408.15) | 10.7 (8.4 to 13.2) | 373.14 (302 to 453.93) | 0.42 (0.33 to 0.52) | 0.59 (0.33 to 0.85) |
| Solomon Islands | 0.9 (0.7 to 1.1) | 361.9 (292.42 to 443.64) | 1.9 (1.5 to 2.3) | 350.51 (280.31 to 425.02) | 1 (0.84 to 1.13) | -0.13 (-0.2 to -0.06) |
| Somalia | 19.6 (15.9 to 23.3) | 463.25 (377.87 to 553.36) | 60.8 (49.8 to 72.4) | 537.11 (433.12 to 647.25) | 2.1 (1.94 to 2.26) | 0.47 (0.43 to 0.51) |
| South Africa | 137.7 (113.3 to 165.3) | 471.67 (385.35 to 564.67) | 290.5 (236 to 351.5) | 536.72 (438.98 to 643.53) | 1.11 (0.98 to 1.21) | 0.51 (0.36 to 0.66) |
| South Sudan | 15.8 (13 to 18.6) | 445.36 (360.75 to 532.65) | 30.5 (24.8 to 36.8) | 528.24 (424.38 to 641.88) | 0.94 (0.82 to 1.06) | 0.58 (0.52 to 0.63) |
| Spain | 119.6 (92.3 to 149.5) | 262.63 (205.76 to 327.76) | 185.3 (142.7 to 234.4) | 284.1 (222.48 to 355.72) | 0.55 (0.47 to 0.64) | 0.34 (0.31 to 0.36) |
| Sri Lanka | 58 (46.5 to 69.8) | 383.26 (306.62 to 462.95) | 86.3 (69.2 to 104.2) | 352.78 (284.57 to 424.03) | 0.49 (0.35 to 0.66) | -0.29 (-0.36 to -0.21) |
| Sudan | 38.4 (30.6 to 46.4) | 241.3 (192.68 to 294.04) | 94.1 (74.9 to 113.8) | 282.55 (225.86 to 343.5) | 1.45 (1.31 to 1.58) | 0.59 (0.54 to 0.64) |
| Suriname | 1.3 (1 to 1.5) | 385.49 (314.05 to 457.21) | 2.7 (2.2 to 3.3) | 437.6 (355.52 to 522.73) | 1.15 (0.97 to 1.32) | 0.39 (0.36 to 0.42) |
| Sweden | 29.8 (23.8 to 36.5) | 276.23 (220.25 to 338.08) | 27.4 (21.8 to 33.4) | 210.52 (167.48 to 257.87) | -0.08 (-0.13 to -0.03) | -1.26 (-1.41 to -1.12) |
| Switzerland | 19.9 (15.6 to 24.6) | 234.74 (185.08 to 290.67) | 30.6 (23.8 to 37.8) | 250.11 (197.37 to 309.89) | 0.54 (0.46 to 0.63) | 0.19 (-0.11 to 0.49) |
| Syrian Arab Republic | 20.8 (16.5 to 25) | 211.61 (168.31 to 257.54) | 34.5 (27.3 to 42.4) | 235.69 (187.13 to 288.19) | 0.66 (0.48 to 0.86) | 0.4 (0.36 to 0.44) |
| Taiwan (Province of China) | 85.7 (68.7 to 104.1) | 465.65 (372.3 to 563.82) | 153.5 (115.3 to 192.6) | 441.82 (343.22 to 552.87) | 0.79 (0.6 to 1.01) | -0.16 (-0.24 to -0.08) |
| Tajikistan | 8.1 (6.6 to 9.8) | 194.11 (156.91 to 234.39) | 18.6 (14.9 to 22.5) | 221.1 (178.27 to 268.89) | 1.28 (1.13 to 1.44) | 0.51 (0.46 to 0.57) |
| Thailand | 186.4 (146.7 to 228) | 360.89 (284.51 to 442.57) | 330 (257.4 to 410.5) | 369.93 (293.26 to 449.9) | 0.77 (0.57 to 0.97) | 0.19 (0.15 to 0.24) |
| Timor-Leste | 2.2 (1.8 to 2.7) | 360.62 (290.68 to 431.33) | 3.8 (3.1 to 4.5) | 352.08 (285.19 to 425.01) | 0.71 (0.59 to 0.82) | -0.07 (-0.16 to 0.02) |
| Togo | 6.7 (5.5 to 8) | 284.85 (230.46 to 339.79) | 19.9 (16.2 to 24) | 328.23 (266.73 to 394.67) | 1.97 (1.8 to 2.17) | 0.46 (0.38 to 0.54) |
| Tokelau | 0 (0 to 0) | 355.8 (282.66 to 438.61) | 0 (0 to 0) | 329.44 (261.89 to 405.24) | -0.1 (-0.17 to -0.04) | -0.3 (-0.37 to -0.22) |
| Tonga | 0.3 (0.2 to 0.3) | 369.86 (297.2 to 445.51) | 0.3 (0.2 to 0.4) | 341.56 (274.41 to 415.89) | 0.09 (0.02 to 0.17) | -0.36 (-0.43 to -0.29) |
| Trinidad and Tobago | 5.8 (4.8 to 6.9) | 581.91 (477.74 to 695.95) | 9.8 (7.9 to 11.9) | 567.18 (462.57 to 681) | 0.69 (0.55 to 0.87) | -0.27 (-0.47 to -0.07) |
| Tunisia | 16 (12.8 to 19.3) | 220.97 (176.45 to 269.2) | 30.9 (24.5 to 38.5) | 242.64 (193.11 to 296.7) | 0.93 (0.74 to 1.1) | 0.37 (0.33 to 0.41) |
| Turkey | 118.7 (94.6 to 142.9) | 226.13 (180.1 to 276.89) | 219.8 (173.6 to 273.1) | 244.81 (194.16 to 298.73) | 0.85 (0.67 to 1.02) | 0.34 (0.27 to 0.42) |
| Turkmenistan | 5.6 (4.5 to 6.8) | 188.02 (150.46 to 230.67) | 10.5 (8.3 to 12.8) | 212.86 (168.31 to 258.08) | 0.88 (0.72 to 1.03) | 0.56 (0.48 to 0.64) |
| Tuvalu | 0 (0 to 0) | 365.25 (291.69 to 449.12) | 0 (0 to 0) | 342.05 (273.22 to 414.91) | 0.25 (0.18 to 0.34) | -0.25 (-0.32 to -0.19) |
| Uganda | 45.5 (37.9 to 53.6) | 472.3 (384.53 to 565.84) | 121.7 (99.7 to 144.4) | 546.54 (443.66 to 664.27) | 1.68 (1.53 to 1.84) | 0.43 (0.38 to 0.47) |
| Ukraine | 166.8 (132.6 to 201.6) | 274.68 (220.89 to 331.29) | 173.7 (135.9 to 212.9) | 311.57 (247.69 to 376.92) | 0.04 (-0.01 to 0.09) | 0.59 (0.5 to 0.68) |
| United Arab Emirates | 3.3 (2.5 to 4.1) | 195.91 (154.71 to 236.89) | 23.9 (17.2 to 33) | 217.35 (170.58 to 265.8) | 6.29 (5.28 to 7.33) | 0.36 (0.3 to 0.41) |
| United Kingdom | 218.8 (172.6 to 270.4) | 317.17 (250.34 to 393.15) | 305.8 (245.5 to 372.5) | 348.09 (279.86 to 423.48) | 0.4 (0.34 to 0.46) | 0.63 (0.44 to 0.82) |
| United Republic of Tanzania | 62.8 (51.8 to 74.1) | 399.72 (327.67 to 478.87) | 172.4 (141 to 207.5) | 478.75 (386.59 to 577.19) | 1.75 (1.59 to 1.9) | 0.54 (0.48 to 0.6) |
| United States of America | 1236.5 (987.6 to 1532.5) | 437.17 (347.13 to 539.17) | 1155.1 (971.1 to 1353.4) | 275.41 (235.22 to 319.39) | -0.07 (-0.16 to 0.05) | -1.73 (-1.88 to -1.57) |
| United States Virgin Islands | 0.4 (0.3 to 0.5) | 357.16 (287.28 to 433.3) | 0.6 (0.5 to 0.7) | 422.19 (339.7 to 509.29) | 0.59 (0.44 to 0.77) | 0.6 (0.56 to 0.63) |
| Uruguay | 8.6 (7 to 10.2) | 256.76 (208.52 to 306.98) | 10.1 (8.1 to 12.4) | 256.66 (204.76 to 314.16) | 0.18 (0.11 to 0.25) | -0.07 (-0.11 to -0.04) |
| Uzbekistan | 30.3 (24.1 to 36.6) | 180.96 (143.95 to 221.81) | 66.6 (52.9 to 81.6) | 210.17 (167.89 to 257.74) | 1.2 (1.01 to 1.36) | 0.67 (0.58 to 0.76) |
| Vanuatu | 0.5 (0.4 to 0.6) | 391.6 (316.04 to 477.28) | 0.9 (0.7 to 1.1) | 381.48 (302.79 to 463.41) | 1.06 (0.93 to 1.19) | -0.14 (-0.2 to -0.09) |
| Venezuela (Bolivarian Republic of) | 35.8 (29.6 to 42.3) | 259.15 (213.72 to 306.18) | 66.7 (53.9 to 80.5) | 223.86 (183.1 to 268.29) | 0.86 (0.7 to 1.05) | -0.56 (-0.59 to -0.53) |
| Viet Nam | 182.2 (143.8 to 217.3) | 328.69 (256.75 to 405.89) | 347.7 (271.1 to 433.3) | 330.38 (260.98 to 408.05) | 0.91 (0.66 to 1.1) | 0.08 (0.03 to 0.13) |
| Yemen | 26.4 (20.8 to 31.9) | 258.05 (206.21 to 313.06) | 69.9 (55.8 to 84.8) | 277.32 (221.58 to 334.65) | 1.65 (1.47 to 1.81) | 0.25 (0.21 to 0.29) |
| Zambia | 19.9 (16.7 to 23.3) | 438.13 (360.12 to 522.23) | 54.8 (44.5 to 65.6) | 498.93 (405.32 to 597.43) | 1.75 (1.57 to 1.93) | 0.38 (0.33 to 0.43) |
| Zimbabwe | 28.3 (23.2 to 33.8) | 443.64 (358.24 to 532.51) | 56.2 (45.7 to 67.8) | 520.53 (424.98 to 625.51) | 0.98 (0.86 to 1.11) | 0.43 (0.17 to 0.68) |

**Supplementary table 5: DALYs of gastritis and duodenitis in 1990 and 2019 for GBD region, with EAPC from 1990 and 2019.**

| location | Num_1990(x10^3^) | ASR_1990/10^5^ | Num_2019(x10^3^) | ASR_2019/10^5^ | Num_change | EAPC_CI |
| --- | --- | --- | --- | --- | --- | --- |
| **Global** | 2216 (1692 to 2919) | 48.14 (36.79 to 63.39) | 2815 (2088 to 3794) | 34.78 (25.87 to 46.8) | 0.27 (0.11 to 0.44) | -1.1 (-1.16 to -1.05) |
| **SDI region** |  |  |  |  |  |  |
| High SDI | 208 (143 to 292) | 22.15 (15.22 to 31.36) | 223 (152 to 314) | 16.43 (10.98 to 23.56) | 0.08 (0.01 to 0.15) | -1.09 (-1.22 to -0.95) |
| High-middle SDI | 444 (323 to 605) | 39.65 (29.07 to 54.14) | 491 (346 to 680) | 27.03 (18.95 to 37.71) | 0.11 (-0.06 to 0.24) | -1.41 (-1.49 to -1.32) |
| Middle SDI | 787 (594 to 1051) | 61.94 (47.86 to 82) | 932 (697 to 1237) | 37.26 (28.06 to 48.91) | 0.18 (-0.02 to 0.38) | -1.62 (-1.7 to -1.54) |
| Low-middle SDI | 505 (377 to 664) | 61.76 (46.49 to 82.09) | 709 (530 to 957) | 44.87 (34 to 59.5) | 0.4 (0.19 to 0.66) | -1.07 (-1.13 to -1.02) |
| Low SDI | 271 (197 to 352) | 73.81 (52.28 to 97.23) | 458 (341 to 606) | 57.16 (42.75 to 74.7) | 0.69 (0.41 to 1) | -0.98 (-1.03 to -0.93) |
| **GBD region** |  |  |  |  |  |  |
| Andean Latin America | 16 (11 to 21) | 53.79 (39.39 to 72.98) | 30 (23 to 40) | 50.71 (38.32 to 66.17) | 0.94 (0.53 to 1.38) | 0.24 (0.04 to 0.43) |
| Australasia | 5 (3 to 7) | 21.4 (14.32 to 31.47) | 7 (5 to 10) | 19.92 (12.74 to 29.63) | 0.5 (0.37 to 0.62) | -0.2 (-0.26 to -0.13) |
| Caribbean | 15 (11 to 20) | 49.87 (37.13 to 65.44) | 23 (17 to 31) | 46.72 (33.87 to 62.14) | 0.56 (0.37 to 0.73) | -0.3 (-0.35 to -0.25) |
| Central Asia | 10 (7 to 14) | 17.09 (11.88 to 23.88) | 16 (10 to 23) | 17.33 (11.67 to 24.8) | 0.53 (0.4 to 0.64) | -0.01 (-0.05 to 0.03) |
| Central Europe | 42 (28 to 60) | 31.21 (21.04 to 45.02) | 50 (35 to 68) | 31.46 (21.79 to 44.01) | 0.19 (0.07 to 0.3) | 0.25 (0.19 to 0.32) |
| Central Latin America | 48 (40 to 58) | 44.73 (38.08 to 53.55) | 71 (58 to 86) | 29.32 (24.23 to 35.64) | 0.48 (0.36 to 0.63) | -1.72 (-1.81 to -1.63) |
| Central Sub-Saharan Africa | 39 (25 to 53) | 118.15 (78.49 to 159.67) | 74 (52 to 100) | 98.54 (67.56 to 132.39) | 0.92 (0.5 to 1.43) | -0.64 (-0.71 to -0.57) |
| East Asia | 881 (656 to 1172) | 90.77 (68.69 to 120.34) | 861 (651 to 1119) | 43.96 (33.37 to 56.76) | -0.02 (-0.26 to 0.21) | -2.4 (-2.51 to -2.28) |
| Eastern Europe | 54 (38 to 75) | 21.19 (15 to 29.53) | 62 (44 to 86) | 23.06 (16.36 to 32.55) | 0.15 (0.08 to 0.21) | 0.24 (0.16 to 0.33) |
| Eastern Sub-Saharan Africa | 122 (82 to 160) | 109.9 (71.43 to 146.85) | 199 (141 to 262) | 81.75 (57.21 to 105.8) | 0.63 (0.36 to 0.97) | -1.2 (-1.28 to -1.12) |
| High-income Asia Pacific | 31 (23 to 39) | 17.15 (12.77 to 21.69) | 17 (12 to 25) | 7.46 (4.9 to 10.92) | -0.43 (-0.54 to -0.18) | -3.5 (-3.95 to -3.05) |
| High-income North America | 91 (62 to 131) | 28.9 (19.39 to 41.57) | 82 (55 to 113) | 17.39 (11.64 to 24.55) | -0.11 (-0.19 to 0) | -1.81 (-1.98 to -1.63) |
| North Africa and Middle East | 52 (34 to 76) | 18.42 (12.33 to 26.87) | 105 (68 to 154) | 18.22 (12 to 26.65) | 1.03 (0.85 to 1.2) | 0.17 (0.1 to 0.24) |
| Oceania | 2 (1 to 2) | 33.03 (22.69 to 47.01) | 3 (2 to 5) | 29.57 (19.25 to 42.66) | 1 (0.8 to 1.18) | -0.45 (-0.54 to -0.37) |
| South Asia | 391 (275 to 531) | 47.36 (33.6 to 65.21) | 646 (445 to 916) | 37.73 (26.26 to 52.97) | 0.65 (0.35 to 0.93) | -0.8 (-0.93 to -0.67) |
| Southeast Asia | 130 (90 to 181) | 32.81 (23.63 to 44.64) | 177 (118 to 256) | 25.97 (17.67 to 37.11) | 0.36 (0.14 to 0.56) | -0.79 (-0.88 to -0.69) |
| Southern Latin America | 12 (9 to 16) | 25.76 (19 to 34.51) | 14 (9 to 19) | 18.36 (12.65 to 26.38) | 0.11 (-0.02 to 0.25) | -1.61 (-1.78 to -1.44) |
| Southern Sub-Saharan Africa | 33 (25 to 41) | 81.58 (62.55 to 99.74) | 51 (41 to 63) | 72.81 (59.2 to 89.17) | 0.53 (0.29 to 0.9) | -0.09 (-0.61 to 0.44) |
| Tropical Latin America | 65 (45 to 94) | 52.51 (36.33 to 74.29) | 69 (49 to 96) | 28.39 (20.4 to 39.45) | 0.05 (-0.02 to 0.14) | -2.46 (-2.66 to -2.25) |
| Western Europe | 90 (60 to 128) | 18.89 (12.69 to 27.26) | 112 (76 to 159) | 17.91 (11.91 to 25.9) | 0.25 (0.2 to 0.3) | -0.14 (-0.19 to -0.1) |
| Western Sub-Saharan Africa | 88 (59 to 123) | 54.66 (36.11 to 78.57) | 148 (106 to 199) | 41 (30.28 to 55.48) | 0.67 (0.3 to 1.13) | -1.01 (-1.05 to -0.97) |

**Supplementary table 6:** **DALYs of gastritis and duodenitis in 1990 and 2019 for all locations, with EAPC from 1990 and 2019.**

| location | Num_1990(x10^3^) | ASR_1990/10^5^ | Num_2019(x10^3^) | ASR_2019/10^5^ | Num_change | EAPC_CI |
| --- | --- | --- | --- | --- | --- | --- |
| Afghanistan | 2.4 (1.6 to 3.5) | 26.47 (17.6 to 39.15) | 7.6 (5 to 11.2) | 28.78 (19.56 to 41.46) | 2.17 (1.52 to 2.68) | 0.41 (0.33 to 0.5) |
| Albania | 0.7 (0.4 to 1) | 24.14 (15.46 to 35.52) | 0.8 (0.5 to 1.2) | 24.16 (15.14 to 36.3) | 0.16 (0 to 0.34) | -0.04 (-0.13 to 0.05) |
| Algeria | 3.1 (2 to 4.6) | 15.81 (10.52 to 22.74) | 6.7 (4.2 to 9.8) | 16.3 (10.36 to 23.92) | 1.16 (0.87 to 1.49) | 0.17 (0.11 to 0.22) |
| American Samoa | 0 (0 to 0) | 33.01 (22.98 to 44.99) | 0 (0 to 0) | 24.83 (16.81 to 35.77) | 0.18 (0.01 to 0.35) | -1.49 (-1.77 to -1.2) |
| Andorra | 0 (0 to 0) | 15.36 (9.81 to 22.98) | 0 (0 to 0) | 16.33 (10.27 to 24.36) | 1.12 (0.9 to 1.37) | 0.29 (0.26 to 0.31) |
| Angola | 7.2 (4.6 to 10.7) | 120.83 (76.52 to 172.69) | 14.8 (10.1 to 20.4) | 93.92 (62.16 to 130.33) | 1.06 (0.36 to 2.19) | -0.93 (-1.04 to -0.81) |
| Antigua and Barbuda | 0 (0 to 0) | 44.45 (33.65 to 57.87) | 0 (0 to 0.1) | 42.23 (31.09 to 56.82) | 0.78 (0.52 to 1.03) | -0.17 (-0.24 to -0.1) |
| Argentina | 7.9 (5.6 to 10.8) | 24.27 (17.46 to 33.24) | 9 (6.1 to 12.9) | 18.48 (12.56 to 26.66) | 0.15 (-0.01 to 0.29) | -0.98 (-1.15 to -0.81) |
| Armenia | 0.5 (0.3 to 0.7) | 15.85 (11.2 to 22.25) | 0.7 (0.5 to 0.9) | 18.54 (13.23 to 25.22) | 0.35 (0.17 to 0.55) | 0.72 (0.59 to 0.85) |
| Australia | 3.8 (2.5 to 5.6) | 20.63 (13.59 to 30.58) | 5.8 (3.7 to 8.6) | 19.37 (12.24 to 29.19) | 0.53 (0.38 to 0.67) | -0.19 (-0.22 to -0.15) |
| Austria | 1.6 (1.1 to 2.4) | 17.05 (11.27 to 24.33) | 2.2 (1.5 to 3.2) | 17.43 (11.35 to 25.84) | 0.36 (0.23 to 0.49) | 0.38 (0.26 to 0.5) |
| Azerbaijan | 1.1 (0.7 to 1.5) | 15.84 (10.69 to 22.47) | 1.7 (1 to 2.5) | 14.94 (9.58 to 21.8) | 0.58 (0.35 to 0.81) | -0.43 (-0.54 to -0.32) |
| Bahamas | 0.1 (0.1 to 0.1) | 40.89 (30.94 to 53.79) | 0.2 (0.1 to 0.2) | 38.78 (28.22 to 52.43) | 0.95 (0.64 to 1.26) | -0.26 (-0.31 to -0.21) |
| Bahrain | 0.1 (0 to 0.1) | 21.36 (15.4 to 28.92) | 0.2 (0.2 to 0.4) | 16.61 (11.26 to 23.67) | 2.35 (1.77 to 3.03) | -1.64 (-2.03 to -1.24) |
| Bangladesh | 37.9 (26.4 to 53) | 55.71 (39.05 to 79.86) | 47.2 (30.3 to 68.6) | 30.78 (19.94 to 44.61) | 0.25 (-0.03 to 0.5) | -2.74 (-3.08 to -2.4) |
| Barbados | 0.1 (0.1 to 0.1) | 27.45 (19.72 to 37.95) | 0.1 (0.1 to 0.2) | 28.59 (19.63 to 40.3) | 0.61 (0.42 to 0.79) | 0.05 (-0.01 to 0.11) |
| Belarus | 2.1 (1.4 to 3.1) | 18.16 (12.24 to 26.33) | 2.3 (1.5 to 3.3) | 18.52 (12.13 to 27) | 0.07 (-0.06 to 0.2) | 0.05 (0 to 0.09) |
| Belgium | 2.1 (1.3 to 3.2) | 17.25 (10.93 to 25.53) | 2.9 (1.8 to 4.2) | 18.38 (11.54 to 27.62) | 0.35 (0.23 to 0.47) | 0.18 (0.13 to 0.23) |
| Belize | 0.1 (0 to 0.1) | 50.03 (38.02 to 65.33) | 0.2 (0.1 to 0.2) | 51.21 (37.41 to 67.97) | 1.75 (1.41 to 2.12) | -0.03 (-0.19 to 0.13) |
| Benin | 2.1 (1.3 to 3.3) | 52.32 (33.64 to 72.11) | 3.8 (2.6 to 5.6) | 40.45 (28.56 to 55.23) | 0.84 (0.13 to 1.94) | -0.95 (-1.05 to -0.86) |
| Bermuda | 0 (0 to 0) | 31.99 (23.08 to 43.26) | 0 (0 to 0) | 30.41 (20.71 to 43.91) | 0.44 (0.22 to 0.66) | -0.17 (-0.23 to -0.1) |
| Bhutan | 0.2 (0.1 to 0.3) | 48.9 (32.16 to 74.41) | 0.2 (0.1 to 0.3) | 30.84 (20.75 to 43.42) | 0.07 (-0.17 to 0.32) | -1.72 (-1.86 to -1.58) |
| Bolivia (Plurinational State of) | 2.8 (1.9 to 4.1) | 61.81 (41.26 to 86.7) | 5.5 (4 to 7.3) | 54.95 (40.36 to 72.41) | 0.92 (0.47 to 1.47) | -0.38 (-0.43 to -0.32) |
| Bosnia and Herzegovina | 1.1 (0.7 to 1.6) | 24.41 (16.57 to 35.13) | 1 (0.7 to 1.5) | 22.87 (14.72 to 33.17) | -0.08 (-0.19 to 0.05) | -0.34 (-0.5 to -0.19) |
| Botswana | 0.9 (0.7 to 1.3) | 107.86 (77 to 149.99) | 1.5 (1 to 2) | 72.54 (52.32 to 97.7) | 0.56 (0.15 to 1.09) | -1.93 (-2.27 to -1.59) |
| Brazil | 64.1 (43.8 to 91.5) | 52.78 (36.62 to 74.65) | 67 (48 to 92.9) | 28.35 (20.41 to 39.28) | 0.04 (-0.03 to 0.13) | -2.49 (-2.69 to -2.28) |
| Brunei Darussalam | 0 (0 to 0) | 17.17 (12.24 to 22.98) | 0 (0 to 0.1) | 9.87 (6.93 to 13.66) | 0.45 (0.18 to 0.73) | -2.55 (-2.84 to -2.26) |
| Bulgaria | 2.5 (1.7 to 3.5) | 23.81 (16.17 to 33.5) | 3.7 (2.6 to 4.9) | 34.46 (24.48 to 46.15) | 0.48 (0.2 to 0.77) | 1.73 (1.56 to 1.9) |
| Burkina Faso | 3 (1.9 to 4.7) | 39.44 (27.08 to 56) | 6.4 (4.3 to 9.2) | 37.23 (25.36 to 52.57) | 1.13 (0.32 to 2.15) | -0.18 (-0.32 to -0.04) |
| Burundi | 3.5 (2.2 to 4.9) | 106.06 (65.38 to 156.32) | 7.3 (4.7 to 10.9) | 107.22 (68.96 to 159.26) | 1.08 (0.45 to 1.95) | -0.11 (-0.29 to 0.08) |
| Cabo Verde | 0.1 (0.1 to 0.1) | 35.3 (25.98 to 49.41) | 0.1 (0.1 to 0.2) | 20.81 (14.04 to 29.38) | 0.16 (-0.21 to 0.56) | -1.55 (-2.03 to -1.07) |
| Cambodia | 6 (3.2 to 9.6) | 73.59 (43.93 to 101.48) | 5.9 (4.1 to 8.2) | 42.15 (29.37 to 56.79) | -0.02 (-0.41 to 0.56) | -2.1 (-2.22 to -1.98) |
| Cameroon | 3.6 (2.4 to 5) | 46.73 (32.23 to 61.68) | 7.2 (4.9 to 9.9) | 33.48 (22.99 to 46) | 1.02 (0.33 to 1.84) | -1.41 (-1.53 to -1.28) |
| Canada | 6.8 (4.8 to 9.4) | 22.1 (15.59 to 30.54) | 9 (6.1 to 12.8) | 17.89 (12.05 to 25.7) | 0.33 (0.18 to 0.5) | -0.8 (-0.9 to -0.71) |
| Central African Republic | 2.9 (1.8 to 4.9) | 177.59 (108.59 to 290.64) | 5.1 (3.1 to 8.9) | 162.44 (100.26 to 272.14) | 0.71 (0.22 to 1.35) | -0.37 (-0.43 to -0.31) |
| Chad | 3.4 (2 to 5.3) | 68.48 (43.08 to 97.04) | 6.3 (4.2 to 9) | 53.7 (38.16 to 71.86) | 0.85 (0.1 to 1.91) | -0.95 (-1.04 to -0.85) |
| Chile | 3.5 (2.7 to 4.5) | 30.66 (23.44 to 39.01) | 3.8 (2.6 to 5.4) | 18.13 (12.3 to 25.81) | 0.09 (-0.09 to 0.31) | -3.1 (-3.45 to -2.75) |
| China | 864.1 (644.6 to 1149.6) | 92.42 (69.7 to 122.68) | 836.3 (634.4 to 1086) | 44.27 (33.63 to 57.06) | -0.03 (-0.27 to 0.2) | -2.43 (-2.55 to -2.31) |
| Colombia | 5.7 (4.3 to 7.5) | 24.74 (19.28 to 31.81) | 10.5 (7.8 to 13.9) | 20.3 (15.1 to 26.93) | 0.84 (0.6 to 1.11) | -0.58 (-0.9 to -0.26) |
| Comoros | 0.3 (0.2 to 0.5) | 119.28 (64.97 to 187.13) | 0.5 (0.3 to 0.7) | 90.92 (61.39 to 129.12) | 0.54 (0.07 to 1.74) | -1.21 (-1.39 to -1.03) |
| Congo | 1.7 (1.1 to 2.4) | 121.64 (79.57 to 165.2) | 2.8 (1.9 to 3.9) | 83.19 (56.02 to 115.64) | 0.65 (0.19 to 1.2) | -1.31 (-1.48 to -1.14) |
| Cook Islands | 0 (0 to 0) | 22.14 (14.39 to 32.97) | 0 (0 to 0) | 20.1 (12.72 to 30.11) | 0.16 (0.01 to 0.35) | -0.36 (-0.44 to -0.29) |
| Costa Rica | 0.4 (0.3 to 0.6) | 18.79 (13.53 to 25.47) | 0.7 (0.5 to 1) | 13.65 (9.3 to 19.76) | 0.67 (0.45 to 0.93) | -1.64 (-1.83 to -1.44) |
| Côte d'Ivoire | 4.3 (2.9 to 6.4) | 47.13 (32.22 to 62.65) | 7.5 (5.2 to 10.2) | 36.42 (25.95 to 49.53) | 0.72 (0.15 to 1.4) | -1.05 (-1.18 to -0.92) |
| Croatia | 1 (0.7 to 1.5) | 18.03 (11.66 to 26.89) | 1.3 (0.9 to 1.7) | 19.94 (13.72 to 27.89) | 0.22 (0.02 to 0.45) | 0.16 (-0.02 to 0.34) |
| Cuba | 4.3 (3.2 to 5.8) | 41.09 (30.17 to 55.4) | 6.4 (4.6 to 8.7) | 39.74 (28.06 to 54.13) | 0.49 (0.31 to 0.68) | -0.3 (-0.38 to -0.23) |
| Cyprus | 0.1 (0.1 to 0.1) | 11.96 (7.64 to 17.15) | 0.2 (0.1 to 0.3) | 11.34 (7.12 to 17.06) | 1.02 (0.77 to 1.29) | -0.93 (-1.33 to -0.54) |
| Czechia | 2.2 (1.5 to 3.2) | 18.96 (12.81 to 27.07) | 3.1 (2.1 to 4.5) | 21.63 (14.29 to 31.47) | 0.4 (0.24 to 0.55) | 0.75 (0.52 to 0.99) |
| Democratic People's Republic of Korea | 10.5 (7 to 14.8) | 58.23 (39.51 to 81.46) | 15.7 (10.7 to 22.7) | 50.57 (34.78 to 72.42) | 0.49 (0.2 to 0.85) | -0.37 (-0.48 to -0.26) |
| Democratic Republic of the Congo | 25.7 (16.6 to 35.9) | 113.3 (72.64 to 153.94) | 49.9 (33.8 to 68.9) | 98.76 (66.53 to 135.45) | 0.94 (0.46 to 1.52) | -0.46 (-0.56 to -0.36) |
| Denmark | 1 (0.7 to 1.4) | 14.83 (9.95 to 21) | 1.5 (1 to 2.1) | 17.62 (11.73 to 25.11) | 0.55 (0.39 to 0.73) | 0.65 (0.52 to 0.77) |
| Djibouti | 0.2 (0.2 to 0.3) | 94.57 (59.79 to 135.44) | 0.6 (0.4 to 0.9) | 71.7 (46.44 to 100.33) | 1.6 (0.88 to 2.53) | -1.18 (-1.29 to -1.08) |
| Dominica | 0 (0 to 0) | 52.99 (40.82 to 68.24) | 0 (0 to 0) | 42.64 (31 to 58.08) | -0.02 (-0.21 to 0.17) | -0.89 (-0.97 to -0.81) |
| Dominican Republic | 2 (1.4 to 2.8) | 35.56 (25.38 to 49.6) | 3.9 (2.7 to 5.5) | 37.44 (25.96 to 52.88) | 0.97 (0.71 to 1.26) | 0.16 (0.06 to 0.25) |
| Ecuador | 3 (2.1 to 4.2) | 39.75 (27.24 to 55.81) | 5.6 (4.2 to 7.5) | 34.87 (25.96 to 45.64) | 0.87 (0.5 to 1.27) | -0.24 (-0.33 to -0.15) |
| Egypt | 7.6 (4.9 to 11.1) | 16.25 (10.6 to 23.75) | 14.3 (9.2 to 21.1) | 15.83 (10.29 to 23.07) | 0.88 (0.63 to 1.13) | -0.01 (-0.08 to 0.07) |
| El Salvador | 1 (0.7 to 1.3) | 26.59 (19.69 to 34.72) | 1.1 (0.8 to 1.5) | 17.72 (12.73 to 24.5) | 0.1 (-0.06 to 0.26) | -1.62 (-1.94 to -1.3) |
| Equatorial Guinea | 0.4 (0.2 to 0.7) | 151.78 (86.08 to 278.75) | 0.5 (0.3 to 0.7) | 68.79 (46.68 to 94.83) | 0.21 (-0.3 to 1.01) | -3.13 (-3.32 to -2.94) |
| Eritrea | 2 (1.3 to 2.9) | 118.34 (76 to 171.53) | 4.7 (3 to 6.8) | 115.97 (73.04 to 165.69) | 1.4 (0.67 to 2.49) | -0.11 (-0.2 to -0.02) |
| Estonia | 0.3 (0.2 to 0.4) | 16.78 (11.14 to 24.47) | 0.3 (0.2 to 0.4) | 17.62 (11.54 to 25.75) | 0.02 (-0.08 to 0.14) | 0.18 (0.08 to 0.27) |
| Eswatini | 0.4 (0.3 to 0.5) | 80.19 (60.3 to 104.06) | 0.7 (0.5 to 0.9) | 74.43 (54.52 to 100.23) | 0.55 (0.13 to 1.21) | -0.08 (-0.35 to 0.19) |
| Ethiopia | 45.8 (28.8 to 66.9) | 151.27 (91.64 to 219.73) | 48 (31.9 to 66) | 76 (49.29 to 105.82) | 0.05 (-0.3 to 0.52) | -2.75 (-2.9 to -2.59) |
| Fiji | 0.2 (0.1 to 0.3) | 31.22 (21.42 to 43.45) | 0.2 (0.1 to 0.3) | 23.57 (15.54 to 34.18) | 0.17 (0.01 to 0.36) | -1.28 (-1.44 to -1.11) |
| Finland | 1.3 (0.9 to 1.7) | 20.56 (14.99 to 27.08) | 1.5 (1.1 to 2) | 18.15 (12.73 to 24.97) | 0.14 (0 to 0.3) | -0.67 (-0.92 to -0.42) |
| France | 10.9 (7.1 to 15.8) | 15.9 (10.33 to 23.25) | 14 (8.7 to 20.9) | 15.71 (9.69 to 23.87) | 0.29 (0.15 to 0.44) | 0.05 (-0.01 to 0.1) |
| Gabon | 0.6 (0.4 to 0.8) | 88.02 (59.01 to 122.8) | 0.8 (0.6 to 1.1) | 63.79 (44.06 to 86.2) | 0.4 (0.05 to 0.81) | -1.17 (-1.3 to -1.03) |
| Gambia | 0.5 (0.3 to 0.7) | 63.7 (38.12 to 96) | 0.6 (0.4 to 0.8) | 37.07 (25.68 to 49.98) | 0.3 (-0.23 to 1.09) | -2.29 (-2.47 to -2.1) |
| Georgia | 1 (0.7 to 1.3) | 16.47 (11.71 to 22.62) | 0.8 (0.5 to 1.1) | 17.37 (11.91 to 24.21) | -0.19 (-0.28 to -0.08) | 0.38 (0.15 to 0.61) |
| Germany | 18.7 (12.7 to 26.4) | 18.01 (12.14 to 25.54) | 26.1 (18.3 to 36) | 19.61 (13.31 to 27.79) | 0.4 (0.26 to 0.55) | 0.5 (0.43 to 0.58) |
| Ghana | 3.4 (2.4 to 4.6) | 29.45 (21.32 to 39.66) | 7.4 (5.1 to 10.5) | 28.2 (19.53 to 40.07) | 1.19 (0.69 to 1.72) | -0.24 (-0.33 to -0.15) |
| Greece | 2 (1.2 to 2.9) | 15.36 (9.72 to 22.78) | 2.4 (1.5 to 3.6) | 15.99 (9.89 to 23.98) | 0.21 (0.1 to 0.33) | 0.23 (0.18 to 0.28) |
| Greenland | 0 (0 to 0) | 36.18 (20.66 to 48.04) | 0 (0 to 0) | 18.65 (12.91 to 26.97) | -0.27 (-0.46 to 0.03) | -3.25 (-3.75 to -2.76) |
| Grenada | 0 (0 to 0.1) | 69.48 (52.64 to 87.41) | 0.1 (0 to 0.1) | 53.86 (41.23 to 68.89) | 0.26 (0.03 to 0.55) | -0.97 (-1.09 to -0.84) |
| Guam | 0 (0 to 0) | 22.42 (14.58 to 32.79) | 0 (0 to 0.1) | 20.92 (12.99 to 31.56) | 0.49 (0.29 to 0.7) | -0.35 (-0.44 to -0.25) |
| Guatemala | 2.8 (2.2 to 3.5) | 55.43 (43.49 to 67.03) | 6.2 (4.8 to 7.9) | 47.93 (37.64 to 60.81) | 1.2 (0.73 to 1.81) | -0.62 (-0.74 to -0.5) |
| Guinea | 3.3 (1.9 to 5.4) | 57.56 (36.4 to 80.68) | 4.2 (2.7 to 6.2) | 42.94 (28.27 to 60.17) | 0.28 (-0.24 to 1.03) | -1.02 (-1.08 to -0.97) |
| Guinea-Bissau | 0.6 (0.4 to 1) | 79.72 (50.54 to 117.21) | 0.7 (0.5 to 1) | 53.18 (38.71 to 71.9) | 0.16 (-0.3 to 0.82) | -1.36 (-1.41 to -1.31) |
| Guyana | 0.3 (0.2 to 0.4) | 59.68 (46.73 to 75.24) | 0.4 (0.3 to 0.5) | 57.13 (42.93 to 74.79) | 0.27 (0.05 to 0.5) | -0.03 (-0.13 to 0.07) |
| Haiti | 4.6 (2.6 to 7.1) | 98.87 (60.9 to 143.04) | 7.1 (4.9 to 9.6) | 75.44 (52.71 to 104.02) | 0.53 (0.11 to 1.11) | -0.85 (-0.92 to -0.79) |
| Honduras | 2.3 (1.6 to 3) | 77.98 (54.28 to 98.8) | 4.4 (3 to 6.1) | 66.67 (46.12 to 92.71) | 0.88 (0.39 to 1.48) | -0.53 (-0.67 to -0.39) |
| Hungary | 2.6 (1.8 to 3.7) | 21.59 (14.4 to 30.64) | 3.5 (2.4 to 4.9) | 25.75 (17.63 to 36.4) | 0.33 (0.08 to 0.52) | 0.54 (0.35 to 0.74) |
| Iceland | 0 (0 to 0.1) | 15.01 (10.32 to 20.88) | 0.1 (0 to 0.1) | 14.39 (9.38 to 20.97) | 0.59 (0.41 to 0.8) | -0.09 (-0.12 to -0.05) |
| India | 318.9 (221.8 to 436.7) | 48.59 (33.9 to 67.41) | 532.1 (370.9 to 751.4) | 39.4 (27.56 to 55.25) | 0.67 (0.35 to 0.98) | -0.68 (-0.82 to -0.54) |
| Indonesia | 42 (27.8 to 61.2) | 25.55 (17.07 to 36.71) | 59.8 (37.5 to 89.7) | 22.65 (14.41 to 33.66) | 0.42 (0.24 to 0.61) | -0.33 (-0.46 to -0.2) |
| Iran (Islamic Republic of) | 10.1 (6.6 to 14.9) | 22.11 (14.92 to 31.61) | 18.3 (11.9 to 26.8) | 21.24 (14.14 to 30.77) | 0.8 (0.58 to 1.03) | 0.59 (0.4 to 0.77) |
| Iraq | 2.4 (1.6 to 3.4) | 17.55 (11.59 to 25.07) | 6.1 (3.9 to 9) | 16.76 (10.98 to 24.53) | 1.57 (1.21 to 1.91) | -0.03 (-0.12 to 0.06) |
| Ireland | 0.7 (0.4 to 1) | 17.85 (11.97 to 25.77) | 1.1 (0.8 to 1.7) | 18.08 (12.07 to 26.59) | 0.72 (0.56 to 0.88) | -0.04 (-0.21 to 0.12) |
| Israel | 0.8 (0.5 to 1.1) | 16.52 (10.62 to 24.19) | 1.6 (1 to 2.4) | 16.48 (10.3 to 24.51) | 1.12 (0.9 to 1.33) | -0.02 (-0.07 to 0.04) |
| Italy | 17.3 (11.8 to 24.6) | 23.85 (16.18 to 34.37) | 12.6 (8.7 to 17.6) | 13.97 (9.43 to 20.07) | -0.27 (-0.32 to -0.21) | -2.17 (-2.31 to -2.04) |
| Jamaica | 0.6 (0.4 to 0.9) | 31.84 (22.23 to 44.71) | 1.1 (0.8 to 1.5) | 37.02 (26.45 to 51.67) | 0.74 (0.51 to 0.97) | 0.75 (0.65 to 0.85) |
| Japan | 17.2 (12.6 to 23.2) | 12.52 (9.19 to 16.96) | 11.5 (7.7 to 16.3) | 7.2 (4.68 to 10.53) | -0.33 (-0.42 to -0.14) | -2.09 (-2.39 to -1.8) |
| Jordan | 0.3 (0.2 to 0.5) | 11.8 (7.59 to 17.31) | 1.4 (0.9 to 2.1) | 13.43 (8.49 to 20.19) | 3.24 (2.67 to 3.86) | 0.61 (0.51 to 0.71) |
| Kazakhstan | 2.7 (1.9 to 3.9) | 18.17 (12.46 to 25.87) | 3.4 (2.2 to 4.8) | 17.64 (11.75 to 24.85) | 0.22 (-0.02 to 0.4) | -0.34 (-0.45 to -0.23) |
| Kenya | 13.9 (8.7 to 20.7) | 114.55 (68.65 to 179.94) | 29.6 (20.1 to 39.9) | 93.79 (62.69 to 127.09) | 1.14 (0.63 to 1.69) | -0.52 (-0.59 to -0.44) |
| Kiribati | 0 (0 to 0) | 44.85 (32.11 to 62.67) | 0 (0 to 0) | 32.78 (22.58 to 45.19) | 0.34 (0.1 to 0.56) | -1.27 (-1.39 to -1.15) |
| Kuwait | 0.2 (0.1 to 0.3) | 13.02 (8.11 to 19.63) | 0.7 (0.4 to 1) | 14.01 (8.82 to 21.14) | 2.26 (1.79 to 2.78) | 0.22 (0.19 to 0.25) |
| Kyrgyzstan | 0.7 (0.5 to 0.9) | 17.72 (12.49 to 24.77) | 1 (0.6 to 1.5) | 16.25 (10.43 to 23.95) | 0.48 (0.26 to 0.7) | -0.48 (-0.6 to -0.35) |
| Lao People's Democratic Republic | 2 (1.2 to 2.9) | 64.07 (40.69 to 86.66) | 2.4 (1.6 to 3.4) | 41.59 (27.38 to 58.32) | 0.2 (-0.18 to 0.82) | -1.6 (-1.67 to -1.54) |
| Latvia | 0.7 (0.5 to 0.9) | 22.07 (15.74 to 30.17) | 0.5 (0.4 to 0.8) | 20.89 (14.31 to 29.34) | -0.21 (-0.31 to -0.1) | -0.67 (-1 to -0.34) |
| Lebanon | 0.5 (0.3 to 0.7) | 15.86 (10.36 to 23.66) | 0.9 (0.6 to 1.3) | 16.66 (10.78 to 24.66) | 0.92 (0.69 to 1.21) | 0.35 (0.22 to 0.48) |
| Lesotho | 1.3 (1 to 1.7) | 100.04 (73.51 to 130.45) | 1.8 (1.2 to 2.4) | 102.81 (71.9 to 137.59) | 0.38 (-0.05 to 0.91) | 0.36 (0.18 to 0.54) |
| Liberia | 1 (0.6 to 1.9) | 54.85 (34.07 to 85.99) | 1.4 (0.9 to 2) | 36.44 (25.04 to 50.91) | 0.31 (-0.28 to 1.27) | -1.39 (-1.59 to -1.18) |
| Libya | 0.5 (0.3 to 0.7) | 15.48 (10.12 to 22.52) | 1.1 (0.7 to 1.7) | 16.85 (11.02 to 24.65) | 1.31 (0.96 to 1.68) | 0.41 (0.34 to 0.49) |
| Lithuania | 0.7 (0.4 to 1) | 15.99 (10.58 to 23.62) | 0.8 (0.6 to 1.1) | 21.12 (14.52 to 29.58) | 0.27 (0.1 to 0.48) | 1.4 (1.24 to 1.56) |
| Luxembourg | 0.1 (0.1 to 0.2) | 24.91 (16.34 to 36.45) | 0.2 (0.1 to 0.3) | 24.75 (15.65 to 37.41) | 0.73 (0.56 to 0.89) | -0.49 (-0.67 to -0.3) |
| Madagascar | 6.5 (4.3 to 8.6) | 84.24 (53.95 to 117.31) | 14.3 (9.8 to 19) | 87.05 (58.69 to 116.62) | 1.22 (0.62 to 2.11) | 0.19 (0.1 to 0.28) |
| Malawi | 5.9 (3.9 to 7.9) | 102.45 (65.24 to 137.9) | 9.4 (6.6 to 13) | 87.11 (62.43 to 118.47) | 0.59 (0.14 to 1.17) | -0.81 (-0.97 to -0.66) |
| Malaysia | 3.1 (2 to 4.5) | 20.77 (13.81 to 29.96) | 5.9 (3.8 to 8.7) | 19.1 (12.2 to 28.02) | 0.93 (0.71 to 1.16) | -0.35 (-0.41 to -0.29) |
| Maldives | 0.1 (0 to 0.1) | 28.48 (18.19 to 42.58) | 0.1 (0.1 to 0.2) | 22.86 (13.93 to 34.97) | 1.12 (0.78 to 1.46) | -0.68 (-0.74 to -0.62) |
| Mali | 5.1 (2.9 to 8.5) | 64.54 (40.82 to 92.04) | 8.3 (5.2 to 12.9) | 49.49 (33.41 to 69.68) | 0.63 (-0.1 to 1.79) | -0.88 (-1.06 to -0.7) |
| Malta | 0.1 (0.1 to 0.2) | 28.26 (19.45 to 40.88) | 0.2 (0.1 to 0.3) | 28.11 (18.82 to 41.17) | 0.54 (0.38 to 0.71) | 0.37 (0.25 to 0.48) |
| Marshall Islands | 0 (0 to 0) | 36.27 (25.57 to 50.14) | 0 (0 to 0) | 26.57 (17.37 to 37.54) | 0.22 (0.04 to 0.43) | -1.22 (-1.35 to -1.1) |
| Mauritania | 0.9 (0.5 to 1.2) | 58.08 (35.61 to 79.92) | 1 (0.7 to 1.4) | 31.93 (22.31 to 45.17) | 0.08 (-0.28 to 0.57) | -2.26 (-2.38 to -2.14) |
| Mauritius | 0.3 (0.2 to 0.3) | 27.51 (20.39 to 36.57) | 0.4 (0.2 to 0.5) | 22.65 (16.04 to 31.45) | 0.37 (0.19 to 0.57) | -0.6 (-0.88 to -0.33) |
| Mexico | 29.6 (25 to 35.6) | 55.68 (48.18 to 65.1) | 38.3 (32.3 to 46.7) | 32.34 (27.29 to 39.15) | 0.29 (0.15 to 0.47) | -2.29 (-2.43 to -2.16) |
| Micronesia (Federated States of) | 0 (0 to 0) | 37.28 (26.32 to 50.87) | 0 (0 to 0) | 27.18 (17.9 to 37.98) | -0.07 (-0.22 to 0.08) | -1.23 (-1.4 to -1.06) |
| Monaco | 0 (0 to 0) | 15.29 (9.5 to 22.59) | 0 (0 to 0) | 15.87 (9.82 to 23.87) | 0.31 (0.18 to 0.45) | 0.22 (0.18 to 0.26) |
| Mongolia | 0.5 (0.3 to 0.7) | 33.67 (23.79 to 46.12) | 0.8 (0.6 to 1.1) | 29.99 (21.6 to 40.66) | 0.61 (0.04 to 1.15) | -0.36 (-0.43 to -0.28) |
| Montenegro | 0.1 (0.1 to 0.2) | 20.48 (12.82 to 30.98) | 0.2 (0.1 to 0.3) | 22.54 (14.01 to 33.93) | 0.32 (0.2 to 0.46) | 0.31 (0.27 to 0.35) |
| Morocco | 4.3 (2.8 to 6.2) | 20.3 (13.29 to 29.65) | 6.7 (4.4 to 9.7) | 18.57 (12.36 to 26.56) | 0.57 (0.36 to 0.78) | -0.2 (-0.27 to -0.13) |
| Mozambique | 7 (4.6 to 9.8) | 85.24 (54.54 to 118.27) | 15.2 (10.1 to 21.9) | 92.7 (60.89 to 134.95) | 1.16 (0.6 to 1.86) | 0.52 (0.39 to 0.64) |
| Myanmar | 18.2 (11.4 to 29.9) | 50.84 (33.24 to 75.49) | 17.6 (12 to 24.7) | 32.79 (22.65 to 45.92) | -0.04 (-0.4 to 0.35) | -1.64 (-1.69 to -1.58) |
| Namibia | 0.9 (0.6 to 1.1) | 91.4 (66.33 to 118.81) | 1.3 (0.9 to 1.7) | 66.51 (47.48 to 88.62) | 0.41 (0.05 to 0.87) | -1.21 (-1.36 to -1.05) |
| Nauru | 0 (0 to 0) | 30.85 (21.43 to 42.55) | 0 (0 to 0) | 26.07 (17.28 to 37.16) | -0.09 (-0.19 to 0.03) | -0.65 (-0.7 to -0.6) |
| Nepal | 4.6 (3 to 6.6) | 31.04 (20.44 to 45.34) | 8 (5.1 to 11.9) | 29.08 (18.68 to 43.25) | 0.75 (0.57 to 0.95) | -0.31 (-0.4 to -0.21) |
| Netherlands | 3.1 (2.1 to 4.4) | 17.54 (11.89 to 25.27) | 4 (2.6 to 5.9) | 16.6 (10.52 to 24.64) | 0.29 (0.15 to 0.44) | -0.25 (-0.3 to -0.2) |
| New Zealand | 0.9 (0.6 to 1.3) | 25.24 (16.9 to 36.71) | 1.2 (0.8 to 1.8) | 23.04 (15.3 to 33.01) | 0.35 (0.22 to 0.5) | -0.2 (-0.42 to 0.03) |
| Nicaragua | 0.8 (0.6 to 1.1) | 40.69 (31.59 to 51.15) | 1.4 (1 to 1.8) | 27.21 (20.62 to 34.61) | 0.62 (0.37 to 0.92) | -1.5 (-1.73 to -1.28) |
| Niger | 5.1 (2.8 to 9.9) | 72.05 (44.9 to 108.65) | 9.6 (5.7 to 16) | 56.62 (36.45 to 82.36) | 0.87 (-0.03 to 2.44) | -0.89 (-1.04 to -0.74) |
| Nigeria | 45.5 (28.1 to 70.8) | 58.72 (36.22 to 95.8) | 73.4 (48.5 to 110.4) | 42.23 (28.47 to 63.78) | 0.61 (0.17 to 1.25) | -1.09 (-1.13 to -1.06) |
| Niue | 0 (0 to 0) | 28.09 (19.22 to 39.14) | 0 (0 to 0) | 22.88 (15.05 to 33.11) | -0.28 (-0.39 to -0.17) | -0.84 (-0.93 to -0.75) |
| North Macedonia | 0.4 (0.3 to 0.6) | 19.81 (12.46 to 29.74) | 0.6 (0.4 to 0.9) | 21.42 (13.42 to 31.97) | 0.42 (0.28 to 0.59) | 0.25 (0.2 to 0.3) |
| Northern Mariana Islands | 0 (0 to 0) | 22.4 (14.98 to 32.47) | 0 (0 to 0) | 20.83 (13.21 to 30.83) | 0.37 (0.1 to 0.75) | -0.39 (-0.51 to -0.27) |
| Norway | 1.4 (1 to 2) | 28.2 (19.61 to 40.14) | 1.7 (1.1 to 2.5) | 24.42 (16.03 to 36.52) | 0.18 (0.09 to 0.27) | -0.84 (-1.04 to -0.64) |
| Oman | 0.3 (0.2 to 0.4) | 18.12 (11.88 to 26.91) | 0.6 (0.4 to 1) | 15.27 (9.45 to 22.89) | 1.26 (0.94 to 1.63) | -0.67 (-0.73 to -0.61) |
| Pakistan | 29.3 (20 to 42.4) | 35.32 (24.47 to 51.04) | 58.3 (38.1 to 84.9) | 32.61 (21.87 to 46.51) | 0.99 (0.74 to 1.2) | -0.27 (-0.38 to -0.16) |
| Palau | 0 (0 to 0) | 36.17 (24.62 to 49.39) | 0 (0 to 0) | 31.06 (21.53 to 43.02) | 0.6 (0.28 to 0.96) | -0.53 (-0.59 to -0.47) |
| Palestine | 0.3 (0.2 to 0.4) | 18.17 (11.64 to 26.78) | 0.7 (0.5 to 1.1) | 17.74 (11.52 to 26.49) | 1.64 (1.32 to 2.01) | 0.05 (-0.03 to 0.13) |
| Panama | 0.4 (0.3 to 0.6) | 21.47 (15.86 to 29.03) | 0.7 (0.5 to 0.9) | 15.56 (10.97 to 21.85) | 0.59 (0.39 to 0.79) | -1.03 (-1.15 to -0.9) |
| Papua New Guinea | 1.1 (0.7 to 1.6) | 33.45 (22.64 to 48.67) | 2.5 (1.6 to 3.7) | 30.74 (19.93 to 44.75) | 1.32 (1.04 to 1.57) | -0.31 (-0.39 to -0.23) |
| Paraguay | 1.3 (0.8 to 2) | 42.57 (27.24 to 63.38) | 1.9 (1.3 to 2.8) | 29.74 (19.53 to 43.52) | 0.45 (0.31 to 0.61) | -1.17 (-1.35 to -0.99) |
| Peru | 9.7 (7 to 13.1) | 57.99 (42.72 to 77.04) | 19 (14 to 25.5) | 57.27 (42.18 to 76.33) | 0.96 (0.49 to 1.48) | 0.58 (0.29 to 0.87) |
| Philippines | 24.3 (18.2 to 32) | 47.25 (37.43 to 60.31) | 32.2 (22.9 to 44.5) | 31.72 (22.83 to 43.3) | 0.32 (0.12 to 0.57) | -1.5 (-1.65 to -1.36) |
| Poland | 19.9 (13 to 29.5) | 49.28 (32.15 to 73.37) | 21.8 (15 to 30.2) | 41.72 (28.58 to 58.3) | 0.09 (-0.03 to 0.25) | -0.39 (-0.48 to -0.3) |
| Portugal | 2.3 (1.6 to 3.2) | 19.17 (13.1 to 27.06) | 2.6 (1.7 to 3.8) | 16.65 (10.57 to 24.84) | 0.15 (0 to 0.29) | -0.51 (-0.64 to -0.37) |
| Puerto Rico | 1 (0.7 to 1.4) | 26.99 (18.7 to 38.56) | 1.3 (0.8 to 1.9) | 26.63 (16.85 to 40.07) | 0.32 (0.13 to 0.51) | -0.13 (-0.23 to -0.03) |
| Qatar | 0.1 (0 to 0.1) | 14.08 (9.21 to 20.51) | 0.4 (0.2 to 0.6) | 16.03 (10.8 to 23.07) | 6.52 (5.38 to 7.84) | 0.96 (0.79 to 1.14) |
| Republic of Korea | 13.3 (9 to 16.5) | 47.89 (27.36 to 58.67) | 5.5 (3.7 to 7.8) | 8.66 (5.83 to 12.44) | -0.59 (-0.71 to -0.36) | -7.9 (-8.83 to -6.95) |
| Republic of Moldova | 0.9 (0.6 to 1.3) | 18.96 (12.35 to 27.8) | 0.9 (0.6 to 1.4) | 19.86 (12.48 to 29.56) | 0.07 (-0.05 to 0.2) | 0.27 (0.19 to 0.36) |
| Romania | 7.6 (5.4 to 10.4) | 29.52 (21 to 40.65) | 8.2 (6 to 11.2) | 30.33 (21.61 to 42.05) | 0.08 (-0.06 to 0.22) | 0.34 (0.18 to 0.49) |
| Russian Federation | 37.2 (26.9 to 51.4) | 22.16 (15.94 to 30.69) | 44.1 (31.3 to 60.9) | 23.67 (16.96 to 33.04) | 0.19 (0.12 to 0.26) | 0.16 (0.04 to 0.28) |
| Rwanda | 5.7 (3.7 to 8.1) | 137.89 (87.17 to 203.06) | 7.1 (4.9 to 10) | 87.49 (60.57 to 121.61) | 0.25 (-0.1 to 0.74) | -2.25 (-2.53 to -1.97) |
| Saint Kitts and Nevis | 0 (0 to 0) | 54.98 (41.75 to 69.77) | 0 (0 to 0) | 43.79 (31.22 to 58.98) | 0.61 (0.32 to 0.93) | -0.96 (-1.08 to -0.85) |
| Saint Lucia | 0 (0 to 0.1) | 41.33 (30.78 to 54.19) | 0.1 (0.1 to 0.1) | 37.41 (26.34 to 51.38) | 0.8 (0.53 to 1.08) | -0.34 (-0.45 to -0.23) |
| Saint Vincent and the Grenadines | 0.1 (0.1 to 0.1) | 81.55 (64.78 to 102.6) | 0.1 (0.1 to 0.1) | 78.45 (61.58 to 99.24) | 0.57 (0.31 to 0.84) | -0.56 (-0.71 to -0.42) |
| Samoa | 0 (0 to 0) | 26.68 (17.61 to 37.55) | 0 (0 to 0.1) | 22.47 (14.68 to 32.41) | 0.27 (0.08 to 0.43) | -0.71 (-0.8 to -0.62) |
| San Marino | 0 (0 to 0) | 16.09 (10.44 to 23.74) | 0 (0 to 0) | 16.73 (10.66 to 24.79) | 0.69 (0.52 to 0.87) | 0.22 (0.18 to 0.25) |
| Sao Tome and Principe | 0 (0 to 0.1) | 37.39 (24.76 to 55.64) | 0 (0 to 0.1) | 22.41 (15.25 to 32.24) | 0.05 (-0.35 to 0.53) | -2.1 (-2.29 to -1.91) |
| Saudi Arabia | 1.9 (1.2 to 2.9) | 15.7 (10.23 to 22.89) | 4.9 (3 to 7.4) | 13.88 (8.87 to 20.43) | 1.53 (1.15 to 1.95) | -0.32 (-0.4 to -0.24) |
| Senegal | 3.3 (1.9 to 5) | 54.26 (33.81 to 74.71) | 4.2 (2.7 to 5.8) | 36.52 (24.12 to 50.82) | 0.27 (-0.17 to 0.89) | -1.5 (-1.66 to -1.33) |
| Serbia | 1.8 (1.2 to 2.5) | 16.76 (11.33 to 23.65) | 2.8 (2 to 3.9) | 23.11 (16.25 to 32.48) | 0.6 (0.32 to 0.85) | 1.95 (1.68 to 2.23) |
| Seychelles | 0 (0 to 0) | 35.56 (25.46 to 47.49) | 0 (0 to 0) | 28.27 (19.5 to 38.93) | 0.46 (0.21 to 0.72) | -0.86 (-0.93 to -0.79) |
| Sierra Leone | 1.7 (1 to 2.9) | 50.25 (32.52 to 77.02) | 2.8 (1.9 to 4) | 42.77 (29.31 to 58.52) | 0.63 (-0.09 to 1.79) | -0.38 (-0.58 to -0.19) |
| Singapore | 0.3 (0.2 to 0.4) | 9.06 (6.3 to 12.84) | 0.5 (0.3 to 0.7) | 7.43 (4.74 to 11.24) | 0.74 (0.49 to 1.02) | -0.66 (-0.74 to -0.59) |
| Slovakia | 1.3 (0.9 to 1.8) | 23.28 (15.83 to 32.94) | 1.9 (1.4 to 2.8) | 26.76 (18.54 to 37.76) | 0.5 (0.25 to 0.73) | 0.76 (0.59 to 0.92) |
| Slovenia | 0.5 (0.3 to 0.7) | 22.37 (14.72 to 32.52) | 0.7 (0.5 to 1) | 24.19 (15.55 to 35.36) | 0.44 (0.29 to 0.59) | 0.53 (0.05 to 1.01) |
| Solomon Islands | 0.1 (0.1 to 0.1) | 34.08 (23.32 to 48.08) | 0.1 (0.1 to 0.2) | 28.21 (18.82 to 40.26) | 0.76 (0.51 to 1) | -0.73 (-0.82 to -0.65) |
| Somalia | 4.9 (2.8 to 8) | 120.11 (69.27 to 198.78) | 11.8 (7 to 19.7) | 108.6 (64.8 to 178.44) | 1.42 (0.83 to 2.14) | -0.45 (-0.57 to -0.34) |
| South Africa | 25.5 (18.5 to 31.9) | 82.99 (61.76 to 102.62) | 37.3 (30 to 46.7) | 70.63 (57.09 to 87.57) | 0.46 (0.18 to 0.89) | -0.21 (-0.88 to 0.45) |
| South Sudan | 3.2 (2.1 to 4.7) | 92.39 (58.4 to 141.91) | 4.7 (3 to 7) | 84.87 (54.65 to 126.24) | 0.48 (0.1 to 0.91) | -0.3 (-0.33 to -0.28) |
| Spain | 7.5 (4.9 to 10.9) | 16.32 (10.74 to 23.84) | 11 (7 to 16.1) | 16.57 (10.27 to 24.27) | 0.48 (0.34 to 0.62) | 0.14 (0.1 to 0.17) |
| Sri Lanka | 6.9 (5.2 to 8.9) | 50.51 (38.49 to 64.44) | 7 (4.9 to 9.8) | 28.93 (20.42 to 40.36) | 0.02 (-0.16 to 0.18) | -1.6 (-1.74 to -1.46) |
| Sudan | 3.3 (2.1 to 4.9) | 20.93 (13.72 to 31.2) | 7.6 (5 to 11.3) | 23.55 (15.59 to 34.47) | 1.33 (0.94 to 1.68) | 0.57 (0.48 to 0.65) |
| Suriname | 0.1 (0.1 to 0.2) | 44.24 (33.71 to 57.88) | 0.3 (0.2 to 0.4) | 42.8 (31.97 to 57.05) | 0.86 (0.6 to 1.15) | -0.21 (-0.34 to -0.07) |
| Sweden | 2.1 (1.5 to 3) | 18.9 (12.91 to 26.85) | 1.7 (1.2 to 2.5) | 12.92 (8.38 to 18.88) | -0.17 (-0.25 to -0.09) | -1.69 (-1.83 to -1.55) |
| Switzerland | 1.3 (0.9 to 1.8) | 14.86 (10.04 to 21.31) | 2 (1.3 to 2.8) | 15.27 (10.12 to 22.05) | 0.53 (0.38 to 0.68) | 0.08 (-0.21 to 0.36) |
| Syrian Arab Republic | 1.7 (1.1 to 2.5) | 17.01 (11.28 to 24.8) | 2.3 (1.5 to 3.4) | 16.14 (10.48 to 23.58) | 0.36 (0.16 to 0.61) | -0.19 (-0.26 to -0.12) |
| Taiwan (Province of China) | 6.3 (4.3 to 8.8) | 36.6 (26.11 to 50.71) | 8.8 (5.2 to 13.3) | 25.45 (15.61 to 38.2) | 0.41 (0.16 to 0.69) | -1.18 (-1.42 to -0.94) |
| Tajikistan | 0.9 (0.6 to 1.2) | 20.69 (14.66 to 28.29) | 1.7 (1.2 to 2.4) | 19.91 (13.69 to 28.11) | 0.94 (0.65 to 1.25) | -0.5 (-0.66 to -0.35) |
| Thailand | 13.6 (8.9 to 20) | 27.16 (18.03 to 39.27) | 23.5 (15.9 to 33.7) | 26.34 (17.99 to 37.7) | 0.73 (0.49 to 1.01) | 0.1 (0 to 0.2) |
| Timor-Leste | 0.3 (0.2 to 0.5) | 56.44 (35.34 to 81.53) | 0.4 (0.3 to 0.7) | 42.77 (26.93 to 66.2) | 0.37 (-0.07 to 0.85) | -1.08 (-1.19 to -0.97) |
| Togo | 1.4 (0.9 to 2) | 51.1 (34.56 to 66.65) | 2.7 (1.8 to 3.7) | 43.63 (30.48 to 60.36) | 0.96 (0.25 to 1.81) | -0.44 (-0.57 to -0.32) |
| Tokelau | 0 (0 to 0) | 32.17 (21.33 to 44.99) | 0 (0 to 0) | 24.06 (15.94 to 34.37) | -0.28 (-0.4 to -0.15) | -1.12 (-1.22 to -1.01) |
| Tonga | 0 (0 to 0) | 34.5 (23.82 to 48.27) | 0 (0 to 0) | 26.24 (17.28 to 38.06) | -0.05 (-0.17 to 0.08) | -1.09 (-1.21 to -0.97) |
| Trinidad and Tobago | 1 (0.8 to 1.2) | 107.39 (82.75 to 128.78) | 1.3 (1 to 1.7) | 73.85 (55.33 to 95.5) | 0.24 (0.01 to 0.54) | -1.88 (-2.23 to -1.54) |
| Tunisia | 1.1 (0.7 to 1.7) | 15.85 (10.21 to 23.1) | 2 (1.3 to 3) | 16.06 (10.5 to 23.71) | 0.79 (0.54 to 1.06) | 0.14 (0.08 to 0.21) |
| Turkey | 8.8 (5.5 to 13.4) | 16.86 (10.71 to 24.73) | 14.2 (9 to 21.3) | 16 (10.14 to 23.7) | 0.61 (0.41 to 0.84) | -0.07 (-0.15 to 0) |
| Turkmenistan | 0.5 (0.3 to 0.7) | 16.38 (10.92 to 23.21) | 0.8 (0.5 to 1.2) | 16.16 (10.65 to 23.59) | 0.6 (0.4 to 0.83) | -0.01 (-0.08 to 0.06) |
| Tuvalu | 0 (0 to 0) | 36.09 (24.22 to 50.08) | 0 (0 to 0) | 26.14 (17.33 to 36.77) | 0.01 (-0.16 to 0.16) | -1.15 (-1.27 to -1.04) |
| Uganda | 8.6 (5.4 to 12.1) | 93.92 (57.76 to 133.5) | 17.4 (11.8 to 24.1) | 81.52 (56.07 to 111.76) | 1.02 (0.52 to 1.62) | -0.94 (-1.15 to -0.73) |
| Ukraine | 12 (8.2 to 17.2) | 19.65 (13.29 to 28.2) | 12.9 (8.9 to 18.5) | 22.68 (15.47 to 32.84) | 0.07 (-0.04 to 0.21) | 0.48 (0.37 to 0.59) |
| United Arab Emirates | 0.2 (0.1 to 0.3) | 13.52 (8.53 to 20) | 1.5 (0.9 to 2.3) | 13.93 (8.9 to 20.7) | 5.62 (4.43 to 6.87) | 0.13 (0.07 to 0.19) |
| United Kingdom | 15.1 (10.1 to 21.6) | 21.55 (14.53 to 30.98) | 22.3 (15.8 to 30.7) | 24.35 (17 to 33.93) | 0.47 (0.32 to 0.58) | 0.71 (0.59 to 0.84) |
| United Republic of Tanzania | 10 (7.1 to 13.2) | 62.54 (43.93 to 82.77) | 20 (14 to 27.1) | 56 (39.09 to 76.02) | 1.01 (0.53 to 1.48) | -0.44 (-0.6 to -0.27) |
| United States of America | 84.5 (56.6 to 121.6) | 29.66 (19.78 to 42.96) | 72.5 (49.2 to 100.8) | 17.32 (11.58 to 24.36) | -0.14 (-0.23 to -0.03) | -1.91 (-2.1 to -1.72) |
| United States Virgin Islands | 0 (0 to 0) | 30.86 (22.11 to 42.4) | 0 (0 to 0.1) | 32.2 (22.27 to 45.73) | 0.48 (0.28 to 0.7) | 0.21 (0.15 to 0.27) |
| Uruguay | 0.9 (0.6 to 1.1) | 24.98 (18.5 to 32.94) | 0.7 (0.5 to 1) | 17.85 (11.96 to 25.36) | -0.15 (-0.28 to 0.02) | -1.48 (-1.63 to -1.33) |
| Uzbekistan | 2.5 (1.6 to 3.7) | 14.33 (9.15 to 21.23) | 5 (3.1 to 7.5) | 15.53 (9.67 to 23.18) | 1.01 (0.75 to 1.28) | 0.43 (0.35 to 0.51) |
| Vanuatu | 0 (0 to 0.1) | 38.21 (26.73 to 53.61) | 0.1 (0 to 0.1) | 31.46 (21.33 to 44.93) | 0.86 (0.62 to 1.12) | -0.88 (-0.97 to -0.78) |
| Venezuela (Bolivarian Republic of) | 4.7 (3.7 to 5.8) | 35.89 (29.03 to 44.02) | 7.6 (5.7 to 10) | 26.1 (19.52 to 34.02) | 0.64 (0.32 to 0.99) | -1.4 (-1.51 to -1.29) |
| Viet Nam | 13.2 (8.7 to 19.5) | 24.05 (16.16 to 34.93) | 21.1 (13.3 to 32.3) | 20.44 (13.03 to 30.85) | 0.59 (0.36 to 0.84) | -0.57 (-0.64 to -0.49) |
| Yemen | 2.4 (1.5 to 3.5) | 25.19 (16.84 to 35.8) | 6.5 (4.3 to 9.5) | 26.92 (18.36 to 38.06) | 1.7 (1.31 to 2.06) | 0.3 (0.23 to 0.37) |
| Zambia | 4.3 (3.1 to 5.7) | 94.68 (65.46 to 126.07) | 7.8 (5.7 to 10.6) | 72.38 (52.45 to 96.29) | 0.82 (0.33 to 1.43) | -1.35 (-1.63 to -1.07) |
| Zimbabwe | 4.2 (3.1 to 5.4) | 68.71 (52.02 to 86.7) | 8.3 (5.6 to 11.6) | 80.27 (53.22 to 112.23) | 0.98 (0.46 to 1.65) | 0.9 (0.71 to 1.1) |

**Supplementary table 7: Deaths of gastritis and duodenitis in 1990 and 2019 for GBD region, with EAPC from 1990 and 2019.**

| location | Num_1990(x10^3^) | ASR_1990/10^5^ | Num_2019(x10^3^) | ASR_2019/10^5^ | Num_change | EAPC_CI |
| --- | --- | --- | --- | --- | --- | --- |
| **Global** | 32 (25 to 40) | 0.83 (0.67 to 1.07) | 37 (31 to 42) | 0.48 (0.39 to 0.54) | 0.19 (-0.1 to 0.55) | -1.82 (-1.93 to -1.72) |
| **SDI region** |  |  |  |  |  |  |
| High SDI | 2 (2 to 3) | 0.24 (0.2 to 0.26) | 2 (2 to 3) | 0.09 (0.08 to 0.12) | -0.19 (-0.29 to 0.24) | -3.71 (-4.04 to -3.39) |
| High-middle SDI | 6 (4 to 8) | 0.6 (0.46 to 0.81) | 6 (5 to 7) | 0.3 (0.26 to 0.34) | 0.02 (-0.29 to 0.43) | -2.31 (-2.54 to -2.08) |
| Middle SDI | 12 (9 to 16) | 1.4 (1.09 to 1.84) | 15 (12 to 18) | 0.75 (0.58 to 0.86) | 0.27 (-0.16 to 0.78) | -1.82 (-2.02 to -1.62) |
| Low-middle SDI | 7 (5 to 10) | 1.27 (0.94 to 1.84) | 9 (8 to 11) | 0.75 (0.63 to 0.88) | 0.27 (-0.05 to 0.74) | -1.74 (-1.86 to -1.62) |
| Low SDI | 4 (2 to 6) | 1.49 (0.88 to 2.17) | 5 (3 to 7) | 0.91 (0.57 to 1.18) | 0.28 (0.02 to 0.65) | -1.88 (-1.99 to -1.76) |
| **GBD region** |  |  |  |  |  |  |
| Andean Latin America | 0 (0 to 0) | 0.89 (0.65 to 1.39) | 1 (0 to 1) | 1.07 (0.79 to 1.39) | 2.2 (0.86 to 3.9) | 1.8 (1.3 to 2.31) |
| Australasia | 0 (0 to 0) | 0.13 (0.11 to 0.14) | 0 (0 to 0) | 0.07 (0.06 to 0.09) | 0.45 (0.17 to 0.79) | -1.93 (-2.06 to -1.8) |
| Caribbean | 0 (0 to 0) | 0.83 (0.65 to 1.05) | 0 (0 to 0) | 0.62 (0.49 to 0.77) | 0.49 (0.21 to 0.79) | -1.13 (-1.24 to -1.01) |
| Central Asia | 0 (0 to 0) | 0.12 (0.08 to 0.17) | 0 (0 to 0) | 0.1 (0.09 to 0.12) | 0.19 (-0.17 to 0.51) | -0.6 (-0.83 to -0.38) |
| Central Europe | 0 (0 to 0) | 0.19 (0.17 to 0.27) | 1 (0 to 1) | 0.24 (0.2 to 0.27) | 1.02 (0.13 to 1.41) | 1.25 (1 to 1.49) |
| Central Latin America | 1 (1 to 1) | 1.35 (1.22 to 1.45) | 2 (2 to 2) | 0.91 (0.77 to 1.06) | 0.98 (0.74 to 1.27) | -1.72 (-1.92 to -1.52) |
| Central Sub-Saharan Africa | 1 (0 to 1) | 3.07 (1.68 to 4.43) | 1 (1 to 2) | 2.24 (1.18 to 3.26) | 0.63 (0.16 to 1.27) | -1.04 (-1.18 to -0.91) |
| East Asia | 16 (12 to 23) | 2.36 (1.72 to 3.31) | 19 (14 to 22) | 1.08 (0.8 to 1.26) | 0.15 (-0.31 to 0.75) | -2.32 (-2.56 to -2.09) |
| Eastern Europe | 0 (0 to 1) | 0.17 (0.15 to 0.21) | 1 (0 to 1) | 0.18 (0.15 to 0.22) | 0.28 (0.06 to 0.49) | -0.26 (-0.58 to 0.06) |
| Eastern Sub-Saharan Africa | 2 (1 to 3) | 2.69 (1.38 to 3.98) | 3 (2 to 4) | 1.71 (0.89 to 2.3) | 0.35 (0.05 to 0.73) | -1.75 (-1.87 to -1.62) |
| High-income Asia Pacific | 1 (1 to 1) | 0.63 (0.35 to 0.72) | 0 (0 to 1) | 0.04 (0.03 to 0.08) | -0.73 (-0.81 to 0.06) | -11.42 (-12.46 to -10.37) |
| High-income North America | 1 (1 to 1) | 0.2 (0.16 to 0.21) | 0 (0 to 1) | 0.08 (0.07 to 0.1) | -0.3 (-0.37 to 0.16) | -3.68 (-4.01 to -3.36) |
| North Africa and Middle East | 0 (0 to 0) | 0.1 (0.07 to 0.15) | 0 (0 to 0) | 0.11 (0.08 to 0.13) | 1.73 (0.38 to 3.14) | 1.02 (0.74 to 1.31) |
| Oceania | 0 (0 to 0) | 0.31 (0.2 to 0.46) | 0 (0 to 0) | 0.16 (0.11 to 0.24) | 0.23 (-0.11 to 0.75) | -2.72 (-2.94 to -2.5) |
| South Asia | 4 (2 to 7) | 0.77 (0.44 to 1.31) | 4 (3 to 5) | 0.31 (0.21 to 0.43) | -0.08 (-0.33 to 0.38) | -3.72 (-4 to -3.43) |
| Southeast Asia | 1 (1 to 1) | 0.37 (0.28 to 0.47) | 1 (1 to 1) | 0.21 (0.17 to 0.25) | 0.11 (-0.16 to 0.52) | -2.08 (-2.14 to -2.02) |
| Southern Latin America | 0 (0 to 0) | 0.4 (0.3 to 0.49) | 0 (0 to 0) | 0.15 (0.13 to 0.18) | -0.25 (-0.41 to 0.07) | -3.88 (-4.27 to -3.49) |
| Southern Sub-Saharan Africa | 1 (0 to 1) | 1.65 (1.25 to 2) | 1 (1 to 1) | 1.56 (1.3 to 1.75) | 0.64 (0.26 to 1.28) | 0.26 (-0.42 to 0.94) |
| Tropical Latin America | 0 (0 to 0) | 0.42 (0.38 to 0.46) | 1 (1 to 1) | 0.29 (0.25 to 0.32) | 0.93 (0.7 to 1.18) | -1.49 (-1.69 to -1.3) |
| Western Europe | 1 (1 to 1) | 0.15 (0.13 to 0.16) | 1 (1 to 1) | 0.12 (0.1 to 0.13) | 0.49 (0.3 to 0.7) | -0.58 (-0.66 to -0.5) |
| Western Sub-Saharan Africa | 1 (1 to 2) | 0.97 (0.51 to 1.58) | 2 (1 to 2) | 0.58 (0.34 to 0.85) | 0.28 (-0.09 to 0.75) | -1.64 (-1.69 to -1.59) |

**Supplementary table 8:** **Deaths of gastritis and duodenitis in 1990 and 2019 for all locations, with EAPC from 1990 and 2019.**

| location | Num_1990(x10^3^) | ASR_1990/10^5^ | Num_2019(x10^3^) | ASR_2019/10^5^ | Num_change | EAPC_CI |
| --- | --- | --- | --- | --- | --- | --- |
| Afghanistan | 0 (0 to 0) | 0.26 (0.11 to 0.58) | 0 (0 to 0.1) | 0.34 (0.18 to 0.59) | 1.42 (0.27 to 3.37) | 1.43 (1.1 to 1.76) |
| Albania | 0 (0 to 0) | 0.07 (0.06 to 0.1) | 0 (0 to 0) | 0.04 (0.02 to 0.05) | -0.03 (-0.43 to 0.55) | -2.89 (-3.25 to -2.53) |
| Algeria | 0 (0 to 0) | 0.11 (0.05 to 0.19) | 0 (0 to 0) | 0.11 (0.07 to 0.16) | 2.12 (0.45 to 5.35) | 0.56 (0.33 to 0.79) |
| American Samoa | 0 (0 to 0) | 0.58 (0.4 to 0.81) | 0 (0 to 0) | 0.25 (0.17 to 0.36) | 0.04 (-0.36 to 0.81) | -4.16 (-4.99 to -3.34) |
| Andorra | 0 (0 to 0) | 0.05 (0.03 to 0.08) | 0 (0 to 0) | 0.05 (0.03 to 0.08) | 1.91 (0.74 to 3.69) | -0.53 (-0.65 to -0.4) |
| Angola | 0.1 (0.1 to 0.2) | 3.18 (1.67 to 5.02) | 0.2 (0.1 to 0.3) | 2.24 (1.1 to 3.56) | 0.67 (-0.08 to 2.18) | -1.22 (-1.35 to -1.1) |
| Antigua and Barbuda | 0 (0 to 0) | 0.79 (0.63 to 1.01) | 0 (0 to 0) | 0.67 (0.52 to 0.84) | 0.37 (-0.01 to 0.88) | -0.56 (-0.76 to -0.35) |
| Argentina | 0.1 (0.1 to 0.1) | 0.31 (0.23 to 0.42) | 0.1 (0.1 to 0.1) | 0.15 (0.12 to 0.18) | -0.13 (-0.4 to 0.21) | -2.95 (-3.4 to -2.5) |
| Armenia | 0 (0 to 0) | 0.15 (0.1 to 0.24) | 0 (0 to 0) | 0.25 (0.2 to 0.32) | 1.65 (0.57 to 2.79) | 2.65 (2.14 to 3.16) |
| Australia | 0 (0 to 0) | 0.12 (0.1 to 0.14) | 0 (0 to 0) | 0.06 (0.05 to 0.09) | 0.38 (0.09 to 0.76) | -2.09 (-2.23 to -1.95) |
| Austria | 0 (0 to 0) | 0.12 (0.11 to 0.14) | 0 (0 to 0) | 0.1 (0.08 to 0.13) | 0.51 (0.06 to 0.91) | 1.7 (0.66 to 2.74) |
| Azerbaijan | 0 (0 to 0) | 0.08 (0.05 to 0.12) | 0 (0 to 0) | 0.04 (0.03 to 0.07) | -0.18 (-0.5 to 0.38) | -3.14 (-3.7 to -2.57) |
| Bahamas | 0 (0 to 0) | 0.69 (0.54 to 0.92) | 0 (0 to 0) | 0.49 (0.38 to 0.65) | 0.71 (0.17 to 1.47) | -1.29 (-1.43 to -1.15) |
| Bahrain | 0 (0 to 0) | 0.47 (0.29 to 0.74) | 0 (0 to 0) | 0.21 (0.15 to 0.31) | 0.69 (-0.02 to 2.18) | -4.73 (-5.83 to -3.61) |
| Bangladesh | 0.4 (0.2 to 0.7) | 0.76 (0.4 to 1.54) | 0.1 (0.1 to 0.2) | 0.08 (0.04 to 0.14) | -0.73 (-0.85 to -0.43) | -9.27 (-9.9 to -8.64) |
| Barbados | 0 (0 to 0) | 0.32 (0.26 to 0.4) | 0 (0 to 0) | 0.26 (0.19 to 0.34) | 0.32 (-0.06 to 0.82) | -0.98 (-1.16 to -0.81) |
| Belarus | 0 (0 to 0) | 0.11 (0.07 to 0.18) | 0 (0 to 0) | 0.08 (0.05 to 0.12) | -0.14 (-0.47 to 0.3) | -1.66 (-2.07 to -1.23) |
| Belgium | 0 (0 to 0) | 0.06 (0.05 to 0.08) | 0 (0 to 0) | 0.06 (0.05 to 0.07) | 0.9 (0.32 to 1.45) | 0.1 (-0.32 to 0.53) |
| Belize | 0 (0 to 0) | 0.86 (0.69 to 1.11) | 0 (0 to 0) | 0.74 (0.58 to 0.92) | 1.41 (0.78 to 2.23) | -0.65 (-1.07 to -0.22) |
| Benin | 0 (0 to 0) | 0.93 (0.5 to 1.39) | 0 (0 to 0.1) | 0.62 (0.38 to 0.9) | 0.53 (-0.13 to 1.69) | -1.36 (-1.51 to -1.22) |
| Bermuda | 0 (0 to 0) | 0.47 (0.33 to 0.61) | 0 (0 to 0) | 0.23 (0.17 to 0.3) | 0.11 (-0.24 to 0.7) | -2.49 (-2.68 to -2.3) |
| Bhutan | 0 (0 to 0) | 0.87 (0.43 to 1.63) | 0 (0 to 0) | 0.41 (0.19 to 0.77) | -0.02 (-0.45 to 0.74) | -2.66 (-2.72 to -2.59) |
| Bolivia (Plurinational State of) | 0 (0 to 0.1) | 1.33 (0.67 to 2.27) | 0.1 (0.1 to 0.1) | 1.36 (0.92 to 1.95) | 1.54 (0.57 to 3.57) | 0.21 (0.12 to 0.31) |
| Bosnia and Herzegovina | 0 (0 to 0) | 0.22 (0.11 to 0.3) | 0 (0 to 0) | 0.11 (0.04 to 0.16) | -0.05 (-0.51 to 0.5) | -2.65 (-3.1 to -2.2) |
| Botswana | 0 (0 to 0) | 2.29 (1.44 to 3.56) | 0 (0 to 0) | 1.37 (0.91 to 2.03) | 0.39 (-0.17 to 1.28) | -2.4 (-2.89 to -1.9) |
| Brazil | 0.3 (0.3 to 0.4) | 0.43 (0.39 to 0.47) | 0.7 (0.6 to 0.7) | 0.29 (0.25 to 0.33) | 0.92 (0.69 to 1.17) | -1.54 (-1.73 to -1.35) |
| Brunei Darussalam | 0 (0 to 0) | 0.73 (0.4 to 1.17) | 0 (0 to 0) | 0.22 (0.15 to 0.31) | -0.26 (-0.55 to 0.35) | -5.61 (-6.27 to -4.95) |
| Bulgaria | 0 (0 to 0) | 0.2 (0.17 to 0.24) | 0.1 (0 to 0.1) | 0.49 (0.28 to 0.65) | 2.2 (0.85 to 3.42) | 4.13 (3.63 to 4.63) |
| Burkina Faso | 0 (0 to 0.1) | 0.54 (0.3 to 0.84) | 0.1 (0 to 0.1) | 0.39 (0.19 to 0.72) | 0.6 (-0.29 to 2.22) | -0.97 (-1.3 to -0.64) |
| Burundi | 0.1 (0 to 0.1) | 2.61 (1.16 to 4.52) | 0.1 (0.1 to 0.2) | 2.41 (1.27 to 3.96) | 0.82 (0.02 to 2.26) | -0.51 (-0.74 to -0.29) |
| Cabo Verde | 0 (0 to 0) | 0.54 (0.37 to 0.78) | 0 (0 to 0) | 0.16 (0.11 to 0.22) | -0.48 (-0.69 to -0.15) | -4.06 (-5 to -3.11) |
| Cambodia | 0.1 (0 to 0.1) | 1.82 (0.91 to 2.74) | 0.1 (0 to 0.1) | 0.95 (0.48 to 1.48) | 0.04 (-0.41 to 1) | -2.45 (-2.66 to -2.24) |
| Cameroon | 0 (0 to 0.1) | 0.87 (0.52 to 1.19) | 0.1 (0 to 0.1) | 0.44 (0.24 to 0.69) | 0.41 (-0.31 to 1.49) | -2.53 (-2.74 to -2.33) |
| Canada | 0.1 (0.1 to 0.1) | 0.28 (0.2 to 0.32) | 0.1 (0.1 to 0.1) | 0.11 (0.09 to 0.14) | -0.03 (-0.23 to 0.46) | -3.54 (-3.83 to -3.25) |
| Central African Republic | 0.1 (0 to 0.1) | 4.77 (2.57 to 8.27) | 0.1 (0 to 0.2) | 4.33 (2.1 to 7.65) | 0.61 (-0.02 to 1.46) | -0.35 (-0.44 to -0.25) |
| Chad | 0.1 (0 to 0.1) | 1.3 (0.65 to 2.08) | 0.1 (0 to 0.1) | 0.93 (0.57 to 1.41) | 0.54 (-0.09 to 1.59) | -1.17 (-1.28 to -1.06) |
| Chile | 0.1 (0 to 0.1) | 0.67 (0.54 to 0.76) | 0 (0 to 0.1) | 0.17 (0.13 to 0.24) | -0.35 (-0.49 to 0.1) | -5.38 (-5.74 to -5.01) |
| China | 16.2 (11.5 to 23.2) | 2.42 (1.75 to 3.41) | 18.6 (13.4 to 21.9) | 1.11 (0.82 to 1.3) | 0.14 (-0.32 to 0.76) | -2.31 (-2.55 to -2.07) |
| Colombia | 0.1 (0.1 to 0.1) | 0.53 (0.46 to 0.62) | 0.2 (0.2 to 0.3) | 0.41 (0.3 to 0.54) | 1.72 (0.99 to 2.64) | -0.55 (-1.28 to 0.19) |
| Comoros | 0 (0 to 0) | 3.04 (1.33 to 5.16) | 0 (0 to 0) | 2.04 (1.03 to 3.44) | 0.42 (-0.11 to 1.95) | -1.61 (-1.82 to -1.39) |
| Congo | 0 (0 to 0) | 3.39 (1.9 to 4.77) | 0 (0 to 0.1) | 2.08 (1.04 to 3.15) | 0.36 (-0.13 to 1.04) | -1.58 (-1.91 to -1.26) |
| Cook Islands | 0 (0 to 0) | 0.12 (0.07 to 0.17) | 0 (0 to 0) | 0.04 (0.02 to 0.07) | -0.16 (-0.58 to 0.67) | -4.06 (-4.32 to -3.79) |
| Costa Rica | 0 (0 to 0) | 0.31 (0.19 to 0.39) | 0 (0 to 0) | 0.12 (0.08 to 0.16) | 0.27 (-0.12 to 1.01) | -5.33 (-6.13 to -4.53) |
| Côte d'Ivoire | 0.1 (0 to 0.1) | 0.79 (0.46 to 1.15) | 0.1 (0 to 0.1) | 0.49 (0.3 to 0.72) | 0.42 (-0.23 to 1.41) | -1.73 (-1.95 to -1.5) |
| Croatia | 0 (0 to 0) | 0.06 (0.05 to 0.09) | 0 (0 to 0) | 0.21 (0.14 to 0.28) | 4.99 (1.21 to 7.64) | 6.56 (5.79 to 7.32) |
| Cuba | 0.1 (0.1 to 0.1) | 0.66 (0.51 to 0.79) | 0.1 (0.1 to 0.1) | 0.56 (0.42 to 0.72) | 0.7 (0.26 to 1.26) | -0.9 (-1.07 to -0.73) |
| Cyprus | 0 (0 to 0) | 0.13 (0.06 to 0.24) | 0 (0 to 0) | 0.04 (0.03 to 0.07) | 0.07 (-0.39 to 1.06) | -4.14 (-4.41 to -3.87) |
| Czechia | 0 (0 to 0) | 0.14 (0.11 to 0.23) | 0 (0 to 0) | 0.12 (0.09 to 0.17) | 0.42 (0 to 0.91) | -1.22 (-2.08 to -0.34) |
| Democratic People's Republic of Korea | 0.2 (0.1 to 0.3) | 1.26 (0.63 to 2.05) | 0.3 (0.2 to 0.5) | 1.13 (0.62 to 1.87) | 0.99 (0.23 to 2.19) | -0.21 (-0.48 to 0.07) |
| Democratic Republic of the Congo | 0.5 (0.2 to 0.7) | 2.92 (1.51 to 4.22) | 0.8 (0.4 to 1.2) | 2.17 (1.07 to 3.38) | 0.67 (0.05 to 1.46) | -0.93 (-1.11 to -0.76) |
| Denmark | 0 (0 to 0) | 0.13 (0.1 to 0.16) | 0 (0 to 0) | 0.21 (0.14 to 0.26) | 1.5 (0.72 to 2.33) | 1.5 (1.11 to 1.88) |
| Djibouti | 0 (0 to 0) | 2.21 (1.1 to 3.56) | 0 (0 to 0) | 1.46 (0.69 to 2.47) | 1.44 (0.41 to 3.08) | -1.67 (-1.83 to -1.51) |
| Dominica | 0 (0 to 0) | 1.02 (0.76 to 1.33) | 0 (0 to 0) | 0.62 (0.43 to 0.85) | -0.24 (-0.5 to 0.17) | -1.83 (-2.03 to -1.63) |
| Dominican Republic | 0 (0 to 0) | 0.38 (0.29 to 0.57) | 0 (0 to 0) | 0.33 (0.23 to 0.48) | 1.12 (0.38 to 2.14) | -0.3 (-0.58 to -0.01) |
| Ecuador | 0 (0 to 0) | 0.39 (0.28 to 0.71) | 0.1 (0.1 to 0.1) | 0.72 (0.53 to 0.92) | 3.92 (1.11 to 6.62) | 4.07 (3.14 to 5.02) |
| Egypt | 0 (0 to 0) | 0.06 (0.02 to 0.15) | 0 (0 to 0.1) | 0.07 (0.03 to 0.14) | 1.4 (0.12 to 3.53) | 1.39 (1.05 to 1.73) |
| El Salvador | 0 (0 to 0) | 0.57 (0.39 to 0.74) | 0 (0 to 0) | 0.29 (0.19 to 0.39) | 0.17 (-0.21 to 0.72) | -2.87 (-3.64 to -2.1) |
| Equatorial Guinea | 0 (0 to 0) | 4.01 (1.9 to 7.94) | 0 (0 to 0) | 1.51 (0.71 to 2.41) | -0.21 (-0.59 to 0.68) | -3.88 (-4.08 to -3.68) |
| Eritrea | 0 (0 to 0.1) | 2.84 (1.47 to 4.57) | 0.1 (0 to 0.1) | 2.77 (1.38 to 4.5) | 1.38 (0.46 to 3.08) | -0.07 (-0.21 to 0.07) |
| Estonia | 0 (0 to 0) | 0.09 (0.07 to 0.11) | 0 (0 to 0) | 0.07 (0.05 to 0.1) | 0.16 (-0.2 to 0.64) | -1.15 (-1.72 to -0.57) |
| Eswatini | 0 (0 to 0) | 1.61 (1.11 to 2.38) | 0 (0 to 0) | 1.43 (0.94 to 2.07) | 0.55 (-0.08 to 1.66) | -0.03 (-0.52 to 0.46) |
| Ethiopia | 0.9 (0.5 to 1.4) | 3.96 (1.97 to 6.18) | 0.8 (0.4 to 1.1) | 1.7 (0.77 to 2.59) | -0.17 (-0.5 to 0.4) | -3.32 (-3.51 to -3.14) |
| Fiji | 0 (0 to 0) | 0.42 (0.22 to 0.61) | 0 (0 to 0) | 0.12 (0.09 to 0.17) | -0.45 (-0.67 to 0.21) | -5.77 (-6.56 to -4.98) |
| Finland | 0 (0 to 0) | 0.31 (0.22 to 0.36) | 0 (0 to 0) | 0.19 (0.12 to 0.24) | 0.05 (-0.19 to 0.39) | -1.89 (-2.02 to -1.75) |
| France | 0.1 (0.1 to 0.1) | 0.1 (0.07 to 0.11) | 0 (0 to 0.1) | 0.03 (0.02 to 0.04) | -0.43 (-0.57 to -0.01) | -4.67 (-5.02 to -4.31) |
| Gabon | 0 (0 to 0) | 2.31 (1.25 to 3.61) | 0 (0 to 0) | 1.43 (0.68 to 2.23) | 0.11 (-0.3 to 0.67) | -1.62 (-1.9 to -1.35) |
| Gambia | 0 (0 to 0) | 1.26 (0.58 to 2.12) | 0 (0 to 0) | 0.56 (0.32 to 0.84) | 0.04 (-0.53 to 1.17) | -3.28 (-3.53 to -3.04) |
| Georgia | 0 (0 to 0) | 0.13 (0.1 to 0.17) | 0 (0 to 0) | 0.13 (0.1 to 0.17) | 0.06 (-0.26 to 0.53) | 0.75 (-0.48 to 1.99) |
| Germany | 0.2 (0.1 to 0.2) | 0.15 (0.12 to 0.19) | 0.4 (0.3 to 0.5) | 0.2 (0.16 to 0.24) | 1.43 (0.65 to 2.34) | 1.93 (1.61 to 2.25) |
| Ghana | 0 (0 to 0) | 0.3 (0.19 to 0.45) | 0 (0 to 0.1) | 0.15 (0.09 to 0.22) | 0.2 (-0.35 to 1.18) | -2.75 (-3.06 to -2.45) |
| Greece | 0 (0 to 0) | 0.04 (0.03 to 0.05) | 0 (0 to 0) | 0.02 (0.02 to 0.03) | 0.15 (-0.16 to 0.67) | -1.62 (-2.03 to -1.21) |
| Greenland | 0 (0 to 0) | 0.72 (0.15 to 1.05) | 0 (0 to 0) | 0.15 (0.07 to 0.2) | -0.62 (-0.77 to -0.11) | -7.53 (-8.73 to -6.32) |
| Grenada | 0 (0 to 0) | 1.51 (1.13 to 2.02) | 0 (0 to 0) | 0.96 (0.76 to 1.2) | -0.09 (-0.33 to 0.29) | -1.79 (-2.04 to -1.54) |
| Guam | 0 (0 to 0) | 0.15 (0.1 to 0.23) | 0 (0 to 0) | 0.05 (0.04 to 0.08) | 0.6 (-0.1 to 1.88) | -4.85 (-5.5 to -4.19) |
| Guatemala | 0.1 (0 to 0.1) | 1.59 (1.22 to 2) | 0.1 (0.1 to 0.2) | 1.43 (1.11 to 1.82) | 1.76 (0.94 to 3.09) | -0.61 (-0.78 to -0.44) |
| Guinea | 0 (0 to 0.1) | 0.97 (0.53 to 1.51) | 0 (0 to 0.1) | 0.63 (0.31 to 1.02) | 0.08 (-0.45 to 0.86) | -1.36 (-1.43 to -1.28) |
| Guinea-Bissau | 0 (0 to 0) | 1.54 (0.86 to 2.38) | 0 (0 to 0) | 0.95 (0.59 to 1.35) | 0.01 (-0.43 to 0.75) | -1.5 (-1.58 to -1.42) |
| Guyana | 0 (0 to 0) | 1.16 (0.91 to 1.49) | 0 (0 to 0) | 0.95 (0.68 to 1.23) | 0.23 (-0.19 to 0.77) | -0.5 (-0.77 to -0.24) |
| Haiti | 0.1 (0 to 0.1) | 2.37 (1.3 to 4.03) | 0.1 (0.1 to 0.2) | 1.54 (0.86 to 2.55) | 0.28 (-0.13 to 0.95) | -1.37 (-1.44 to -1.31) |
| Honduras | 0 (0 to 0.1) | 2.35 (1.46 to 3.21) | 0.1 (0.1 to 0.2) | 2.56 (1.54 to 3.78) | 1.65 (0.72 to 2.93) | 0.36 (0.1 to 0.63) |
| Hungary | 0 (0 to 0) | 0.13 (0.11 to 0.28) | 0 (0 to 0) | 0.21 (0.15 to 0.26) | 1.26 (-0.03 to 2.08) | 1.05 (0.19 to 1.92) |
| Iceland | 0 (0 to 0) | 0.16 (0.13 to 0.19) | 0 (0 to 0) | 0.07 (0.06 to 0.1) | -0.02 (-0.27 to 0.4) | -2.13 (-2.41 to -1.86) |
| India | 3.6 (2 to 5.8) | 0.87 (0.5 to 1.44) | 3.5 (2.4 to 5) | 0.34 (0.24 to 0.5) | -0.03 (-0.29 to 0.47) | -3.7 (-4 to -3.39) |
| Indonesia | 0.1 (0.1 to 0.2) | 0.13 (0.07 to 0.21) | 0.1 (0.1 to 0.2) | 0.09 (0.05 to 0.13) | -0.05 (-0.36 to 0.41) | -1.56 (-1.7 to -1.43) |
| Iran (Islamic Republic of) | 0 (0 to 0) | 0.16 (0.11 to 0.23) | 0.1 (0 to 0.1) | 0.17 (0.07 to 0.2) | 2.47 (0.47 to 4.66) | 0.91 (0.15 to 1.69) |
| Iraq | 0 (0 to 0) | 0.1 (0.07 to 0.15) | 0 (0 to 0) | 0.07 (0.05 to 0.09) | 0.73 (0.04 to 1.82) | -0.95 (-1.27 to -0.63) |
| Ireland | 0 (0 to 0) | 0.17 (0.14 to 0.19) | 0 (0 to 0) | 0.14 (0.09 to 0.18) | 0.71 (0.22 to 1.22) | -1.42 (-2.09 to -0.73) |
| Israel | 0 (0 to 0) | 0.09 (0.07 to 0.11) | 0 (0 to 0) | 0.05 (0.04 to 0.06) | 0.5 (0.1 to 1.32) | -3.19 (-3.66 to -2.73) |
| Italy | 0.2 (0.2 to 0.2) | 0.23 (0.2 to 0.24) | 0.2 (0.1 to 0.2) | 0.09 (0.08 to 0.12) | -0.16 (-0.28 to 0.11) | -3.65 (-3.82 to -3.48) |
| Jamaica | 0 (0 to 0) | 0.31 (0.25 to 0.37) | 0 (0 to 0) | 0.47 (0.31 to 0.61) | 1.69 (0.71 to 2.67) | 2.49 (2.1 to 2.88) |
| Japan | 0.5 (0.3 to 0.5) | 0.4 (0.28 to 0.44) | 0.2 (0.1 to 0.3) | 0.03 (0.02 to 0.07) | -0.68 (-0.77 to -0.03) | -9.53 (-10.44 to -8.62) |
| Jordan | 0 (0 to 0) | 0.04 (0.03 to 0.06) | 0 (0 to 0) | 0.02 (0.01 to 0.03) | 0.74 (0.03 to 2.13) | -3.55 (-3.88 to -3.21) |
| Kazakhstan | 0 (0 to 0) | 0.13 (0.05 to 0.3) | 0 (0 to 0) | 0.11 (0.07 to 0.13) | 0.1 (-0.49 to 0.85) | -1.34 (-1.78 to -0.91) |
| Kenya | 0.3 (0.1 to 0.5) | 3.06 (1.44 to 5.81) | 0.5 (0.3 to 0.8) | 2.33 (1.18 to 3.46) | 0.96 (0.36 to 1.82) | -0.79 (-0.9 to -0.67) |
| Kiribati | 0 (0 to 0) | 1 (0.5 to 1.83) | 0 (0 to 0) | 0.5 (0.27 to 0.83) | -0.21 (-0.58 to 0.34) | -2.79 (-2.99 to -2.59) |
| Kuwait | 0 (0 to 0) | 0.03 (0.02 to 0.04) | 0 (0 to 0) | 0.02 (0.01 to 0.03) | 1.46 (0.71 to 2.73) | -1.36 (-1.61 to -1.12) |
| Kyrgyzstan | 0 (0 to 0) | 0.14 (0.1 to 0.19) | 0 (0 to 0) | 0.05 (0.04 to 0.09) | -0.52 (-0.66 to 0) | -4.25 (-5.16 to -3.33) |
| Lao People's Democratic Republic | 0 (0 to 0) | 1.49 (0.76 to 2.43) | 0 (0 to 0.1) | 0.85 (0.4 to 1.4) | 0.08 (-0.38 to 1.22) | -2.13 (-2.24 to -2.02) |
| Latvia | 0 (0 to 0) | 0.23 (0.17 to 0.28) | 0 (0 to 0) | 0.16 (0.12 to 0.22) | -0.23 (-0.45 to 0.1) | -2.65 (-3.59 to -1.7) |
| Lebanon | 0 (0 to 0) | 0.07 (0.04 to 0.11) | 0 (0 to 0) | 0.09 (0.04 to 0.17) | 2.9 (0.5 to 6.12) | 1.96 (1.6 to 2.32) |
| Lesotho | 0 (0 to 0) | 2.13 (1.42 to 2.99) | 0 (0 to 0) | 2.11 (1.31 to 3.05) | 0.3 (-0.28 to 1.2) | 0.37 (0.09 to 0.66) |
| Liberia | 0 (0 to 0) | 0.91 (0.45 to 1.51) | 0 (0 to 0) | 0.53 (0.29 to 0.84) | -0.03 (-0.5 to 0.83) | -1.88 (-2.11 to -1.65) |
| Libya | 0 (0 to 0) | 0.1 (0.05 to 0.18) | 0 (0 to 0) | 0.12 (0.06 to 0.18) | 2.01 (0.51 to 4.5) | 0.98 (0.72 to 1.24) |
| Lithuania | 0 (0 to 0) | 0.06 (0.05 to 0.09) | 0 (0 to 0) | 0.18 (0.13 to 0.23) | 2.67 (1.12 to 4.02) | 4.51 (3.85 to 5.18) |
| Luxembourg | 0 (0 to 0) | 0.13 (0.11 to 0.16) | 0 (0 to 0) | 0.08 (0.06 to 0.11) | 0.35 (0.01 to 0.78) | -1.29 (-1.46 to -1.11) |
| Madagascar | 0.1 (0.1 to 0.2) | 1.85 (0.91 to 3.02) | 0.2 (0.1 to 0.3) | 1.82 (0.82 to 2.75) | 0.95 (0.17 to 2.26) | 0.07 (0.01 to 0.14) |
| Malawi | 0.1 (0 to 0.1) | 2.34 (1.14 to 3.58) | 0.1 (0.1 to 0.2) | 1.55 (0.84 to 2.47) | 0.26 (-0.29 to 1.04) | -1.71 (-1.94 to -1.47) |
| Malaysia | 0 (0 to 0) | 0.11 (0.08 to 0.16) | 0 (0 to 0) | 0.07 (0.05 to 0.1) | 0.69 (0.03 to 1.7) | -2.7 (-3.21 to -2.19) |
| Maldives | 0 (0 to 0) | 0.06 (0.03 to 0.11) | 0 (0 to 0) | 0.02 (0.01 to 0.03) | 0.1 (-0.44 to 1.47) | -4.78 (-5.02 to -4.54) |
| Mali | 0.1 (0 to 0.1) | 1.15 (0.55 to 1.84) | 0.1 (0 to 0.2) | 0.76 (0.4 to 1.25) | 0.4 (-0.35 to 1.75) | -1.29 (-1.54 to -1.04) |
| Malta | 0 (0 to 0) | 0.26 (0.22 to 0.32) | 0 (0 to 0) | 0.17 (0.13 to 0.22) | 0.59 (0.21 to 1.05) | -1.06 (-1.24 to -0.88) |
| Marshall Islands | 0 (0 to 0) | 0.62 (0.37 to 0.91) | 0 (0 to 0) | 0.24 (0.11 to 0.42) | -0.26 (-0.62 to 0.3) | -3.75 (-3.95 to -3.55) |
| Mauritania | 0 (0 to 0) | 1.22 (0.55 to 1.85) | 0 (0 to 0) | 0.45 (0.25 to 0.71) | -0.31 (-0.62 to 0.19) | -3.59 (-3.73 to -3.44) |
| Mauritius | 0 (0 to 0) | 0.43 (0.36 to 0.55) | 0 (0 to 0) | 0.28 (0.21 to 0.36) | 0.56 (0.15 to 1.1) | -1.17 (-1.99 to -0.35) |
| Mexico | 0.7 (0.7 to 0.8) | 1.89 (1.7 to 2.03) | 1.3 (1.1 to 1.5) | 1.23 (1.04 to 1.46) | 0.81 (0.55 to 1.11) | -1.97 (-2.2 to -1.75) |
| Micronesia (Federated States of) | 0 (0 to 0) | 0.65 (0.37 to 1.09) | 0 (0 to 0) | 0.27 (0.13 to 0.48) | -0.42 (-0.72 to 0.09) | -3.32 (-3.67 to -2.97) |
| Monaco | 0 (0 to 0) | 0.04 (0.03 to 0.06) | 0 (0 to 0) | 0.02 (0.01 to 0.03) | -0.19 (-0.54 to 0.36) | -2.27 (-2.48 to -2.07) |
| Mongolia | 0 (0 to 0) | 1.02 (0.54 to 1.65) | 0 (0 to 0) | 0.77 (0.38 to 1.19) | 0.17 (-0.38 to 1.22) | -0.86 (-1.09 to -0.62) |
| Montenegro | 0 (0 to 0) | 0.02 (0.01 to 0.04) | 0 (0 to 0) | 0.02 (0.02 to 0.03) | 0.59 (-0.13 to 1.59) | 0.29 (0.06 to 0.51) |
| Morocco | 0 (0 to 0) | 0.12 (0.06 to 0.23) | 0 (0 to 0) | 0.14 (0.09 to 0.2) | 1.64 (0.41 to 3.37) | 1.09 (0.87 to 1.3) |
| Mozambique | 0.1 (0.1 to 0.2) | 1.82 (0.86 to 2.86) | 0.2 (0.1 to 0.4) | 1.75 (0.83 to 2.94) | 0.9 (0.07 to 2.07) | 0.19 (0.04 to 0.34) |
| Myanmar | 0.2 (0.1 to 0.4) | 0.73 (0.38 to 1.19) | 0.1 (0.1 to 0.2) | 0.31 (0.2 to 0.44) | -0.32 (-0.62 to 0.36) | -3.07 (-3.25 to -2.89) |
| Namibia | 0 (0 to 0) | 2.01 (1.38 to 2.86) | 0 (0 to 0) | 1.22 (0.81 to 1.82) | 0.18 (-0.26 to 0.82) | -1.75 (-2.01 to -1.49) |
| Nauru | 0 (0 to 0) | 0.44 (0.25 to 0.65) | 0 (0 to 0) | 0.26 (0.15 to 0.41) | -0.47 (-0.65 to -0.18) | -2.16 (-2.36 to -1.97) |
| Nepal | 0 (0 to 0) | 0.14 (0.06 to 0.42) | 0 (0 to 0) | 0.08 (0.04 to 0.15) | 0.24 (-0.33 to 1.26) | -2.38 (-2.59 to -2.18) |
| Netherlands | 0 (0 to 0) | 0.15 (0.12 to 0.17) | 0 (0 to 0) | 0.07 (0.05 to 0.11) | -0.15 (-0.36 to 0.3) | -3.63 (-4.06 to -3.19) |
| New Zealand | 0 (0 to 0) | 0.18 (0.15 to 0.2) | 0 (0 to 0) | 0.13 (0.1 to 0.16) | 0.68 (0.33 to 1.05) | -1.37 (-1.68 to -1.06) |
| Nicaragua | 0 (0 to 0) | 1.03 (0.74 to 1.29) | 0 (0 to 0) | 0.71 (0.54 to 0.91) | 0.82 (0.28 to 1.58) | -1.5 (-2.01 to -0.98) |
| Niger | 0.1 (0 to 0.1) | 1.29 (0.65 to 2.15) | 0.1 (0.1 to 0.2) | 0.97 (0.5 to 1.6) | 0.77 (-0.13 to 2.4) | -0.9 (-1.08 to -0.73) |
| Nigeria | 0.6 (0.3 to 1.2) | 1.08 (0.5 to 2.05) | 0.8 (0.4 to 1.4) | 0.61 (0.31 to 1.23) | 0.18 (-0.21 to 0.8) | -1.64 (-1.76 to -1.52) |
| Niue | 0 (0 to 0) | 0.38 (0.21 to 0.59) | 0 (0 to 0) | 0.18 (0.11 to 0.29) | -0.59 (-0.76 to -0.3) | -2.85 (-2.99 to -2.72) |
| North Macedonia | 0 (0 to 0) | 0.05 (0.03 to 0.06) | 0 (0 to 0) | 0.05 (0.03 to 0.07) | 0.56 (0.04 to 1.25) | -0.52 (-1.15 to 0.11) |
| Northern Mariana Islands | 0 (0 to 0) | 0.19 (0.09 to 0.28) | 0 (0 to 0) | 0.05 (0.04 to 0.07) | -0.23 (-0.53 to 0.47) | -5.86 (-6.78 to -4.94) |
| Norway | 0 (0 to 0) | 0.25 (0.2 to 0.28) | 0 (0 to 0) | 0.13 (0.11 to 0.16) | -0.2 (-0.29 to -0.05) | -1.96 (-2.54 to -1.37) |
| Oman | 0 (0 to 0) | 0.06 (0.03 to 0.09) | 0 (0 to 0) | 0.04 (0.02 to 0.05) | 0.18 (-0.39 to 1.21) | -1.04 (-1.49 to -0.58) |
| Pakistan | 0.2 (0.1 to 0.3) | 0.35 (0.15 to 0.69) | 0.2 (0.1 to 0.4) | 0.24 (0.14 to 0.41) | 0.18 (-0.34 to 1.19) | -1.29 (-1.34 to -1.24) |
| Palau | 0 (0 to 0) | 0.7 (0.4 to 1.11) | 0 (0 to 0) | 0.48 (0.29 to 0.71) | 0.41 (-0.31 to 1.8) | -1.32 (-1.41 to -1.23) |
| Palestine | 0 (0 to 0) | 0.08 (0.04 to 0.16) | 0 (0 to 0) | 0.08 (0.05 to 0.11) | 1.21 (0.08 to 3.24) | 0.25 (-0.07 to 0.56) |
| Panama | 0 (0 to 0) | 0.27 (0.22 to 0.32) | 0 (0 to 0) | 0.14 (0.1 to 0.19) | 0.46 (0.04 to 1.03) | -2.02 (-2.4 to -1.64) |
| Papua New Guinea | 0 (0 to 0) | 0.24 (0.12 to 0.4) | 0 (0 to 0) | 0.16 (0.09 to 0.25) | 0.57 (0.03 to 1.5) | -1.6 (-1.71 to -1.49) |
| Paraguay | 0 (0 to 0) | 0.13 (0.07 to 0.19) | 0 (0 to 0) | 0.16 (0.06 to 0.23) | 2.11 (0.9 to 3.76) | 1.62 (1.18 to 2.06) |
| Peru | 0.1 (0.1 to 0.2) | 0.99 (0.74 to 1.51) | 0.4 (0.3 to 0.5) | 1.18 (0.8 to 1.64) | 2.16 (0.77 to 4.08) | 1.89 (1.26 to 2.53) |
| Philippines | 0.3 (0.2 to 0.3) | 0.92 (0.72 to 1.24) | 0.3 (0.2 to 0.4) | 0.38 (0.31 to 0.62) | 0.05 (-0.2 to 0.49) | -3.55 (-3.86 to -3.23) |
| Poland | 0.1 (0.1 to 0.1) | 0.2 (0.18 to 0.36) | 0.2 (0.1 to 0.2) | 0.22 (0.18 to 0.27) | 0.88 (0.11 to 1.35) | 0.42 (0.01 to 0.82) |
| Portugal | 0 (0 to 0) | 0.22 (0.16 to 0.26) | 0 (0 to 0) | 0.06 (0.05 to 0.08) | -0.3 (-0.46 to 0.08) | -5.15 (-5.76 to -4.54) |
| Puerto Rico | 0 (0 to 0) | 0.23 (0.18 to 0.28) | 0 (0 to 0) | 0.05 (0.04 to 0.07) | -0.47 (-0.64 to -0.15) | -5.79 (-6.32 to -5.27) |
| Qatar | 0 (0 to 0) | 0.11 (0.06 to 0.28) | 0 (0 to 0) | 0.18 (0.12 to 0.29) | 7.67 (1.57 to 18.72) | 4.31 (3.34 to 5.29) |
| Republic of Korea | 0.5 (0.2 to 0.6) | 2.73 (1.2 to 3.51) | 0.1 (0.1 to 0.2) | 0.14 (0.1 to 0.22) | -0.77 (-0.86 to -0.06) | -13.54 (-15.05 to -11.99) |
| Republic of Moldova | 0 (0 to 0) | 0.06 (0.04 to 0.07) | 0 (0 to 0) | 0.05 (0.04 to 0.06) | 0.22 (-0.29 to 0.7) | -0.44 (-0.97 to 0.08) |
| Romania | 0.1 (0.1 to 0.1) | 0.28 (0.24 to 0.36) | 0.1 (0.1 to 0.1) | 0.31 (0.24 to 0.39) | 0.56 (0 to 1.1) | 1.1 (0.65 to 1.55) |
| Russian Federation | 0.3 (0.3 to 0.5) | 0.19 (0.17 to 0.27) | 0.4 (0.4 to 0.6) | 0.2 (0.16 to 0.28) | 0.33 (0.12 to 0.54) | -0.32 (-0.7 to 0.06) |
| Rwanda | 0.1 (0.1 to 0.2) | 3.65 (1.76 to 6.17) | 0.1 (0.1 to 0.2) | 1.8 (0.89 to 2.81) | -0.03 (-0.43 to 0.56) | -3.32 (-3.71 to -2.93) |
| Saint Kitts and Nevis | 0 (0 to 0) | 1.04 (0.78 to 1.32) | 0 (0 to 0) | 0.53 (0.39 to 0.72) | -0.09 (-0.39 to 0.33) | -2.51 (-2.8 to -2.22) |
| Saint Lucia | 0 (0 to 0) | 0.7 (0.56 to 0.88) | 0 (0 to 0) | 0.46 (0.35 to 0.59) | 0.65 (0.2 to 1.21) | -1.63 (-1.93 to -1.33) |
| Saint Vincent and the Grenadines | 0 (0 to 0) | 1.81 (1.51 to 2.2) | 0 (0 to 0) | 1.74 (1.35 to 2.14) | 0.72 (0.29 to 1.21) | -0.79 (-1.03 to -0.54) |
| Samoa | 0 (0 to 0) | 0.4 (0.22 to 0.66) | 0 (0 to 0) | 0.21 (0.11 to 0.33) | -0.07 (-0.46 to 0.41) | -2.61 (-2.84 to -2.38) |
| San Marino | 0 (0 to 0) | 0.1 (0.07 to 0.14) | 0 (0 to 0) | 0.07 (0.05 to 0.11) | 0.97 (0.13 to 2.24) | -1.09 (-1.18 to -1) |
| Sao Tome and Principe | 0 (0 to 0) | 0.65 (0.33 to 1.12) | 0 (0 to 0) | 0.21 (0.12 to 0.33) | -0.5 (-0.77 to -0.01) | -4.27 (-4.74 to -3.8) |
| Saudi Arabia | 0 (0 to 0) | 0.08 (0.04 to 0.17) | 0 (0 to 0) | 0.06 (0.03 to 0.09) | 0.86 (-0.31 to 3.88) | -0.73 (-1.07 to -0.39) |
| Senegal | 0 (0 to 0.1) | 0.94 (0.5 to 1.49) | 0 (0 to 0.1) | 0.57 (0.28 to 0.97) | 0.14 (-0.37 to 0.92) | -1.79 (-2 to -1.58) |
| Serbia | 0 (0 to 0) | 0.13 (0.1 to 0.17) | 0 (0 to 0.1) | 0.31 (0.16 to 0.41) | 2.7 (0.45 to 4.54) | 4.97 (4.25 to 5.7) |
| Seychelles | 0 (0 to 0) | 0.57 (0.34 to 0.81) | 0 (0 to 0) | 0.37 (0.21 to 0.54) | 0.1 (-0.25 to 0.64) | -1.57 (-1.68 to -1.46) |
| Sierra Leone | 0 (0 to 0) | 0.82 (0.44 to 1.38) | 0 (0 to 0.1) | 0.62 (0.34 to 0.99) | 0.36 (-0.39 to 1.8) | -0.61 (-0.89 to -0.33) |
| Singapore | 0 (0 to 0) | 0.09 (0.07 to 0.1) | 0 (0 to 0) | 0.02 (0.01 to 0.03) | -0.11 (-0.35 to 0.43) | -5.39 (-5.94 to -4.83) |
| Slovakia | 0 (0 to 0) | 0.19 (0.15 to 0.36) | 0 (0 to 0) | 0.26 (0.18 to 0.35) | 1.09 (0.07 to 2.24) | 2.03 (1.4 to 2.67) |
| Slovenia | 0 (0 to 0) | 0.15 (0.11 to 0.19) | 0 (0 to 0) | 0.15 (0.09 to 0.21) | 1.26 (0.43 to 2.35) | -1.13 (-1.95 to -0.31) |
| Solomon Islands | 0 (0 to 0) | 0.37 (0.19 to 0.64) | 0 (0 to 0) | 0.2 (0.11 to 0.32) | 0.12 (-0.33 to 0.88) | -2.45 (-2.62 to -2.28) |
| Somalia | 0.1 (0 to 0.2) | 2.95 (1.3 to 5.63) | 0.2 (0.1 to 0.4) | 2.46 (1 to 4.87) | 1.18 (0.44 to 2.15) | -0.69 (-0.87 to -0.52) |
| South Africa | 0.4 (0.3 to 0.5) | 1.63 (1.18 to 2.03) | 0.6 (0.5 to 0.7) | 1.54 (1.25 to 1.75) | 0.63 (0.19 to 1.39) | 0.28 (-0.54 to 1.1) |
| South Sudan | 0.1 (0 to 0.1) | 2.21 (1.13 to 3.87) | 0.1 (0 to 0.1) | 1.83 (0.81 to 3.18) | 0.27 (-0.2 to 0.95) | -0.59 (-0.64 to -0.53) |
| Spain | 0 (0 to 0.1) | 0.09 (0.07 to 0.1) | 0.1 (0.1 to 0.1) | 0.06 (0.04 to 0.07) | 0.52 (0.16 to 1.04) | -1.4 (-1.54 to -1.27) |
| Sri Lanka | 0.1 (0.1 to 0.1) | 1.16 (0.76 to 1.45) | 0.1 (0.1 to 0.1) | 0.44 (0.31 to 0.59) | -0.15 (-0.41 to 0.21) | -2.36 (-2.67 to -2.06) |
| Sudan | 0 (0 to 0) | 0.12 (0.04 to 0.27) | 0 (0 to 0.1) | 0.19 (0.08 to 0.33) | 2.08 (0.15 to 7.17) | 2.23 (1.94 to 2.53) |
| Suriname | 0 (0 to 0) | 0.76 (0.59 to 0.99) | 0 (0 to 0) | 0.63 (0.47 to 0.84) | 0.81 (0.29 to 1.52) | -0.72 (-1.08 to -0.36) |
| Sweden | 0 (0 to 0) | 0.17 (0.12 to 0.19) | 0 (0 to 0) | 0.07 (0.06 to 0.09) | -0.35 (-0.47 to -0.08) | -3.56 (-3.77 to -3.35) |
| Switzerland | 0 (0 to 0) | 0.11 (0.09 to 0.14) | 0 (0 to 0) | 0.12 (0.09 to 0.15) | 1.13 (0.52 to 1.98) | 0.46 (0.24 to 0.68) |
| Syrian Arab Republic | 0 (0 to 0) | 0.1 (0.06 to 0.16) | 0 (0 to 0) | 0.07 (0.03 to 0.1) | 0 (-0.44 to 0.83) | -1.41 (-1.53 to -1.29) |
| Taiwan (Province of China) | 0.1 (0 to 0.1) | 0.65 (0.46 to 0.75) | 0 (0 to 0) | 0.04 (0.02 to 0.1) | -0.78 (-0.86 to -0.08) | -11.1 (-11.94 to -10.26) |
| Tajikistan | 0 (0 to 0) | 0.17 (0.09 to 0.25) | 0 (0 to 0) | 0.13 (0.09 to 0.18) | 0.42 (-0.07 to 1.28) | -1.94 (-2.43 to -1.45) |
| Thailand | 0.1 (0 to 0.1) | 0.19 (0.14 to 0.28) | 0.2 (0.2 to 0.3) | 0.23 (0.16 to 0.32) | 2.77 (1.33 to 4.7) | 1.53 (1.05 to 2.02) |
| Timor-Leste | 0 (0 to 0) | 1.3 (0.59 to 2.31) | 0 (0 to 0) | 0.98 (0.4 to 1.9) | 0.75 (-0.03 to 1.87) | -1.15 (-1.25 to -1.06) |
| Togo | 0 (0 to 0) | 0.9 (0.5 to 1.28) | 0 (0 to 0) | 0.67 (0.37 to 1.06) | 0.77 (-0.16 to 2.09) | -0.71 (-0.91 to -0.51) |
| Tokelau | 0 (0 to 0) | 0.42 (0.21 to 0.7) | 0 (0 to 0) | 0.19 (0.12 to 0.29) | -0.56 (-0.73 to -0.23) | -2.98 (-3.24 to -2.72) |
| Tonga | 0 (0 to 0) | 0.56 (0.33 to 0.9) | 0 (0 to 0) | 0.26 (0.15 to 0.41) | -0.14 (-0.49 to 0.47) | -3.01 (-3.24 to -2.78) |
| Trinidad and Tobago | 0 (0 to 0) | 2.59 (1.94 to 3.03) | 0 (0 to 0) | 1.42 (1 to 1.95) | 0.2 (-0.16 to 0.75) | -2.91 (-3.36 to -2.45) |
| Tunisia | 0 (0 to 0) | 0.07 (0.05 to 0.12) | 0 (0 to 0) | 0.09 (0.05 to 0.14) | 2.51 (0.64 to 5.58) | 1.46 (1.18 to 1.74) |
| Turkey | 0 (0 to 0) | 0.05 (0.03 to 0.09) | 0 (0 to 0.1) | 0.05 (0.03 to 0.07) | 1.33 (0.23 to 3.93) | 0.56 (0.13 to 0.99) |
| Turkmenistan | 0 (0 to 0) | 0.09 (0.06 to 0.12) | 0 (0 to 0) | 0.06 (0.04 to 0.09) | 0.39 (-0.09 to 1.13) | -1.32 (-1.56 to -1.08) |
| Tuvalu | 0 (0 to 0) | 0.55 (0.28 to 0.93) | 0 (0 to 0) | 0.23 (0.12 to 0.39) | -0.32 (-0.57 to 0.12) | -3.19 (-3.36 to -3.03) |
| Uganda | 0.2 (0.1 to 0.2) | 2.28 (0.99 to 3.82) | 0.3 (0.1 to 0.4) | 1.69 (0.87 to 2.66) | 0.65 (0.09 to 1.54) | -1.57 (-1.86 to -1.29) |
| Ukraine | 0.1 (0 to 0.1) | 0.13 (0.07 to 0.2) | 0.1 (0.1 to 0.1) | 0.15 (0.08 to 0.19) | 0.16 (-0.24 to 0.64) | -0.04 (-0.36 to 0.27) |
| United Arab Emirates | 0 (0 to 0) | 0.04 (0.02 to 0.09) | 0 (0 to 0) | 0.05 (0.02 to 0.08) | 9.9 (2.83 to 28.34) | 1.05 (0.49 to 1.62) |
| United Kingdom | 0.1 (0.1 to 0.2) | 0.16 (0.13 to 0.24) | 0.3 (0.3 to 0.3) | 0.23 (0.2 to 0.25) | 1.18 (0.54 to 1.65) | 1.53 (1.15 to 1.91) |
| United Republic of Tanzania | 0.1 (0.1 to 0.2) | 1.19 (0.6 to 1.89) | 0.2 (0.1 to 0.3) | 0.84 (0.43 to 1.29) | 0.6 (-0.06 to 1.53) | -1.13 (-1.48 to -0.77) |
| United States of America | 0.6 (0.5 to 0.7) | 0.19 (0.16 to 0.2) | 0.4 (0.3 to 0.6) | 0.07 (0.06 to 0.1) | -0.34 (-0.41 to 0.14) | -3.76 (-4.09 to -3.43) |
| United States Virgin Islands | 0 (0 to 0) | 0.36 (0.26 to 0.47) | 0 (0 to 0) | 0.28 (0.21 to 0.37) | 0.71 (0.15 to 1.61) | -0.5 (-0.72 to -0.28) |
| Uruguay | 0 (0 to 0) | 0.49 (0.3 to 0.57) | 0 (0 to 0) | 0.14 (0.11 to 0.18) | -0.52 (-0.64 to -0.13) | -5.36 (-5.8 to -4.91) |
| Uzbekistan | 0 (0 to 0) | 0.01 (0.01 to 0.03) | 0 (0 to 0) | 0.02 (0.01 to 0.02) | 0.22 (-0.51 to 0.99) | 0.41 (-0.2 to 1.03) |
| Vanuatu | 0 (0 to 0) | 0.57 (0.3 to 1) | 0 (0 to 0) | 0.28 (0.17 to 0.44) | 0.32 (-0.24 to 1.28) | -2.93 (-3.17 to -2.69) |
| Venezuela (Bolivarian Republic of) | 0.1 (0.1 to 0.1) | 0.94 (0.78 to 1.12) | 0.2 (0.1 to 0.3) | 0.65 (0.46 to 0.91) | 1.09 (0.45 to 1.94) | -1.88 (-2.11 to -1.66) |
| Viet Nam | 0.1 (0 to 0.1) | 0.17 (0.08 to 0.3) | 0 (0 to 0.1) | 0.06 (0.04 to 0.1) | -0.17 (-0.61 to 0.7) | -3.84 (-4.1 to -3.58) |
| Yemen | 0 (0 to 0) | 0.19 (0.08 to 0.38) | 0 (0 to 0) | 0.26 (0.15 to 0.4) | 2.58 (0.72 to 6.05) | 1.31 (1.03 to 1.58) |
| Zambia | 0.1 (0 to 0.1) | 2.17 (1.19 to 3.13) | 0.1 (0.1 to 0.1) | 1.27 (0.65 to 1.83) | 0.37 (-0.12 to 1.13) | -2.34 (-2.78 to -1.9) |
| Zimbabwe | 0.1 (0 to 0.1) | 1.51 (1.04 to 2) | 0.1 (0.1 to 0.2) | 1.67 (0.85 to 2.64) | 1 (0.04 to 2.22) | 0.89 (0.67 to 1.12) |

**Supplementary Table 9: Risk factors associated with ASIR and ASDR of GD in 1990.**

| **Risk factors** | ASIR | | ASDR | |
| --- | --- | --- | --- | --- |
|  | r P | | r P | |
| High LDL cholesterol | 0.322 | 0.154 | 0.641** | 0.002 |
| Unsafe sanitation | 0.36 | 0.109 | 0.706** | <0.001 |
| Ambient particulate matter pollution | 0.311 | 0.169 | 0.621** | 0.003 |
| Ambient ozone pollution | 0.427 | 0.054 | 0.478* | 0.029 |
| Residential radon | 0.462* | 0.035 | 0.461* | 0.035 |
| Lead exposure | 0.327 | 0.148 | 0.621** | 0.003 |
| Child underweight | 0.337 | 0.136 | 0.626** | 0.002 |
| Iron deficiency | 0.306 | 0.178 | 0.630** | 0.002 |
| High body-mass index | 0.332 | 0.141 | 0.746** | <0.001 |
| Diet low in fruits | 0.448* | 0.042 | 0.550** | 0.01 |
| Diet low in nuts and seeds | 0.302 | 0.184 | 0.569** | 0.007 |
| Diet low in fiber | 0.085 | 0.715 | 0.498* | 0.021 |
| Low physical activity | 0.342 | 0.129 | 0.692** | 0.001 |
| Occupational carcinogens | 0.147 | 0.525 | 0.577** | 0.006 |
| Occupational asthmagens | 0.472* | 0.031 | 0.657** | 0.001 |
| Occupational particulate matter, gases, and fumes | 0.474* | 0.03 | 0.676** | 0.001 |
| Occupational noise | 0.281 | 0.217 | 0.648** | 0.001 |
| Discontinued breastfeeding | 0.322 | 0.155 | 0.621** | 0.003 |
| Occupational exposure to benzene | 0.406 | 0.068 | 0.605** | 0.004 |
| Occupational exposure to beryllium | 0.413 | 0.062 | 0.558** | 0.009 |
| Occupational exposure to cadmium | 0.374 | 0.094 | 0.500* | 0.021 |
| Occupational exposure to chromium | 0.272 | 0.234 | 0.467* | 0.033 |
| Occupational exposure to diesel engine exhaust | 0.383 | 0.087 | 0.525* | 0.015 |
| Occupational exposure to nickel | 0.365 | 0.104 | 0.495* | 0.023 |
| Occupational exposure to silica | 0.444* | 0.044 | 0.615** | 0.003 |
| Occupational exposure to sulfuric acid | 0.296 | 0.192 | 0.500* | 0.021 |
| Occupational exposure to trichloroethylene | 0.339 | 0.133 | 0.727** | <0.001 |
| Child wasting | 0.157 | 0.496 | 0.487* | 0.025 |

**Supplementary Table 10:** GBD 2017 risk factor hierarchy and accompanying exposure definitions

|  | **Risk factors** | | | **Exposure definition** | **Theoretical minimum risk exposure level** |  |
| --- | --- | --- | --- | --- | --- | --- |
| **0** | **All** | | | **..** | **..** |  |
| **1** | **Environmental and occupational risks** | | | **..** | **..** |  |
| **2** |  | **Unsafe water, sanitation, and hand-washing** | | **..** | **..** |  |
| 3 |  | Unsafe water source | | Proportion of individuals with access to different water sources (unimproved, improved except piped, or piped water supply) and reported use of household water treatment methods (boiling or filtering, chlorinating or solar filtering, or no treatment) | All individuals have access to water from a piped water supply that is also boiled or filtered before drinking |  |
| 3 |  | Unsafe sanitation | | Proportion of individuals with access to different sanitation facilities (unimproved, improved except sewer, or sewer connection) | All individuals have access to toilets with sewer connection |  |
| 3 |  | No access to hand-washing facility | | Proportion of individuals with access to handwashing facility with soap, water, and wash station | All individuals have access to handwashing facility with soap, water, and wash station |  |
| **2** | **Air pollution** | | | **..** | **..** |  |
| 3 | Particulate matter pollution | | | .. | .. |  |
| 4 |  | Ambient particulate matter pollution | | Annual average daily exposure to outdoor air concentrations of particulate matter with an aerodynamic diameter of ≤2·5 μm (PM2·5), measured in μg/m3 | Joint theoretical minimum risk exposure level for both household and ambient particulate matter pollution is a uniform distribution between 2·4 and 5·9 μg/m3, with burden attributed proportionally between household and particulate matter pollution on the basis of source of PM2·5 exposure in excess of theoretical minimum risk exposure level |  |
| 4 |  | Household air pollution from solid fuels | | Individual exposure to PM2·5 due to use of solid cooking fuel | See ambient particulate matter pollution |  |
| 3 | Ambient ozone pollution | | | Seasonal (6-month period with highest ozone) 8-h daily maximum ozone concentrations, measured in ppb | Uniform distribution between 29·1 and 35·7 ppb |  |
| **2** | **Other environmental risks** | | | **..** | **..** |  |
| 3 | Residential radon | | | Average daily exposure to indoor air radon levels measured in becquerels (radon disintegrations per second) per cubic metre (Bq/m3) | 10 Bq/m3, corresponding to the outdoor concentration of radon |  |
| 3 | Lead exposure | | | Blood lead levels in μg/dL of blood, bone lead levels in μg/g of bone | 2 μg/dL, corresponding to lead levels in pre-industrial humans as natural sources of lead prevent the feasibility of zero exposure |  |
| **2** |  | **Occupational risks** | | **..** | **..** |  |
| 3 |  | Occupational carcinogens | | .. | .. |  |
| 4 |  |  | Occupational exposure to asbestos | Proportion of the population with cumulative lifetime exposure to occupational asbestos | No occupational exposure to asbestos | |
| 4 |  |  | Occupational exposure to arsenic | Proportion of the population ever exposed to arsenic at work or through their occupation | No occupational exposure to arsenic | |
| 4 |  |  | Occupational exposure to benzene | Proportion of the population ever exposed to benzene at work or through their occupation | No occupational exposure to benzene | |
| 4 |  |  | Occupational exposure to beryllium | Proportion of the population ever exposed to beryllium at work or through their occupation | No occupational exposure to beryllium | |
| 4 |  |  | Occupational exposure to cadmium | Proportion of the population ever exposed to cadmium at work or through their occupation | No occupational exposure to cadmium | |
| 4 |  |  | Occupational exposure to chromium | Proportion of the population ever exposed to chromium at work or through their occupation | No occupational exposure to chromium | |
| 4 |  |  | Occupational exposure to diesel engine exhaust | Proportion of the population ever exposed to diesel engine exhaust at work or through their occupation | No occupational exposure to diesel engine exhaust | |
| 4 |  |  | Occupational exposure to formaldehyde | Proportion of the population ever exposed to formaldehyde at work or through their occupation | No occupational exposure to formaldehyde | |
| 4 |  |  | Occupational exposure to nickel | Proportion of the population ever exposed to nickel at work or through their occupation | No occupational exposure to nickel | |
| 4 |  |  | Occupational exposure to polycyclic aromatic hydrocarbons | Proportion of the population ever exposed to polycyclic aromatic hydrocarbons at work or through their occupation | No occupational exposure to polycyclic aromatic hydrocarbons | |
| 4 |  |  | Occupational exposure to silica | Proportion of the population ever exposed to silica at work or through their occupation | No occupational exposure to silica | |
| 4 |  |  | Occupational exposure to sulphuric acid | Proportion of the population ever exposed to sulphuric acid at work or through their occupation | No occupational exposure to sulphuric acid | |
| 4 |  |  | Occupational exposure to trichloroethylene | Proportion of the population ever exposed to trichloroethylene at work or through their occupation | No occupational exposure to trichloroethylene | |
| 3 |  | Occupational asthmagens | | Proportion of the population currently exposed to asthmagens at work or through their occupation | Background asthmagen exposures |  |
| 3 |  | Occupational particulate matter, gases, and fumes | | Proportion of the population ever exposed to particulates, gases, or fumes at work or through their occupation | No occupational exposure to particulates, gases, or fumes |  |
| 3 |  | Occupational noise | | Proportion of the population ever exposed to noise greater than 85 decibels at work or through their occupation | Background noise exposure |  |
| 3 |  | Occupational injuries | | Proportion of the population at risk to injuries related to work or through their occupation | The rate of injury deaths per 100 000 person-years is zero |  |
| 3 |  | Occupational ergonomic factors | | Proportion of the population who are exposed to ergonomic risk factors for low back pain at work or through their occupation | All individuals have the ergonomic factors of clerical and related workers |  |
| **1** | **Behavioural risks** | | | **..** | **..** |  |
| **2** |  | **Child and maternal malnutrition** | | **..** | **..** |  |
| 3 |  | Suboptimal breastfeeding | | .. | .. |  |
| 4 |  |  | Non-exclusive breastfeeding | Proportion of children younger than 6 months who receive predominant, partial, or no breastfeeding | All children are exclusively breastfed for first 6 months of life | |
| 4 |  |  | Discontinued breastfeeding | Proportion of children aged 6–23 months who do not receive any breast milk | All children continue to receive breast milk until 2 years of age | |
| 3 |  | Child growth failure | | .. | .. |  |
| 4 |  |  | Child underweight | Proportion of children ≥3 SDs, 2–3 SDs, and 1–2 SDs lower than the WHO 2006 standard weight-for-age curve | All children are <1 SD below the WHO 2006 standard weight-for-age curve | |
| 4 |  |  | Child wasting | Proportion of children ≥3 SDs, 2–3 SDs, and 1–2 SDs lower than the WHO 2006 standard weight-for-length curve | All children are <1 SD below the WHO 2006 standard weight-for-height curve | |
| 4 |  |  | Child stunting | Proportion of children ≥3 SDs, 2–3 SDs, and 1–2 SDs lower than the WHO 2006 standard height-for-age curve | All children are <1 SD below the WHO 2006 standard height-for-age curve | |
| 3 |  | Low birthweight and short gestation | | .. | .. |  |
| 4 |  |  | Low birthweight for gestation | Proportion of births occurring in 2-week gestational age categories from [0–24) weeks to [40–42) weeks, for each 500-g birthweight category starting from [0–500) g to [4000–4500) g[*](https://www.ncbi.nlm.nih.gov/pmc/articles/PMC6227755/table/tbl1/?report=objectonly#tbl1fn1) | 500-g birthweight category with lowest risk within each gestational age category | |
| 4 |  |  | Short gestation for birthweight | Proportion of births occurring in 500-g birthweight categories from [0–500) g to [4000–4500) g, for each 2-week gestational age category starting from [0–24) weeks to [40–42) weeks[*](https://www.ncbi.nlm.nih.gov/pmc/articles/PMC6227755/table/tbl1/?report=objectonly#tbl1fn1) | 2-week gestational age category with lowest risk within each birthweight category | |
| 3 |  | Iron deficiency | | Peripheral blood haemoglobin concentration in g/L for all iron-responsive causes | Counterfactual haemoglobin concentration in the absence of iron deficiency in g/L for all iron-responsive causes |  |
| 3 |  | Vitamin A deficiency | | Proportion of children aged 0–5 years with serum retinol concentration <0·7 μmol/L | No childhood vitamin A deficiency |  |
| 3 |  | Zinc deficiency | | Proportion of the population with inadequate zinc intake versus loss | No inadequate zinc intake |  |
| **2** |  | **Tobacco** | | **..** | **..** |  |
| 3 |  | Smoking | | Prevalence of current use of any smoked tobacco product and prevalence of former use of any smoked tobacco product; among current smokers, cigarette equivalents smoked per smoker per day and cumulative pack-years of exposure; among former smokers, number of years since quitting | All individuals are lifelong non-smokers |  |
| 3 |  | Chewing tobacco | | Current use of any chewing tobacco product | All individuals are lifelong non-users of chewing tobacco products |  |
| 3 |  | Second-hand smoke | | Average daily exposure to air particulate matter from second-hand smoke with an aerodynamic diameter smaller than 2·5 μg, measured in μg/m3, among non-smokers | No second-hand smoke exposure |  |
| **2** |  | **Alcohol use** | | **Average daily alcohol consumption of pure alcohol (measured in g per day) in current drinkers who had consumed alcohol during the past 12 months** | **Estimated distribution 0–10 g per day** |  |
| **2** |  | **Drug use** | | **Proportion of the population dependent upon opioids, cannabis, cocaine, or amphetamines; proportion of the population who have ever injected drugs** | **No drug use** |  |
| **2** |  | **Dietary risks** | | **..** | **..** |  |
| 3 |  | Diet low in fruits | | Average daily consumption of fruits (fresh, frozen, cooked, canned, or dried, excluding fruit juices and salted or pickled fruits) | Consumption of fruit 200–300 g per day |  |
| 3 |  | Diet low in vegetables | | Average daily consumption of vegetables (fresh, frozen, cooked, canned, or dried, excluding legumes and salted or pickled vegetables, juices, nuts and seeds, and starchy vegetables such as potatoes or corn) | Consumption of vegetables 290–430 g per day |  |
| 3 |  | Diet low in legumes | | Average daily consumption of legumes (fresh, frozen, cooked, canned, or dried legumes) | Consumption of legumes 50–70 g per day |  |
| 3 |  | Diet low in whole grains | | Average daily consumption of whole grains (bran, germ, and endosperm in their natural proportion) from breakfast cereals, bread, rice, pasta, biscuits, muffins, tortillas, pancakes, and other sources | Consumption of whole grains 100–150 g per day |  |
| 3 |  | Diet low in nuts and seeds | | Average daily consumption of nut and seed foods | Consumption of nuts and seeds 16–25 g per day |  |
| 3 |  | Diet low in milk | | Average daily consumption of milk, including non-fat, low-fat, and full-fat milk, excluding soy milk and other plant derivatives | Consumption of milk 350–520 g per day |  |
| 3 |  | Diet high in red meat | | Average daily consumption of red meat (beef, pork, lamb, and goat but excluding poultry, fish, eggs, and all processed meats) | Consumption of red meat 18–27 g per day |  |
| 3 |  | Diet high in processed meat | | Average daily consumption of meat preserved by smoking, curing, salting, or addition of chemical preservatives | Consumption of processed meat 0–4 g per day |  |
| 3 |  | Diet high in sugar-sweetened beverages | | Average daily consumption of beverages with ≥50 kcal per 226·8 g serving, including carbonated beverages, sodas, energy drinks, fruit drinks, but excluding 100 fruit and vegetable juices | Consumption of sugar-sweetened beverages 0–5 g per day |  |
| 3 |  | Diet low in fibre | | Average daily intake of fibre from all sources including fruits, vegetables, grains, legumes, and pulses | Consumption of fibre 19–28 g per day |  |
| 3 |  | Diet low in calcium | | Average daily intake of calcium from all sources, including milk, yogurt, and cheese | Consumption of calcium 1·0–1·5 g per day |  |
| 3 |  | Diet low in seafood omega 3 fatty acids | | Average daily intake of eicosapentaenoic acid and docosahexaenoic acid | Consumption of seafood omega 3 fatty acids 200–300 mg per day |  |
| 3 |  | Diet low in polyunsaturated fatty acids | | Average daily intake of omega 6 fatty acids from all sources, mainly liquid vegetable oils, including soybean oil, corn oil, and safflower oil | Consumption of polyunsaturated fatty acids as 9–13 of total daily energy |  |
| 3 |  | Diet high in trans fatty acids | | Average daily intake of trans fat from all sources, mainly partially hydrogenated vegetable oils and ruminant products | Consumption of trans fatty acids as 0–1 of total daily energy |  |
| 3 |  | Diet high in sodium | | 24-h urinary sodium measured in g per day | 24-h urinary sodium 1–5 g per day |  |
| **2** |  | **Intimate partner violence** | | **Proportion of the population who have ever experienced one or more acts of physical or sexual violence by a present or former intimate partner since age 15 years** | **No intimate partner violence** |  |
| **2** |  | **Childhood maltreatment** | | **..** | **..** |  |
| 3 |  | Childhood sexual abuse | | Proportion of the population ever having had the experience of intercourse or other contact abuse (ie, fondling and other sexual touching) when aged 15 years or younger, and the perpetrator or partner was more than 5 years older than the victim | No childhood sexual abuse |  |
| 3 |  | Bullying victimisation | | Proportion of population attending school who have been exposed to bullying victimisation within the past year | No bullying victimisation |  |
| **2** |  | **Unsafe sex** | | **Proportion of the population with exposure to sexual encounters that convey the risk of disease** | **No exposure to disease-causing pathogen through sex** |  |
| **2** |  | **Low physical activity** | | **Average weekly physical activity at work, home, transport-related and recreational measured by MET min per week** | **All adults experience 3000–4500 MET min per week** |  |
| **1** | **Metabolic risks** | | | **..** | **..** |  |
| **2** |  | **High fasting plasma glucose** | | **Serum fasting plasma glucose measured in mmol/L** | **4·8–5·4 mmol/L** |  |
| **2** |  | **High low-density lipoprotein cholesterol** | | **Serum low-density lipoprotein, measured in mmol/L** | **0·7–1·3 mmol/L** |  |
| **2** |  | **High systolic blood pressure** | | **Systolic blood pressure, measured in mm Hg** | **110–115 mm Hg** |  |
| **2** |  | **High body-mass index** | | **Body-mass index, measured in kg/m2** | **20–25 kg/m2** |  |
| **2** |  | **Low bone mineral density** | | **Standardised mean bone mineral density values measured by dual x-ray absorptiometry at the femoral neck in g/cm2** | **99th percentile of NHANES 1988–2014 by age and sex** |  |
| **2** |  | **Impaired kidney function** | | **Proportion of the population with ACR >30 mg/g or GFR <60 mL/min/1·73 m2, excluding end-stage renal disease** | **GFR >60 mL/min/1·73 m2 and ACR <30 mg/g** |  |


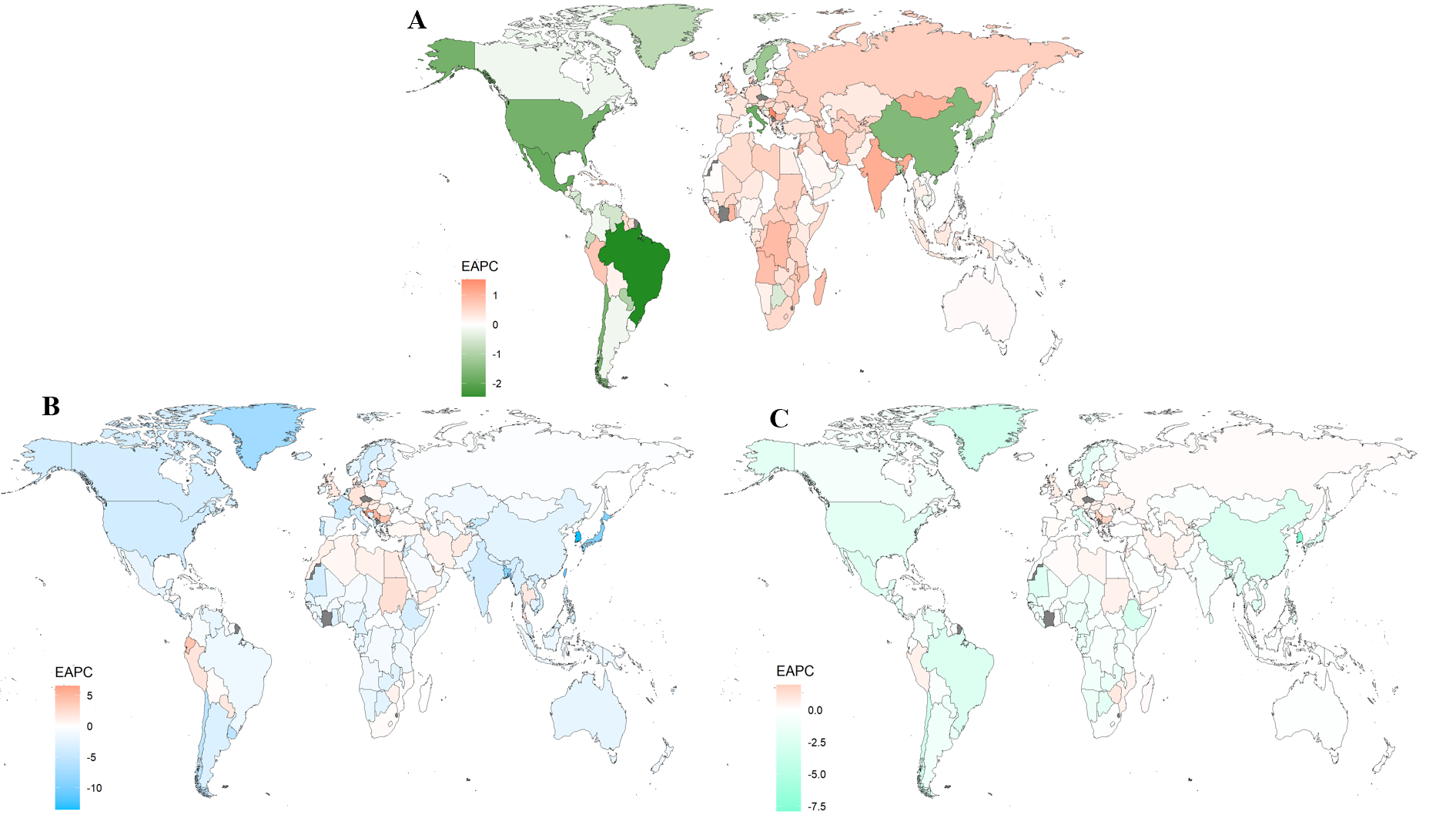


**Supplementary Figure1:** **EAPC of ASIR (A), ASDR(B) and AS-DALYs(C) in 1990 and 2019 for all locations from 1990 and 2019.**

**Note: All the world maps above were generated by open-source software R (version 4.3.1, http://www.r-project.org/)**


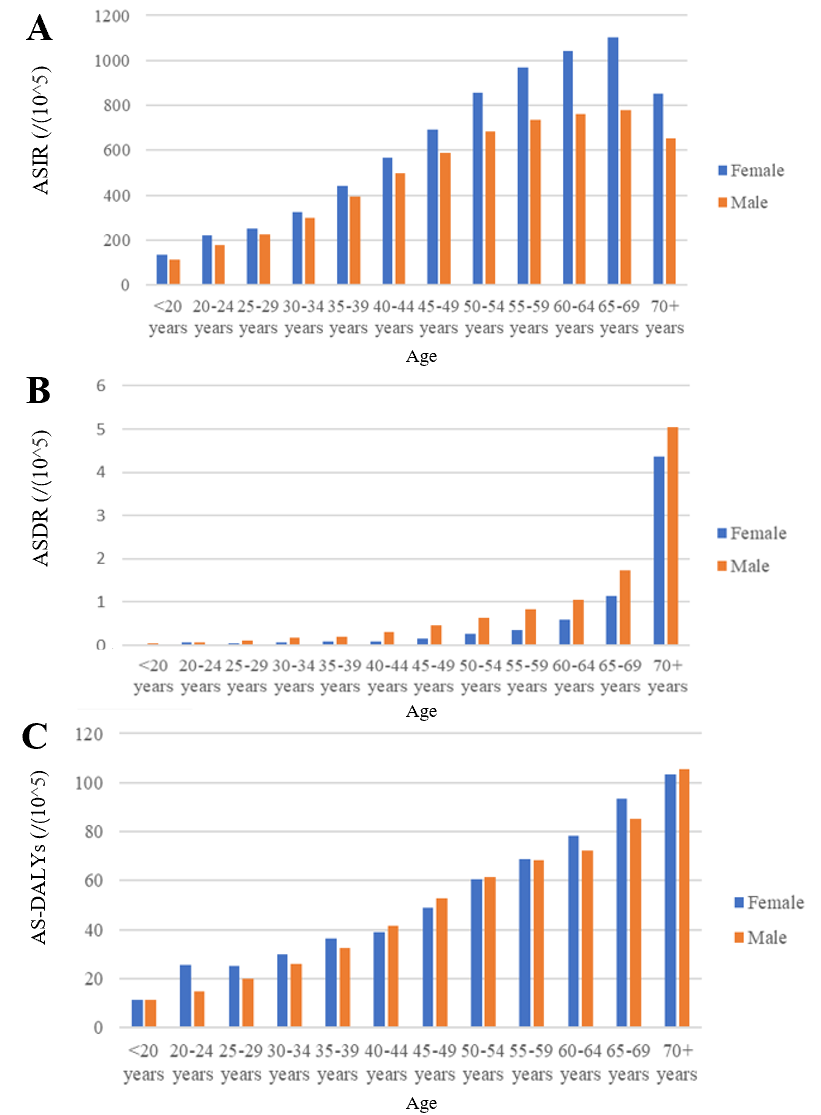


**Supplementary Figure2:GD related ASIR(A), ASDR(B) and AS-DALYs(C) of different gender in 2019**

**
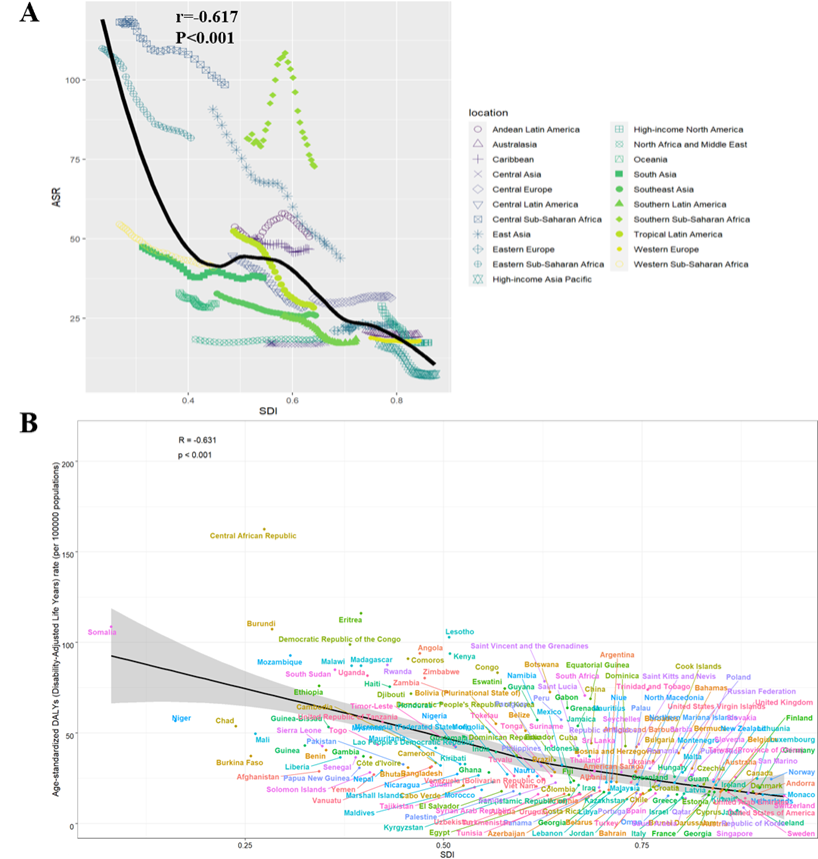
**

**Supplementary Figure3:** **(A)AS-DALYs for GD for different regions and (B) countries and territories by SDI, 1990–2019**


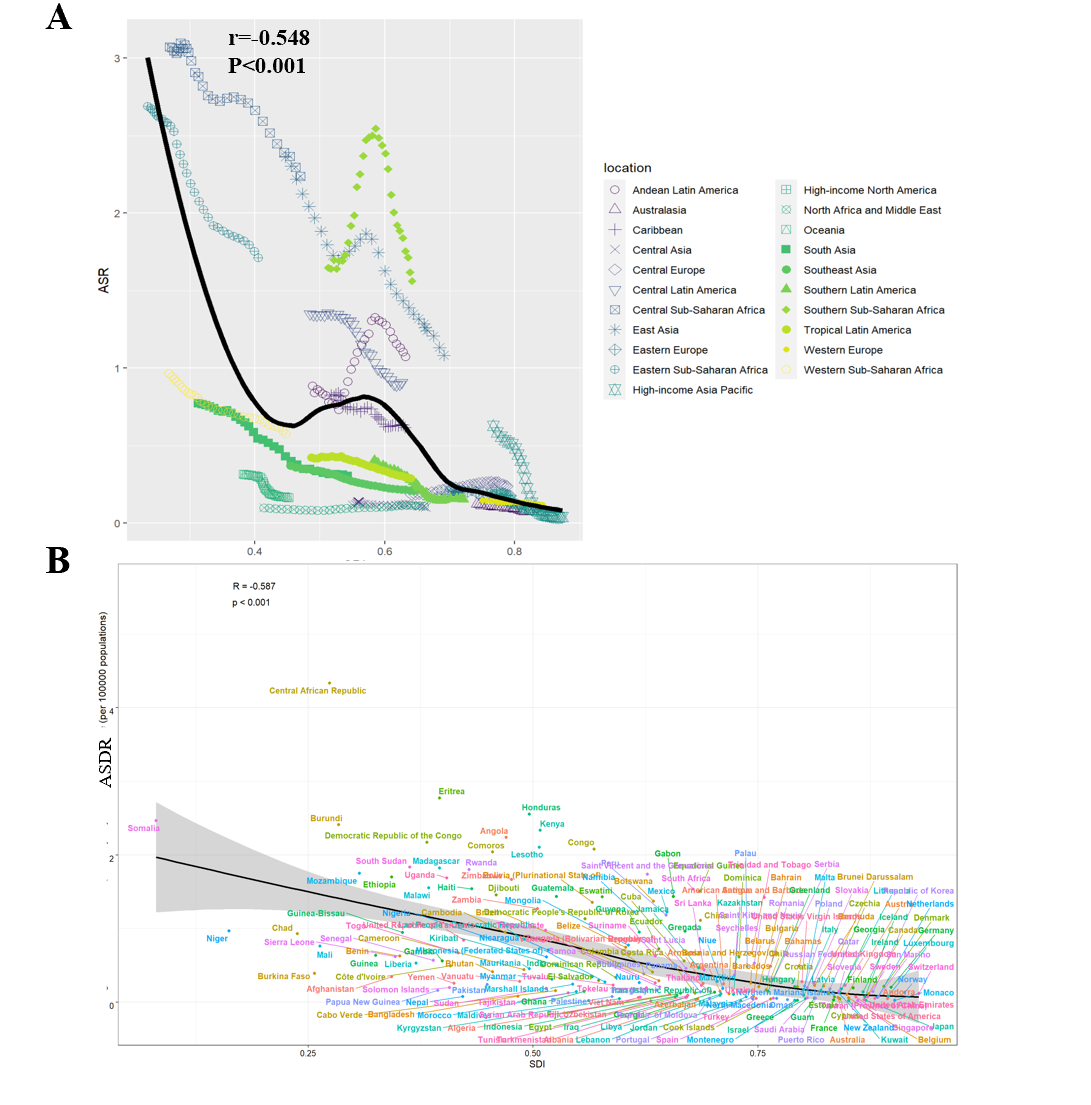


**Supplementary Figure4:** **(A) ASDR for GD for different regions and (B) countries and territories by SDI, 1990–2019**
